# Comparative Spectroscopic Study Revealing Why the CO_2_ Electroreduction Selectivity Switches from CO to HCOO^–^ at Cu–Sn- and Cu–In-Based Catalysts

**DOI:** 10.1021/acscatal.2c04419

**Published:** 2022-12-05

**Authors:** Gumaa A. El-Nagar, Fan Yang, Sasho Stojkovikj, Stefan Mebs, Siddharth Gupta, Ibbi Y. Ahmet, Holger Dau, Matthew T. Mayer

**Affiliations:** † Young Investigator Group Electrochemical Conversion of CO_2_, Helmholtz-Zentrum Berlin für Materialien und Energie GmbH, Hahn-Meitner-Platz 1, Berlin 14109, Germany; ‡ Department of Physics, Freie Universität Berlin, Arnimallee 14, Berlin 14195, Germany; § Institut für Chemie und Biochemie, Freie Universität Berlin, Arnimallee 22, Berlin D-14195, Germany; ∥ Institute for Solar Fuels, Helmholtz-Zentrum Berlin für Materialien und Energie GmbH, Hahn-Meitner-Platz 1, Berlin 14109, Germany; ⊥ Department of Chemistry, Faculty of Science, Cairo University, Giza 12613, Egypt

**Keywords:** CO_2_ electroreduction, Cu nanostructures, in situ spectroscopy, bimetallic catalysts, electrodeposition

## Abstract

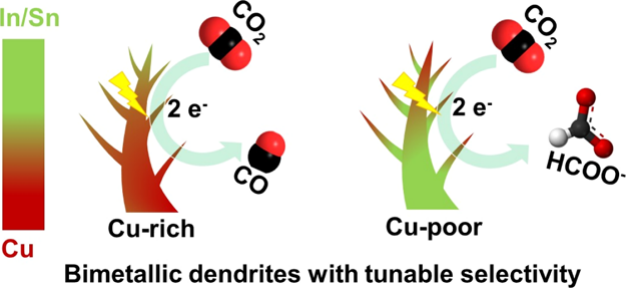

To address the challenge of selectivity toward single products in Cu-catalyzed electrochemical CO_2_ reduction, one strategy is to incorporate a second metal with the goal of tuning catalytic activity via synergy effects. In particular, catalysts based on Cu modified with post-transition metals (Sn or In) are known to reduce CO_2_ selectively to either CO or HCOO^–^ depending on their composition. However, it remains unclear exactly which factors induce this switch in reaction pathways and whether these two related bimetal combinations follow similar general structure–activity trends. To investigate these questions systematically, Cu–In and Cu–Sn bimetallic catalysts were synthesized across a range of composition ratios and studied in detail. Compositional and morphological control was achieved via a simple electrochemical synthesis approach. A combination of operando and quasi-in situ spectroscopic techniques, including X-ray photoelectron, X-ray absorption, and Raman spectroscopy, was used to observe the dynamic behaviors of the catalysts’ surface structure, composition, speciation, and local environment during CO_2_ electrolysis. The two systems exhibited similar selectivity dependency on their surface composition. Cu-rich catalysts produce mainly CO, while Cu-poor catalysts were found to mainly produce HCOO^–^. Despite these similarities, the speciation of Sn and In at the surface differed from each other and was found to be strongly dependent on the applied potential and the catalyst composition. For Cu-rich compositions optimized for CO production (Cu_85_In_15_ and Cu_85_Sn_15_), indium was present predominantly in the reduced metallic form (In^0^), whereas tin mainly existed as an oxidized species (Sn^2/4+^). Meanwhile, for the HCOO^–^-selective compositions (Cu_25_In_75_ and Cu_40_Sn_60_), the indium exclusively exhibited In^0^ regardless of the applied potential, while the tin was reduced to metallic (Sn^0^) only at the most negative applied potential, which corresponds to the best HCOO^–^ selectivity. Furthermore, while Cu_40_Sn_60_ enhances HCOO^–^ selectivity by inhibiting H_2_ evolution, Cu_25_In_75_ improves the HCOO^–^ selectivity at the expense of CO production. Due to these differences, we contend that identical mechanisms cannot be used to explain the behavior of these two bimetallic systems (Cu–In and Cu–Sn). Operando surface-enhanced Raman spectroscopy measurements provide direct evidence of the local alkalization and its impact on the dynamic transformation of oxidized Cu surface species (Cu_2_O/CuO) into a mixture of Cu(OH)_2_ and basic Cu carbonates [Cu_x_(OH)_y_(CO_3_)_y_] rather than metallic Cu under CO_2_ electrolysis. This study provides unique insights into the origin of the switch in selectivity between CO and HCOO^–^ pathways at Cu bimetallic catalysts and the nature of surface-active sites and key intermediates for both pathways.

## 1 Introduction

The use of CO_2_ as a carbon feedstock will be an important component of a sustainable post-fossil-fuel future. CO_2_ can be electrochemically reduced into a variety of products including hydrocarbons, alcohols, carbon monoxide (CO), and formate (HCOO^–^). Recent techno-economic analyses suggest that two-electron reduction of CO_2_ into C_1_ products (i.e., HCOO^–^ and CO) presents the best route to economic feasibility in the near future.^1−4^ CO and HCOO^–^ are essential feedstocks used in various industrial applications ranging from chemical synthesis (Fischer–Tropsch) to energy conversion (e.g., fuel cells) through existing and emerging technologies.^5−7^ The realization of electrochemical synthesis of these important molecules on a large scale will require catalysts with high activity and selectivity to the desired products, exceptional stability, sufficient earth abundance, and broad availability. New discoveries of improved catalysts, guided by a detailed understanding of structure–activity relationships, are needed in order to progress toward these targets.

The selectivity of CO_2_ electroreduction (CO_2_ER) is strongly dependent on the nature of the catalyst. Among the single metals which show CO_2_ER activity,^8^ In and Sn are mainly HCOO^–^ producers, while Cu shows a unique ability to reduce CO_2_ into a wide range of products including C_1_ compounds (CO, HCOO^–^, and CH_4_) and C_2+_ hydrocarbons (C_2_H_4_, C_2_H_5_OH, and others), typically as an undesirable mixture.^9,10^ A variety of approaches, including surface nanostructuring^11−13^ and tuning the electronic structure by modifying with a second metal, have been introduced to direct the selectivity of Cu toward a specific product.^10,14,15^ Several recent studies revealed that modulating the Cu surface with another metal, such as In or Sn, is an effective strategy to tune selectivity toward either CO or HCOO^–^ at relatively low overpotentials.^16,17^ Achieving these selectivities using earth-abundant metals presents a promising alternative to the use of more expensive or rare metals, such as Au and Ag which are highly selective toward CO. Luo et al.,^18^ Rasul et al.,^19^ and Zhu et al.^17^ reported high CO Faradaic efficiency (>85% CO FE) at Cu surfaces modified with In nanoparticles. On the other hand, other researchers^17,20^ developed Cu–In bimetallic electrocatalysts with high FE for HCOO^–^ (>85%). Many other studies revealed a similar behavior for Cu–Sn systems, where researchers reported Cu–Sn catalysts with high FE toward either CO or HCOO^–^.^7,16,21^ For instance, Li et al.^22^ investigated CO_2_ER on Cu/SnO_2_ core–shell nanoparticles with various Sn shell thicknesses, finding the selectivity to strongly depend on the said thickness, with thicker Sn shells showing Sn-like activity that is high HCOO^–^ selectivity (FE ∼ 90%), while thinner shells exhibited a high CO selectivity (FE ∼ 93%). Moreover, our recently published work^16^ reported a similar behavior for Cu nanowires coated with ultrathin SnO_*x*_ layers grown using atomic layer deposition, and detailed X-ray spectroscopic investigation revealing differing Sn speciation (i.e., oxidation state) between the CO- and HCOO^–^-selective catalysts.

In previous studies, the general trend is that Cu–In and Cu–Sn catalysts with low In and Sn contents (Cu-rich catalysts) are CO-selective while those with high Sn and In contents (Cu-poor catalysts) showed a higher tendency toward HCOO^–^. A variety of mechanistic hypotheses, such as alloying and electronic effects, have been proposed to explain these bimetallic synergetic effects, mostly centered around the crucial role of Cu–In and Cu–Sn interfaces in tuning the binding strength of the key intermediates (e.g., *COOH, *OCHO, and *H) and consequently CO and HCOO^–^ selectivity.^16,18,22−24^ For instance, density functional theory (DFT) calculations attribute the selectivity shifts (from CO to formate) with increase in In or Sn contents to the gradual weakening of the adsorbed *COOH intermediate (which leads to CO), concurrently with the enhancing adsorption of the *OCHO intermediate (resulting in HCOO^–^). A plausible explanation of this observed shift from carbophilic (*COOH, C-bound) to oxophilic (*OCHO, O-bound) adsorption modes could be the charge transfer from Sn or In to Cu sites, resulting in localized positive charge regions. These regions on the catalyst surface hamper the adsorption of the *COOH intermediate and hence increase the competitiveness of the HCOO^–^ production pathway at the expense of the CO formation pathway.^17,23^ These theoretical investigations into Cu–In and Cu–Sn suggest a direct correlation between the surface atoms environment (surface composition) and the surface electronic properties (charge distribution) and indicate their essentially inextricable role in the binding energy of the key intermediates for CO and HCOO^–^ production.

The surface composition and speciation of Cu-based bimetallic catalysts (Cu–Sn and Cu–In) have substantial effects on their performance (selectivity) for CO_2_ER, as discussed above. However, most of the previous studies build their mechanistic hypotheses based on structural information gathered from ex situ measurements conducted either before or after CO_2_ER testing. Since it is well known that catalyst materials can significantly transform under CO_2_ER measurement conditions, basing DFT calculations on such structural information as a main way to explain the observed selectivity trends of these bimetallic systems can be challenging and potentially misleading, as the models likely do not represent the precise active-surface species. Accordingly, in-depth exploration of the surface chemical environment, speciation (oxidation states), structure, and composition of these bimetallic systems using a combination of appropriate and complementary surface-sensitive techniques via operando/in situ configurations is required to fully expose the complex link between their composition, intermediate adsorption, and selectivity. As mentioned above, a limited number of studies have reported the high selectivity of Cu–In catalysts to either CO or HCOO^–^, but none of them, to the best of our knowledge, provides detailed insights into the active surface speciation. Furthermore, while one may presume that Cu–In and Cu–Sn systems follow the same mechanisms for selectivity tuning based on the similar composition–activity trends, to date, no study has systematically compared the two to address this possibility. Thus, there is a need for detailed and systematic investigations into the surface properties of such bimetallic catalysts to define the precise surface-active species, which govern their selectivity shift from CO to HCOO^–^.

This work is mainly dedicated to providing a thorough structure–composition–activity comparison between Cu–In and Cu–Sn bimetallic electrocatalysts using various surface- and bulk-sensitive X-ray spectroscopic techniques, aimed at providing insights into the open questions regarding the similarity and differences between these two systems. In this regard, multiple complementary techniques including quasi-in situ X-ray photoelectron spectroscopy (XPS) and X-ray absorption spectroscopy (XAS) combined with in situ surface-enhanced Raman spectroscopy (SERS) were used to probe the chemical environment and surface changes under CO_2_ER electrolysis. A simple one-pot electrochemical synthesis strategy was used to prepare bimetallic Cu–In and Cu–Sn with dendrite-like structures across a range of controlled In and Sn contents to tune their selectivity toward either CO or HCOO^–^. In situ SERS was used to probe the catalyst/electrolyte interface changes (e.g., local pH changes, surface oxide species) and key intermediates of CO_2_ reduction for a better understanding of the origin of the preference shift from the C-bound intermediate to the O-bound intermediate with increase in Sn/In surface contents. This comparative study highlights key differences in the surface composition and speciation (surface-active species) between Cu–In and Cu–Sn bimetallic systems under CO_2_ER conditions, despite both systems showing similar CO_2_ER catalytic activity behaviors. It also introduces new insights into the debated role of the persistence of metal surface oxide species and their dynamic transformations during CO_2_ER.

## 2 Experimental Section

Cu–Sn and Cu–In nanostructures of various compositions were grown onto Cu mesh substrates using a simple electrodeposition approach. In brief, Cu was co-electrodeposited simultaneously with either In (to create Cu–In) or Sn (to fabricate Cu–Sn) from 1.5 M H_2_SO_4_ solutions containing Cu/In or Cu/Sn salts, respectively, at different molar ratios by applying relatively high cathodic current (1 A·cm^–2^). Under these conditions, H_2_ bubbles generated in situ act as a dynamic template for constructing foam-like structures with mesoscale porosity. The morphology, structure, and composition of the as-prepared bimetallic materials were examined by various material characterization techniques. In situ SERS and glovebox-assisted XAS and XPS were used for tracking dynamic changes of CO_2_ER intermediates and local changes of the bimetallic surfaces and environment under CO_2_ER conditions. Gaseous and liquid products were quantified via gas chromatography and high-performance liquid chromatography. Comprehensive details of the materials, synthesis procedures, and characterization methods are provided in the Supporting Information.

## 3 Results and Discussion

### 3.1 Material Characterization

**Figure 1:**
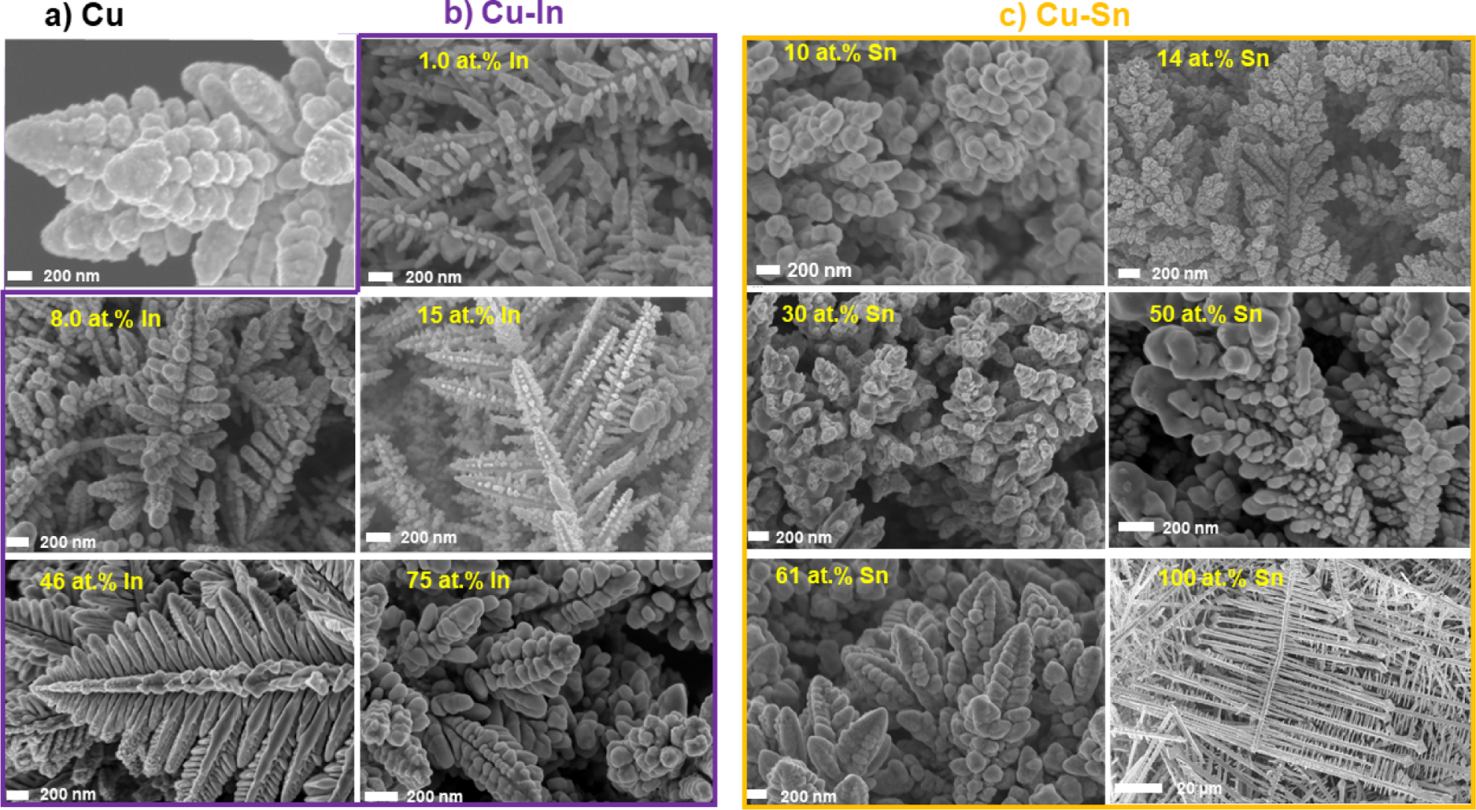
Representative SEM images of pure Cu (a) and bimetallic Cu–In [(b) violet border)] and Cu–Sn [(c) orange border)] dendrites with various In and Sn contents. In and Sn atomic percentages (at. %) are provided in the SEM images as estimated from their respective EDX analysis.

The physical properties, including the morphology, thickness, porosity, crystalline structure, and surface/bulk compositions, of the as-prepared Cu_x_Sn_y_ (*x* = at. % Cu, *y* = at. % Sn) and Cu_x_In_z_ (*z* = at. % In) bimetallic catalysts were first evaluated using different material characterization techniques. As shown in Figures 1, S1, and S2, porous Cu–Sn and Cu–In bimetallic foams with a range of compositions (various In and Sn contents) were successfully synthesized through a one-step co-electrodeposition synthesis approach, as described in the Experimental Section in the Supporting Information. Although the resulting materials exhibited similar dendrite-like microstructures, the fine microstructure of these dendrites showed some variation depending on the Cu/In and Cu/Sn molar ratios in the deposition solution (see Figures S1 and S2). The shape of these electrodeposited dendrites is mainly controlled by the rate of metal deposition and the rate of the concurrent hydrogen evolution reaction. The presence of either In or Sn in the Cu deposition bath is seen to have a significant impact on the nucleation, growth, and break-off rates of the in situ-generated hydrogen bubbles and hence on the fine structure and physical properties (e.g., porosity, thickness, etc.) of the obtained porous foams.^20^ The presence of Cu as a foaming agent is essential to create these dendritic microstructures (porous foams) since fabricated pure Sn and In did not show any dendrite-like structures. Contrary to the mixed metal depositions, electrodeposition from solutions of only Sn resulted in porous Sn films composed of interconnected Sn tubes (fishbone-like structures, Figure S1), while pure In exhibited a rough thin layer composed of In grains (Figure S2).

Grazing incidence X-ray diffraction (XRD) was next used to examine the crystalline structures of the as-prepared foams; data are displayed in Figure S3. Pure Cu foam exhibited mainly the typical reflection peaks for the cubic metallic copper with a small contribution from Cu_2_O, while the various as-prepared Cu–Sn bimetallic foams showed a mixture of metallic copper and Cu_2_O phases. Additionally, pure electrodeposited Sn exhibited several reflection peaks attributed to metallic Sn. No significant shifts are observed in the Cu reflection peaks of the all as-synthesized Cu–Sn foams compared to that of pure Cu. The foams with low Sn contents did not exhibit any noticeable signal for Sn (metallic or oxide), suggesting that electrodeposited Sn exists in an amorphous state or is below the detection threshold for XRD. Taken together, there is no clear evidence of the formation of crystalline Cu–Sn alloys. Cu–In bimetallic foams with a low In content (≤48 at. %) exhibited mainly reflection peaks for the metallic copper with small contribution of metallic In, while Cu–In foams with higher In contents beyond 48 at. % displayed a mixture of metallic In and Cu.

Again, the as-prepared Cu–In bimetallic foams did not show an obvious evidence of crystalline Cu–In alloy formation. It is worth noting that while the Cu-rich Cu–Sn foams exhibited very strong peaks for Cu_2_O with very low contribution of metallic Cu, the Cu-rich Cu–In foams showed only metallic Cu reflections. However, as described later in Section 3.2.1, XAS measurements under CO_2_ electrolysis conditions suggest the formation of Cu–In and Cu–Sn alloys, in addition to the existence of In and Sn oxides (see Section 3.2.2 for more details).

Furthermore, XPS (more surface-sensitive) analyses of these samples (air-exposed) showed the existence of a thin surface oxide shell for all the as-prepared Cu–In and Cu–Sn bimetallic catalysts (see Figure S4). We only measured XAS and XPS for the Cu–In and Cu–Sn bimetallic foams with the best CO_2_ER performance toward either CO or HCOO^–^.

### 3.2 CO_2_ER Performance

**Figure 2:**
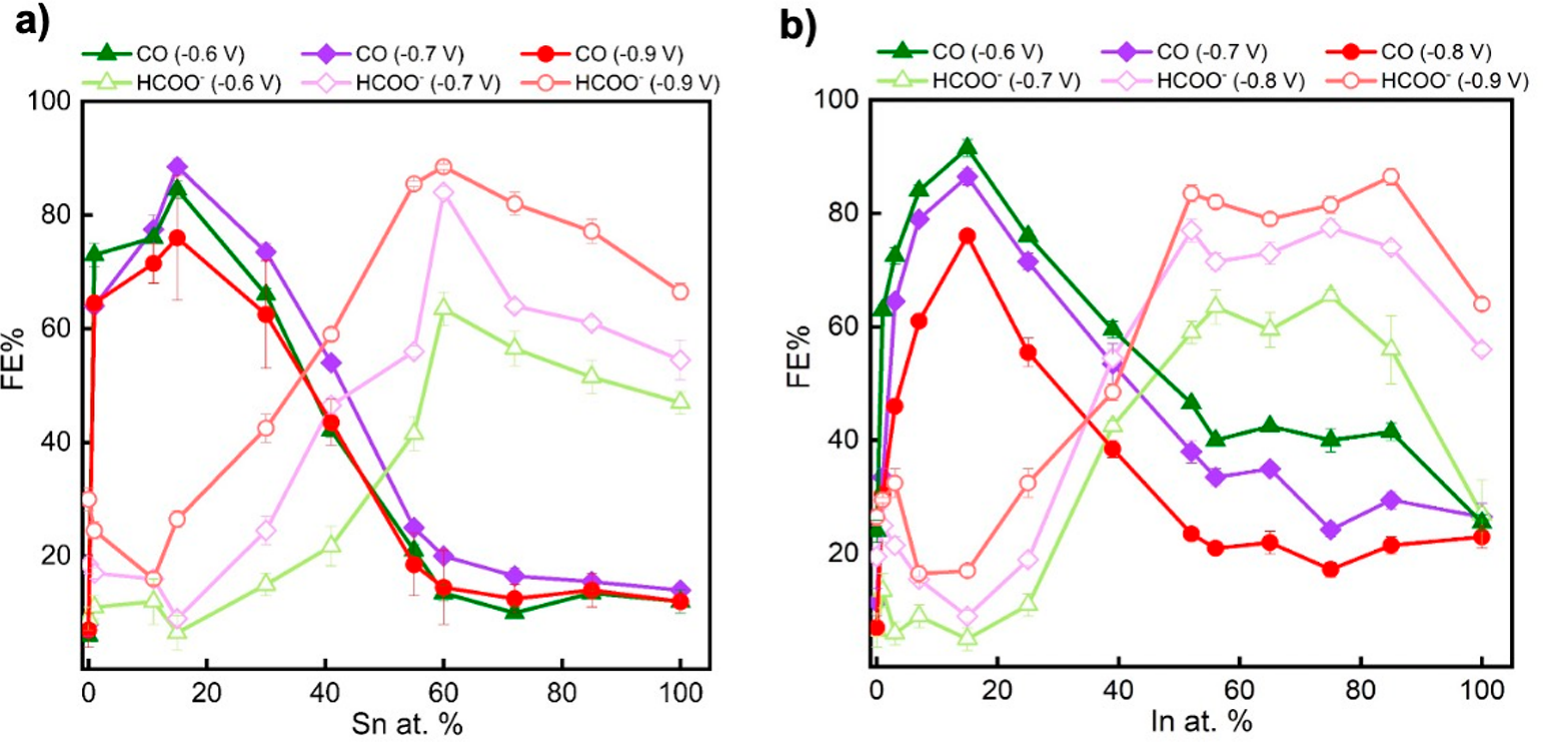
Distribution of CO and HCOO^–^ FE for Cu–Sn (a) and Cu–In (b) bimetallic foams as a function of Sn and In contents at several applied cathodic potentials. Each different *x*-axis value represents a separately prepared sample of different composition, tested over a range of potentials (indicated by different symbols/colors) and reporting the FE toward the major products CO (solid symbols) and HCOO^–^ (hollow symbols). The yields of H_2_ and other minor products are omitted for clarity. All results are expressed as average values ± average mean absolute errors from replicate samples.

The electrochemical CO_2_ reduction performance of the various as-prepared Cu–In and Cu–Sn bimetallic foams with a wide range of compositions was examined in CO_2_-saturated 0.1 M KHCO_3_ using a two-compartment, three-electrode H-type electrochemical cell. The obtained CO_2_ER performance results are summarized in Figures 2 and S5–S7. Figure 2 displays the FE of CO and HCOO^–^ formation as a function of Sn and In contents at various applied cathodic potentials. A similar trend in selectivity toward CO and HCOO^–^ pathways is observed for both the bimetallic foam systems, wherein Cu-rich bimetallic (Cu–In and Cu–Sn) foams showed a high CO selectivity, while Cu-poor foams exhibited a high tendency toward HCOO^–^ production. Even introducing trace amounts (≤1.5 at. %) of either Sn or In into Cu foam shifts its selectivity significantly toward CO production, and Sn or In contents of approximately 15 at. % resulted in peak CO selectivity reaching above 90%. Further increase in either metal results in an abrupt decrease in CO selectivity. Hereafter, Cu_85_In_15_ and Cu_85_Sn_15_, representing the foams with the highest CO selectivity, will be used for further detailed investigation. It is worth mentioning here that Cu_85_In_15_ foam exhibited its highest FE for CO of ∼92% at −0.6 V RHE, while Cu_85_Sn_15_ achieved a similar FE for CO but at more negative potential (−0.7 V RHE).

Further increase in the Sn and In contents leads to steadily increasing HCOO^–^ selectivity and plateauing for contents above 50–60 at. %, accompanied by greatly suppressed CO yields. Cu_40_Sn_60_ and Cu_25_In_75_ will be used henceforth for indicating the foams with the highest HCOO^–^ selectivity. Both displayed the best HCOO^–^ selectivity at −0.9 V RHE. Further detailed studies were only performed on the Cu–In and Cu–Sn foams with the best selectivity toward CO (Cu_85_In_15_ and Cu_85_Sn_15_) and HCOO^–^ (Cu_40_Sn_60_ and Cu_25_In_75_). Furthermore, Figure S4 shows the distribution of FE for H_2_ as a function of Sn and In contents (at. %) at various applied cathodic potentials. As shown in this figure, the selectivity of the competitive undesired hydrogen evolution reaction is significantly suppressed upon modifying Cu foams with even small amounts of either In or Sn.

**Figure 3:**
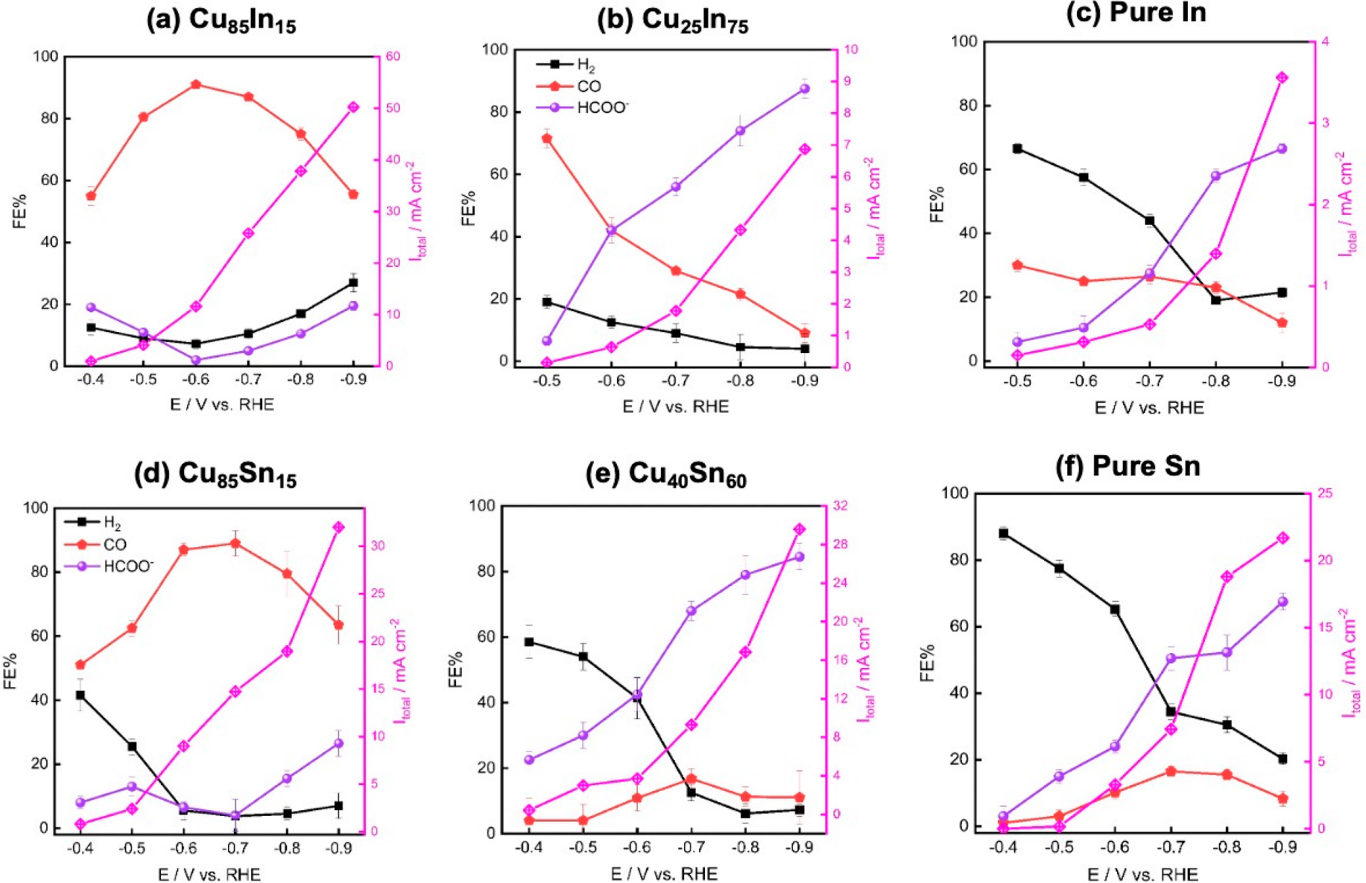
Potential-dependent FE distribution of major products (CO, HCOO^–^, and H_2_) and the obtained total current (*I*_total_, right *y*-axes) for bimetallic foam catalysts optimal for CO production [(a) Cu_85_In_15_ and (d) Cu_85_Sn_15_] and HCOO^–^ production [(b) Cu_25_In_75_ and (e) Cu_40_Sn_60_], compared to (c) pure In and (f) pure Sn. The relevant data of the pure Cu sample is provided in Figure S6. All results are expressed as average values ± average mean absolute errors from replicate samples.

Figures 3, S6, and S7 display the distribution of FEs and partial current densities of the different major products obtained at Cu–Sn and Cu–In bimetallic foams optimal for CO and HCOO^–^ production compared to their respective single elements at various applied cathodic potentials. Cu_25_In_75_ and Cu_40_Sn_60_ showed a similar steady increase in the HCOO^–^ selectivity with the increase in the applied potential (Figure 3a,e); however, they exhibited quite different selectivity trends for CO and H_2_. Cu_25_In_75_ showed low H_2_ selectivity with high CO selectivity at low potentials, while Cu_40_Sn_60_ in contrast exhibited high H_2_ selectivity with very low CO selectivity under similar conditions. On the other hand, the Cu-rich Cu–Sn and Cu–In bimetallic foams (Cu_85_In_15_ and Cu_85_Sn_15_) exhibited a kind of similar volcano-shaped trend for the CO selectivity with the maximum at −0.6 and −0.7 V versus RHE (all potentials given herein are relative to the RHE) for Cu_85_In_15_ and Cu_85_Sn_15_, respectively. Despite their similar CO selectivity behavior, they showed differences in the H_2_ formation behavior.

Cu_85_Sn_15_ foam showed a high tendency for H_2_ production at low potentials (<−0.6 V), while Cu_85_In_15_ exhibited a high CO selectivity (>50% CO FE) even at relatively lower overpotential (−0.4 V). The pure Sn and In catalysts followed similar CO, H_2_, and HCOO^–^ trends, where the HCOO^–^ selectivity increases with the potential accompanied with decrease in both H_2_ and CO production. However, pure In still shows higher CO and lower H_2_ selectivity at less negative potentials compared to pure Sn. Moreover, post-CO_2_ER scanning electron microscopy (SEM) analysis of Cu–In and Cu–Sn bimetallic dendrites with the best CO and HCOO^–^ activity showed the stability of their dendritic-like structures under CO_2_ER measuring conditions (Figure S8). More thorough analysis of the physical properties (e.g., porosity, thickness, and roughness) of the Cu–In and Cu–Sn bimetallic foams with the best CO and HCOO^–^ selectivity is essential for better understanding the wide variance in the observed currents and selectivity.^25,26^ Thus, the thickness and porosity of these bimetallic foams were estimated from their respective cross-section focused ion beam-SEM (see Figure S9). Their roughness and bulk and surface compositions were estimated using XPS (Figure S4), energy-dispersive X-ray spectroscopy (EDX) (Figures S10 and S11), and inductively coupled plasma–optical emission spectroscopy (ICP–OES); obtained results are summarized in Table 1.

**Table 1:** Summary of the Various Physical Parameters Including the Roughness, Thickness, Pore Size, and Morphology, in Addition to the Bulk and Surface Composition of the As-Synthesized Bimetallic Dendrites

	Cu/M (M = Sn or In) composition ratio						
catalyst	XPS (surface)	ICP–OES (bulk)	roughness^a^	thickness (μm)	average pore size (μm)	morphology	major product
Cu	N/A	N/A	173	64	28	foam/dendrites	H_2_ & C_2+_ products
Cu_85_In_15_	3.4	3.8	156	40	25	foam/dendrites	92% CO
Cu_85_Sn_15_	5.0	3.3	540	52	30	foam/dendrites	90% CO
Cu_25_In_75_	1.0	1.1	73	14	25	foam/dendrites	86% HCOO^–^
Cu_40_Sn_60_	0.04	0.02	204	16	36	foam/dendrites	87% HCOO^–^
Sn	N/A	N/A	320	N/A	N/A	foam/fishbone-like structures	70% HCOO^–^
In	N/A	N/A	10	N/A	N/A	rough porous layer	62% HCOO^–^

a Estimated by dividing *C*_dl_ of bimetallic by *C*_dl_ of the Cu mesh substrate.

Additionally, their electrochemically active surface area (roughness) was estimated via measurement of capacitive double layer behavior, as shown in Figure S12. Despite Cu_85_In_15_ (CO-selective) showing significantly lower electrochemically active surface area (see Table 1) compared to Cu_85_Sn_15_ (CO-selective), it exhibited higher CO_2_ER total current (Figure S6b). This indicates the higher intrinsic activity and the superiority of Cu_85_In_15_ over Cu_85_Sn_15_. In contrast, Cu_40_Sn_60_ (HCOO^–^-selective, Figure S7b) showed higher CO_2_ER current compared to Cu_25_In_75_ (Figure S6c) which could be attributed to its significantly higher electrochemically active surface area. So far, we have succeeded in fabricating and fully characterizing the Cu–In and Cu–Sn bimetallic dendrites with tunable performance toward either CO (Cu-rich dendrites) or HCOO^–^ (Cu-poor dendrites) production. The improved CO and HCOO^–^ selectivity on the bimetallic dendrites is not likely attributable to a bulk alloying effect since the obtained XRD patterns did not exhibit any signs of the existence of a crystalline alloy. Both bimetallic systems (Cu–In and Cu–Sn) showed a similar CO and HCOO^–^ selectivity dependence on In and Sn contents, where their HCOO^–^ selectivity increases with the applied potential with a simultaneous decrease in their tendency to produce CO. Despite their CO and HCOO^–^ selectivity trends, their enhanced ability to yield CO and HCOO^–^ could be attributed to different enhancing mechanisms. Cu_25_In_75_ showed a kind of linear increase in HCOO^–^ selectivity at the expense of CO, while Cu_40_Sn_60_ seems to enhance the HCOO^–^ selectivity at the expense of H_2_ production.

The synthetic method used herein allowed facile tuning of composition but indeed resulted in some morphological variation between the different compositions (Figure 1) which makes it challenging to precisely deconvolute the effects of chemical and morphological structures on the observed catalytic selectivity. Nonetheless, our characterization allows some degree of deconvolution. For example, upon comparing the CO-selective catalysts Cu_85_In_15_ and Cu_85_Sn_15_ to pure Cu (Table 1), we see that each has comparable average pore size and thickness of the dendritic layer. The fact that the bimetallic catalysts resulted in predominant CO production while hydrogen evolution reaction was suppressed, without significant change to the thickness and porosity as compared to Cu, suggests that morphology effects are not the dominant origin of the selectivity tuning in this case.

Although the above-mentioned ex situ characterization provides insights into the observed selectivity trends, it is known that electrocatalysts can transform significantly during electrochemical operation, and transition metals in particular can be susceptible to oxidation in air during post-run sample handling.

A more precise understanding of the true catalytic interface requires the use of in situ methods capable of characterizing the operating system. Thus, quasi-in situ XPS and XAS together with operando SERS were next used to examine the induced local environment (local pH) and surface (speciation and composition) changes during CO_2_ER at both bimetallic systems.

#### 3.2.1 Quasi-In Situ XPS and XAS Measurements

**Figure 4:**
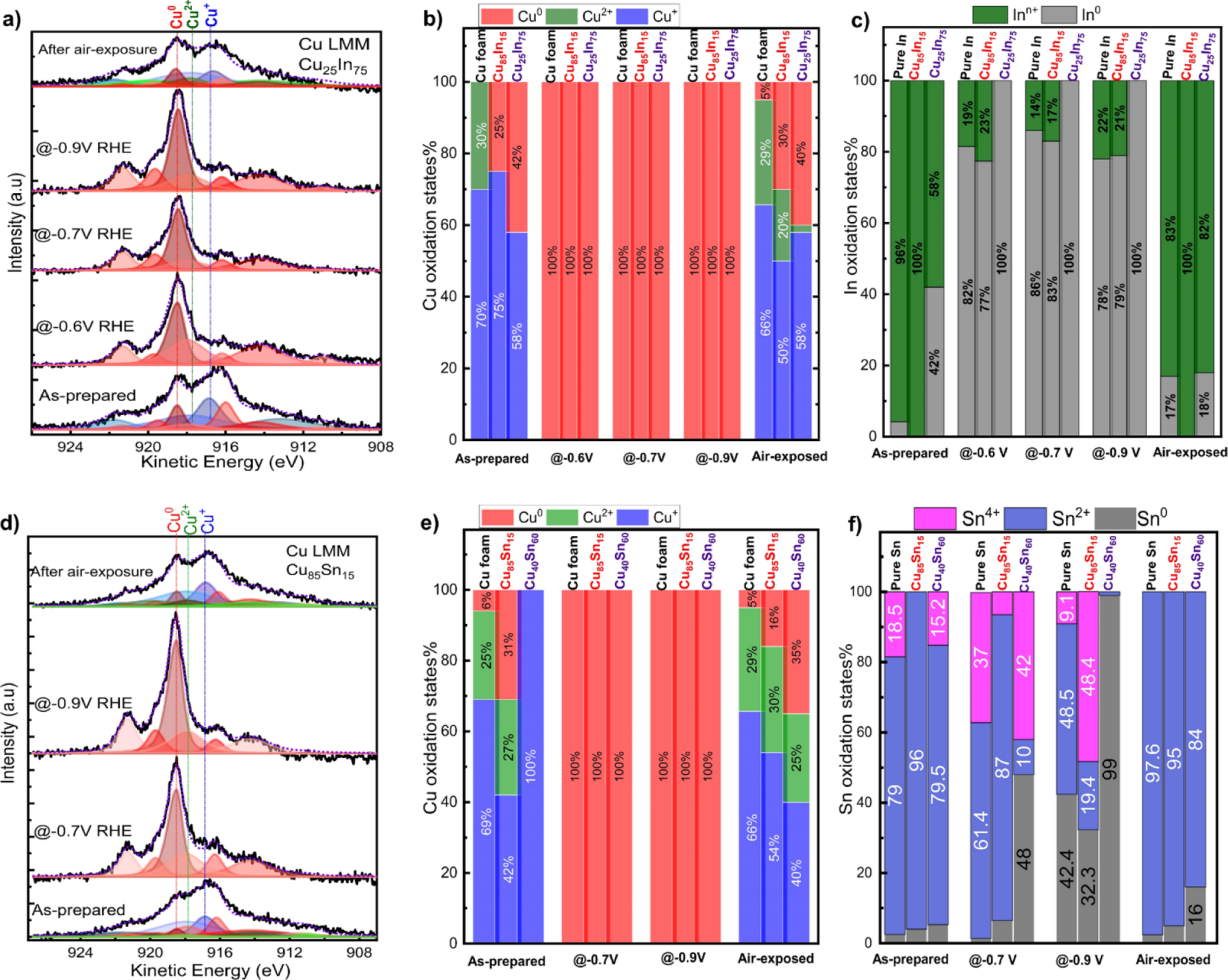
Quasi-in situ XPS for copper speciation based on Cu LMM Auger analysis. Representative Cu LMM Auger peak fittings (left column), fitting results of Cu surface speciation (middle) for Cu–In (a,b) and Cu–Sn (d,e) bimetallic foams following different ambient or electrochemical conditions, and summary of In and Sn surface speciation (right column)] of Cu–In (c) and Cu–Sn (f) bimetallic foams.

A so-called quasi-in situ XPS approach was used to explore the surface composition and speciation of the bimetallic systems giving the best CO and HCOO^–^ performance. CO_2_ electrolysis was carried out at various potentials in an O_2_-free glovebox under an inert (N_2_) atmosphere. Then, the measured samples were rapidly transferred to the XPS analysis chamber under vacuum without any air exposure through a gastight transfer capsule. In this way, we sought to avoid the re-oxidation of the tested catalysts’ surfaces, providing chemical and compositional insights more representatives of their real surface-active species. The quasi-in situ XPS results are summarized in Figures 4 and S13–S15. It is immediately noticeable that while the surface of the various as-prepared bimetallic foams and their single-metal counterparts (Cu, Sn, and In) is mostly dominated by oxide species (e.g., Cu_x_O_y_, SnO_x_, and In_x_O_y_) (Figure S4), the samples measured post-electrolysis resemble transformed surface speciation.

**Figure 5:**
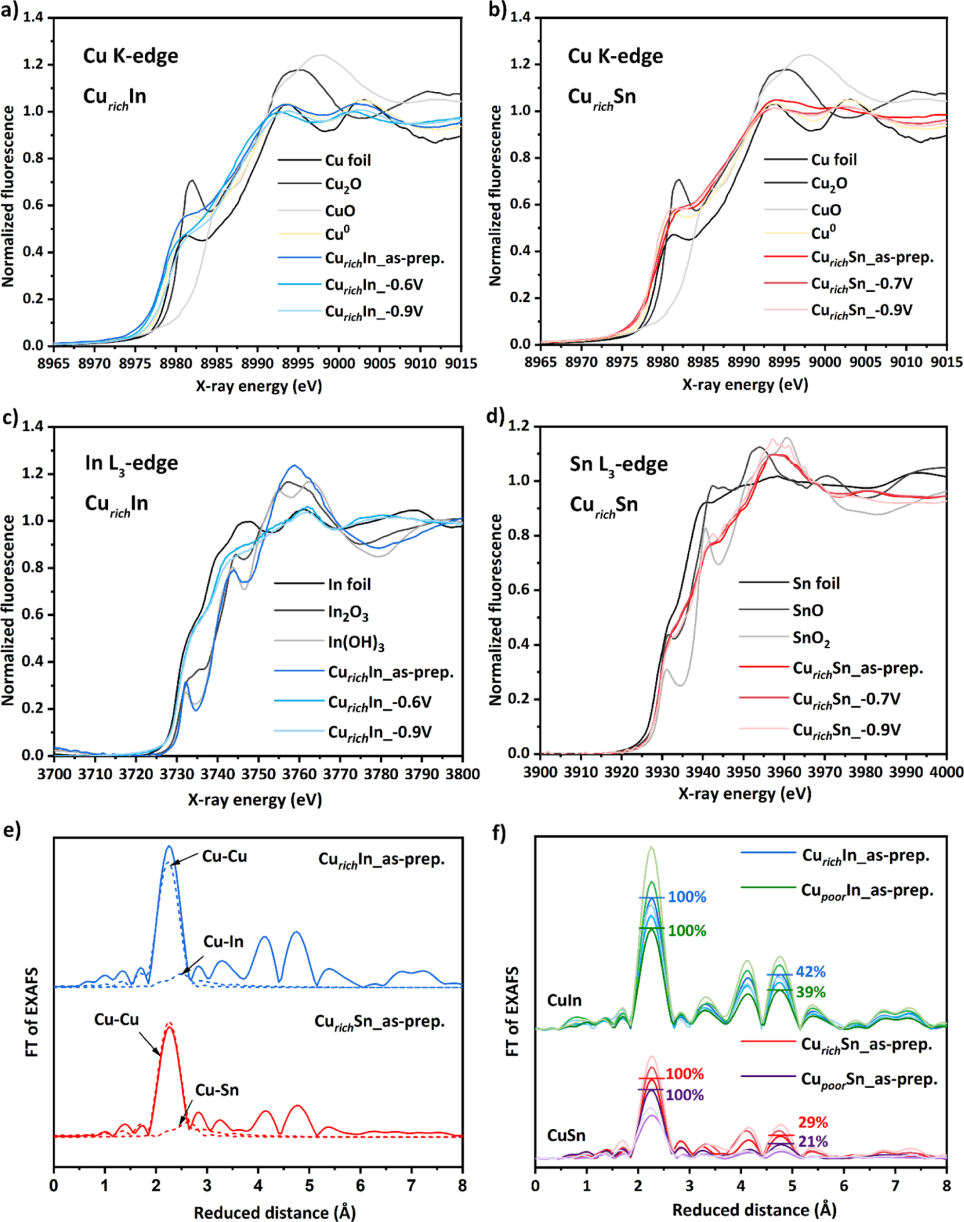
XAS analysis of Cu–In and Cu–Sn foams. (a,b) XANES at Cu K-edge for Cu-rich Cu–In and Cu–Sn foams under different electrochemical conditions. See inset legends. (c,d) Corresponding XANES at the In and Sn L_3_-edges. (e) FT of EXAFS (Cu K-edge) for as-prepared samples of Cu-rich CuIn and CuSn foam. Contributions of the first Cu shell and In or Sn shell are highlighted by dashed lines. (f) FT of EXAFS (Cu K-edge) for all Cu–In (upper spectra) and Cu–Sn (lower spectra) foams. Percentage numbers highlight the relative size of the first peak to the fourth peak for the as-prepared samples to convey different degrees of the long-range order. Light blue/green/red/purple colors refer to samples after application of reductive potentials; see the Supporting Information for details.

General observations based on our evaluation of the obtained Cu 2p spectra (see Figures S13 and S4) and Cu LMM Auger regions (see Figures 4, S15,and S5) are the following:

(I) Before CO_2_ER, the surface of all the as-synthesized materials, CO- and HCOO^–^-selective bimetallic foams, is predominated by Cu^+^ species (≥65%) with a small contribution from metallic Cu (≤20%) and Cu^2+^ (∼25%). It is worth mentioning that some of the as-synthesized catalyst compositions (Cu_85_In_15_, Cu_25_In_75_, and Cu_40_Sn_60_) did not display any Cu^2+^ surface species but exhibited mainly Cu^+^ species with a low metallic copper contribution, Figures S4 and S13.
(II) A complete transformation of the different detected surface copper oxide species (Cu^2+^ and Cu^+^) into metallic copper was observed for all investigated catalyst materials (i.e., Cu, Cu–In, and Cu–Sn foams) following CO_2_ER in the glovebox and the subsequent transfer to the XPS analysis chamber under inert atmosphere. This was true regardless of the applied cathodic potential (−0.5 to −0.9 V) and the different bimetallic compositions studied. Additionally, brief exposure of any of these catalysts to air resulted in significant re-oxidation of their surface into various copper oxide species (Cu^+^ and Cu^2+^) with small contribution of metallic copper, as shown in Figures 4 and S13 and S14.

The Cu LMM Auger analysis revealed the surface transformation to metallic Cu^0^ without any detectable residual oxides following electrolysis and furthermore showed how the as-prepared materials and the post-electrolysis samples exposed to air undergo significant surface re-oxidation. Taken together, these observations help validate the usefulness of the quasi-in situ XPS approach and highlight the limitations of using *ex situ* approaches to study electrocatalyst surfaces. The obtained Cu results emphasize the existence of the mixed heterogeneous surface of separate metals, rather than alloying since we did not detect any meaningful shift of the Cu 2p spectra of the bimetallic systems compared to Cu 2p of pure Cu. Unlike the Cu surface species, the analysis of the In 3d and Sn 3d spectra of bimetallic foams showed that the identified In and Sn surface species are strongly dependent on the initial composition and the applied potentials (Figures 4 and S15). CO-selective bimetallic foams (Cu-rich, Cu_85_In_15_ and Cu_85_Sn_15_) showed different surface speciation compared to HCOO^–^-selective catalysts (Cu-poor, Cu_25_In_75_ and Cu_40_Sn_60_). Before CO_2_ electrolysis, CO-selective bimetallic foams and their respective single elements (In and Sn) exhibited nearly fully oxidized In and Sn surface species with a tiny contribution (<4%) of metallic species (In^0^ and Sn^0^). Looking first at the Cu–In catalysts (Figures 4c and S15a–d), we see that after CO_2_ electrolysis at −0.5 V (or more negative potentials), the oxidized In surface of the pure In and Cu_85_In_15_ catalysts is severely reduced, where the quantification of their surfaces revealed the predominance of metallic In (77–86% In^0^) with a small contribution of oxidized In species (23–14% In^n+^).

On the other hand, Cu_25_In_75_ (HCOO^–^-selective catalysts) exhibited a mixture of metallic (58% In^0^) and oxide (42% In^n+^) surface species before CO_2_ER. Interestingly, Cu_25_In_75_ exhibited exclusively metallic In after CO_2_ electrolysis at all studied potentials (−0.5 to −0.9 V RHE). In summary, the obtained XPS results of Cu–In bimetallic foams reveal that the active surface of Cu_85_In_15_ foam is composed of metallic Cu^0^ and a mixture of metallic In^0^ (predominant species) and oxidized In (In^n+^), while the HCOO^–^-selective Cu_25_In_75_ surface is exclusively composed of Cu^0^ and In^0^.

The quantification of Sn surface speciation of CO- and HCOO^–^-selective Cu–Sn bimetallic dendrites (Figures 4h and S15e–h) displayed substantial differences compared to the Cu–In bimetallic dendrites under similar measuring conditions. Before CO_2_ER, the surface of the as-synthesized pure Sn, Cu_85_Sn_15_, and Cu_40_Sn_60_ foams exhibited predominantly oxidized Sn (∼95% Sn^*n*+^) with a residual of metallic Sn (Sn^0^). After CO_2_ER at −0.7 V, no major changes were detected in the Sn surface specification of Cu_85_Sn_15_ (CO-selective) catalysts, where their surfaces are predominantly composed of oxidized Sn species (∼87% Sn^2+^ and 7% Sn^4+^). Interestingly, at −0.9 V RHE, Sn^2+^ gave way to significant increases in Sn^0^ and Sn^4+^ species. Despite the high cathodic applied potential, the developed local high pH (cf Figure S31) can still result in oxidized Sn species. On the other hand, the HCOO^–^-selective Cu–Sn sample (Cu_40_Sn_60_) showed a great increase in the metallic Sn contents after CO_2_ electrolysis at −0.7 and −0.9 V RHE, where Sn^0^ increased from 4% before CO_2_ER to 48 and ∼100% at −0.7 and −0.9 V, respectively. Additionally, the brief air exposure of any of these catalysts leads to a near-complete re-oxidation of their surfaces, highlighting again the effectiveness of using glovebox-assisted XPS for identifying the surface-active species. Figure S16 summarizes surface composition and speciation of all the investigated electrodes including CO-selective and HCOO^–^-selective bimetallic Cu–In and Cu–Sn foams.

**Scheme 1:**
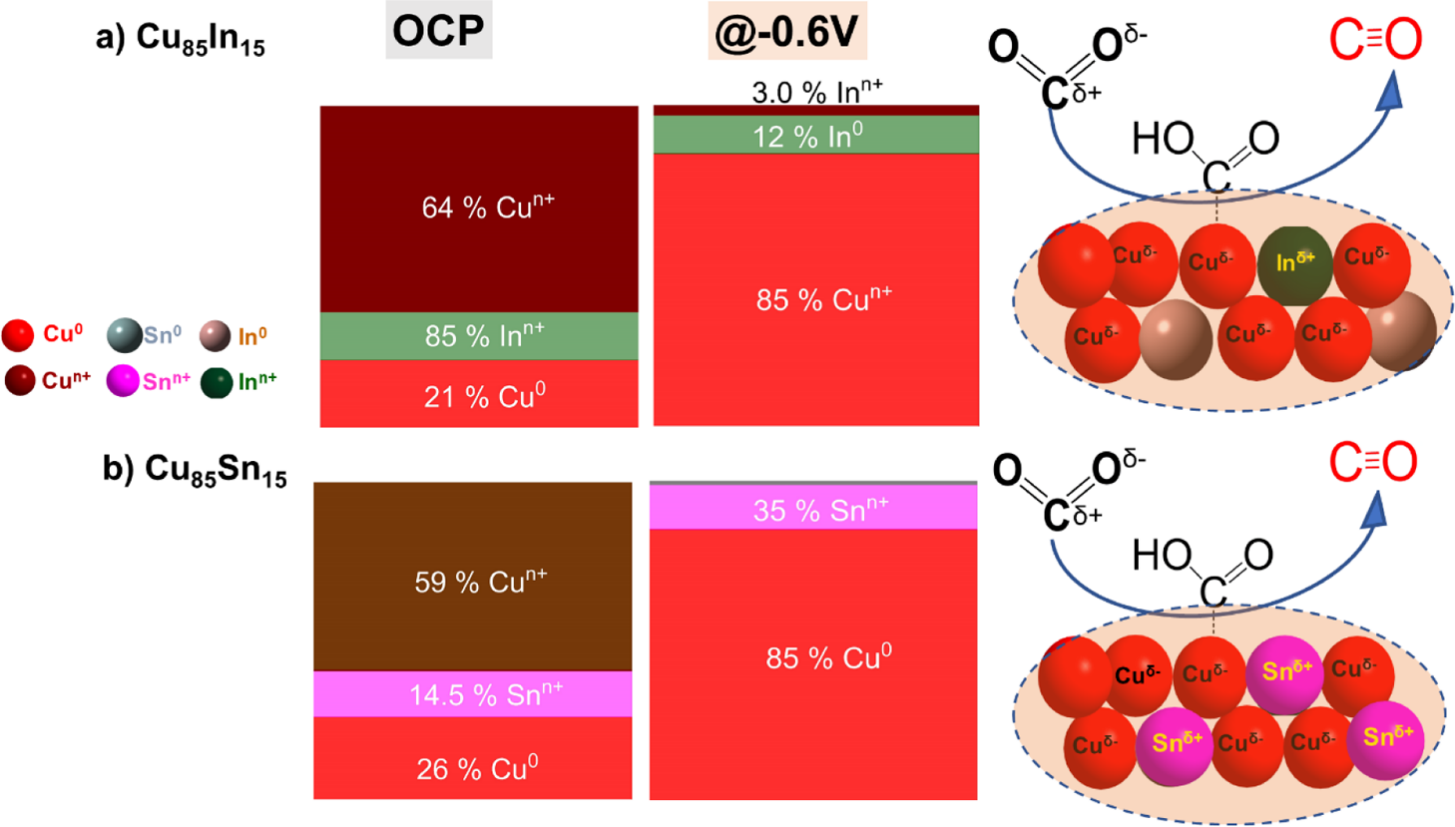
Schematic Diagram Showing the Surface-Active Sites of CO-Selective Bimetallic Catalysts and the Role of the Speciation in Enhancing the CO Pathway

**Scheme 2:**
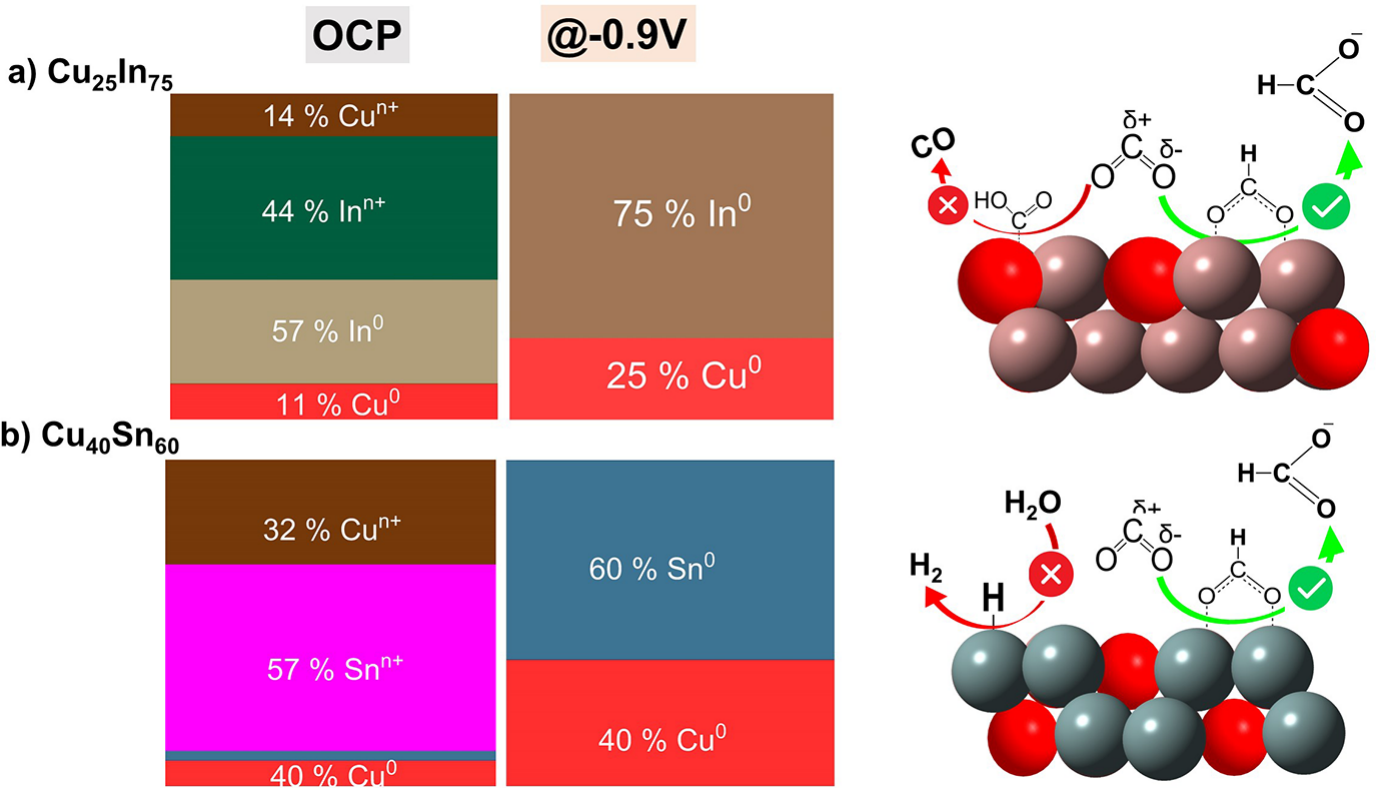
Schematic Diagram Showing the Surface-Active Sites of HCOO^–^-Selective Bimetallic Catalysts and the Role of the Speciation in Enhancing the HCOO^–^ Pathway

A closer look at the surface speciation of these two HCOO^–^-selective bimetallic systems (Cu_25_In_75_ and Cu_40_Sn_60_) reveals the fully metallic nature of all elements at the potential with the best HCOO^–^ production (−0.9 V). Despite their similar speciation and CO_2_ER performance at −0.9 V, they displayed a completely different CO_2_ER behavior at lower potentials (see Figure 3a,b), with Cu_25_In_75_ yielding mainly CO at low potentials (<−0.7 V), while Cu_40_Sn_60_ produces mostly H_2_. These differences in the major products at low potentials may originate from the differences in their surface speciation (Figures 4 and S16). Where the fully metallic surface of Cu_25_In_75_ seems to have a high tendency for CO production with little H_2_ formation, the partially oxidized Sn surface of Cu_40_Sn_60_ favored H_2_ production over CO at low potentials. Schemes 1 and 2 summarize the surface-active species of CO- and HCOO^–^-selective bimetallic catalysts, as evaluated from quasi-in situ XPS, and depict our hypothesis of how they are related to the observed selectivity switch from CO for Cu-rich to HCOO^–^ for Cu-poor bimetallic catalysts. The enhanced CO production at the Cu-rich system can be attributed to the localized partial negative charge on Cu sites due to the charge transfer from Sn/In atoms to Cu. This charge transfer from Sn/In to Cu led to partial positive charge on Sn/In atoms, indicated by the oxidized Sn and In species, and hence stabilization of the *COOH intermediate (CO pathway).^16,23^ Within the resolution of our XPS analysis, we do not observe clear evidence of charge transfer, but rather, both metals are fully reduced on the surface. Thus, Cu-poor catalysts (HCOO^–^-selective) are thought to enhance the HCOO^–^ pathway by inhibiting the H-adsorption and stabilizing the *OCHO intermediate (HCOO^–^ pathway); see in situ SERS below.

Bulk-sensitive XAS measurements were conducted for a set of 16 quasi-in situ samples in order to complement the surface-sensitive XPS measurements.^27^ X-ray absorption near-edge structure (XANES) provides estimates for the oxidation state—particularly the contribution of oxide phases—and the local geometry around the X-ray-absorbing atom. Extended X-ray absorption fine structure (EXAFS) is determined by backscattering of the electron wave created at the X-ray-absorbing atom; it provides bond distances and coordination numbers of the backscattering atoms in the first three–five coordination shells around the X-ray-absorbing atom.

Figure 5a–d displays Cu-, In-, and Sn-XANES of Cu–In and Cu–Sn Cu-rich samples for different potentials. The spectra of metallic foils, typical oxides, and reduced Cu foam for Cu-XANES are given as references. First glance inspection of Cu-XANES immediately hints toward three aspects, in that (1) the Cu–In and Cu–Sn spectra are neither looking like Cu foil Cu_2_O nor pure reduced Cu foam (Figure S17) but are also not just a superposition of those (Figures S18–S20, Tables S1–S7), which is due to the presence of In or Sn atoms within the Cu phase; (2) oxidation states of Cu are close to zero (a quantification was attempted but is largely excluded due to the lack of proper references; see Table S8 for details); and (3) there are only minor responses against applied potentials, which may include the reduction of low amounts of (surface) oxidic species. On the contrary, pronounced changes are obtained for In- and Sn-XANES. Notably, In-XANES of the Cu–In samples can indeed be well approximated as a linear combination^28^ of In foil and In_2_O_3_, and the as-prepared samples basically resemble In_2_O_3_ (Figures 5c and S25), suggesting not only strong oxidic contributions but in addition, the formation of a separate In phase. After application of reducing potentials, the spectra indicate significant reduction and get very close to the In-foil spectra.

The XANES results are supported by the Cu-EXAFS results. Fit models using an In or Sn shell were clearly superior to those using exclusively Cu shells (see Figure 5e and the Supporting Information for details), indicating the formation of Cu–In or Cu–Sn intermetallic phases. The best fitting model and corresponding parameters are shown in Figure S21 and Table S9. The bulk ratios (Figures S28d–S29d) for the Cu-poor samples, however, are not in accordance with XPS, EDX, or ICP–OES, as for all samples—irrespective of element choice (M: In or Sn), suggested ratios (Cu-poor or -rich), or electrochemical treatment (as-prepared or reduced)—Cu/M atomic ratios of 1.5–9 are obtained in EXAFS; that is, 10–40% is In or Sn.

The discrepancy might stem from the fact that Cu-EXAFS only “detects” In or Sn being incorporated into the Cu phase (by means of successful modeling) but is “blind” to a separate M(O_x_) phase, whereas EDX and ICP–OES do not discriminate between phases (while XPS is surface-sensitive) and point toward a thermodynamically preferred mixture with low In or Sn amounts. Combining all techniques, one may argue for a core–shell structure with CuM cores covered by M(O_x_) shells. There is an indication for Sn enrichment in the course of electrochemical operation (from about 10% Sn before to 20–40% after operation), suggesting that parts of the reduced Sn atoms will be embedded into the Cu–Sn (core) structure. Figures 5f and S22 display Cu-EXAFS for all samples in FT representation, showing large peaks for Cu–In samples and systematically lower peaks for the Cu–Sn samples, suggesting a considerable degree of crystallinity in the former and the lack of such in the latter. This is supported by the fact that not only the absolute peak heights are larger for Cu–In but also that the ratio between the fourth peak at about 4.8 Å reduced distance and the first-shell peak at about 2.2 Å is larger for Cu–In than that for Cu–Sn, indicating a long-range order; see Figure 5f. Accordingly, EXAFS models including multiple-scattering shells were superior in describing Cu–In but inferior in describing Cu–Sn. Figures 5f, S21c,and S22c show that the degree of crystallinity increases with increasing amounts of In in Cu–In and with increasing potential due to In enrichment. The latter effect is also partially visible in the Cu–Sn spectra. The first Cu-shell populations (NCu1) support these findings, in that they are highest in average for pure Cu foams (10.1), medium for Cu–In foams (8.7), and lowest for Cu–Sn foams (4.9); see also Table S11. With the exception of Cu-rich In, In- and Sn-shell populations tend to rise after application of reductive potential (suggesting element incorporation), and notably, also, the Cu–In and Cu–Sn distances (*R*_In/Sn_) become shorter by 0.03–0.1 Å.

In summary, XAS reveals evidence for the formation of Cu–In or Cu–Sn alloy/intermetallic phases, containing 10–40% In or Sn. This may indicate the amorphous nature of the noticed Cu–In and Cu–Sn alloys by XAS measurements and explains why XRD did not detect any alloy formation. Unlike the XRD technique, crystalline samples are not necessary for XAS measurements, and hence, XAS can be used to study and detect amorphous materials. There are no indications for the Cu oxide phase in the bulk material. In addition, separate In(O_x_) or Sn(O_x_) phases are always detected in the as-prepared materials. These oxide phases get (partially) reduced, and parts of the In or Sn atoms are likely incorporated into the Cu–In or Cu–Sn alloy during operation at catalytic potentials. The metallic Cu–In phase exhibits a considerable degree of crystallinity, which is not observed for the Cu–Sn phase. Table S13 summarizes the findings of the XAS, XRD, and XPS measurements.

#### 3.2.2 In Situ SERS

**Figure 6:**
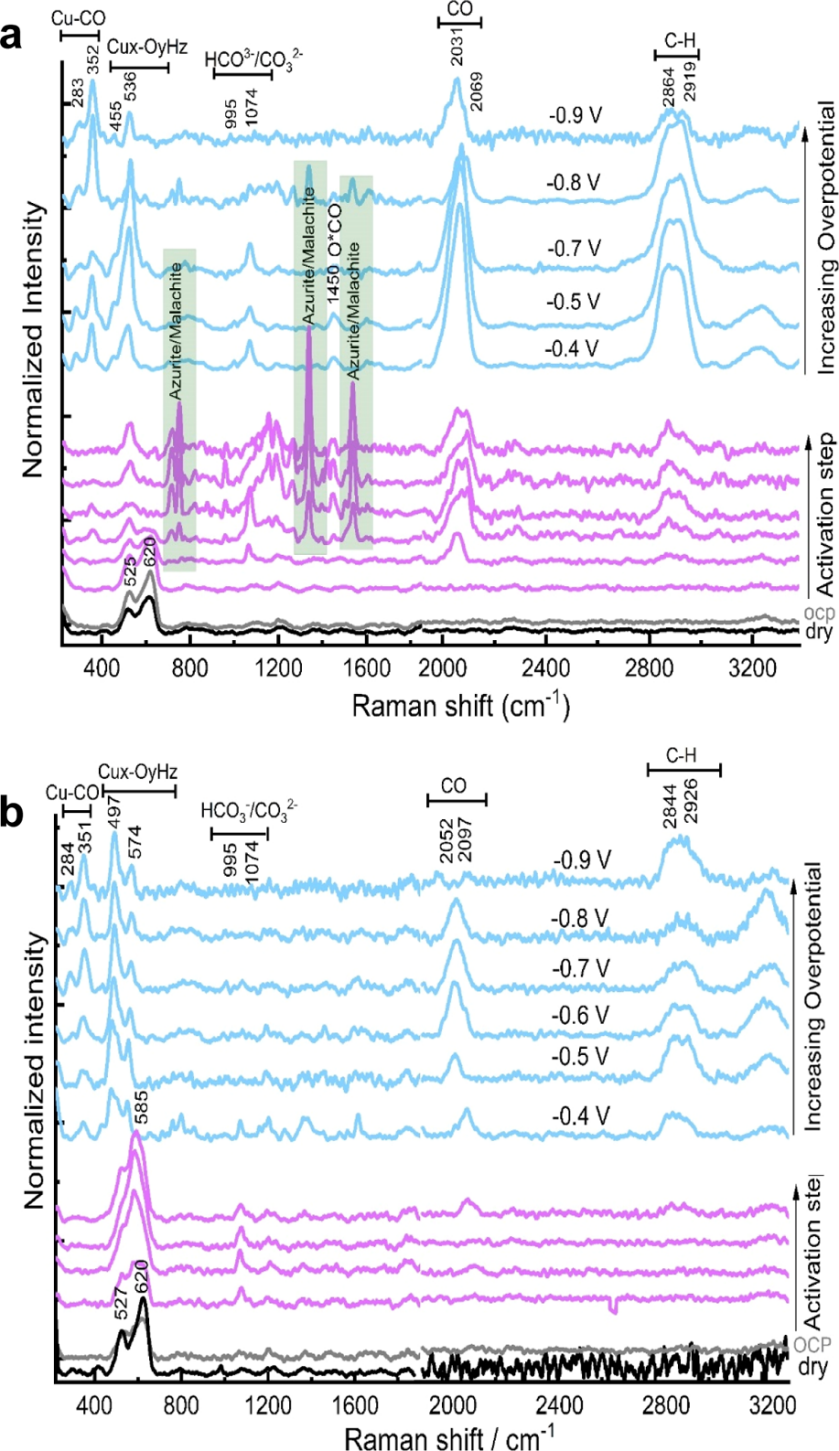
In situ SERS obtained at CO-selective bimetallic foams; namely, Cu_85_In_15_ (a) and Cu_85_Sn_15_ (b), during the activation step (applying −2 mA·cm^–2^ for 15 min) and at various applied potentials (from −0.4 to −0.9 V) in CO_2_-saturated 0.1 M KHCO_3_.

**Figure 7:**
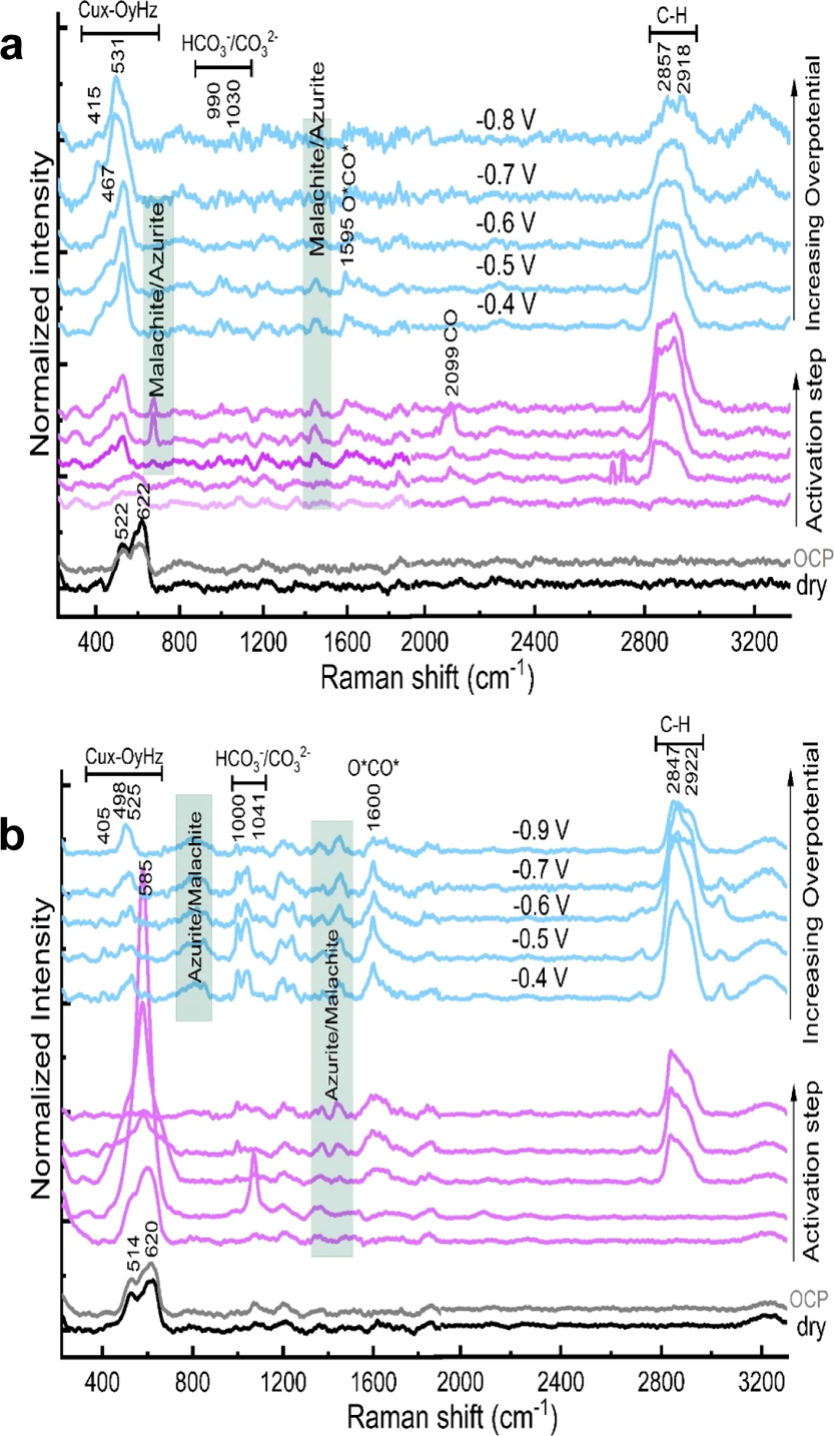
In situ SERS obtained at HCOO^−^-selective bimetallic foams; namely, Cu_25_In_75_ (a) and Cu_40_Sn_60_ (b), during the activation step (applying −2 mA/cm^2^ for 15 min) and at various applied potentials (from −0.4 to −0.9 V) in CO_2_-saturated 0.1 M KHCO_3_.

To obtain further dynamic information about the adsorbed intermediates, local pH, and catalyst surface structure during CO_2_ER, we studied the materials by in situ SERS. The results for the CO- and HCOO^–^-selective bimetallic foams are summarized in Figures 6, 7, and S30–S32. Under open-circuit potential (OCP) and dry conditions, all the investigated catalyst materials including the bimetallic foams showed two strong peaks located at ∼522–527 and ∼619–623 cm^–1^.^29^ We measured the Raman spectra of different Cu oxide/hydroxide standards [e.g., Cu_2_O, CuO, Cu(OH)_2_] for comparison, see Figure S28. Since these two aforementioned peaks do not perfectly match with any of the single oxide/hydroxide standards, we attribute them to a mixture of Cu_2_O and CuO with Cu_2_O as the dominant surface species, which is in good alignment with the obtained XPS results. For in situ measurements (see the Experimental Section for more details), the as-prepared catalyst materials are first pre-activated (reduced) by applying a constant cathodic current of −2 mA·cm^–2^ in a CO_2_-saturated solution of 0.1 M KHCO_3_ until −0.4 V are reached. Then, a constant potential of interest is applied for 2 h to replicate relevant CO_2_ER conditions. Interestingly, Cu–In bimetallic foams exhibited different activation–reduction behavior compared to the Cu–Sn bimetallic foams. The initial Cu_2_O/CuO surface oxides of the as-prepared Cu–In bimetallic foams seem to be dynamically reduced with near-instantaneous formation of a basic copper carbonate phase [metastable malachite-/azurite-like materials, Cu_2_CO_3_(OH)_2_/Cu_3_(CO_3_)_2_(OH)_2_],^30^ indicated by observation of vanishing Cu_2_O/CuO peaks at 525 and 620 cm^–1^ and the immediate appearance of several new peaks assigned to malachite/azurite phases, as shown in Figures 6a and 7a.

On the other hand, the Cu_2_O/CuO peaks (at 514–527 and 620 cm^–1^) of the as-synthesized Cu–Sn bimetallic foams do not fade away during the pre-activation step, but rather, they evolve into a very strong peak at 585 cm^–1^, which completely disappears within 10 min of the pre-activation step, as shown in Figures 6b and 7b.

The pure Cu and bimetallic foams exhibited several peaks between 411 and 5240 cm^–1^ under CO_2_ electrolysis at all investigated potentials. These peaks could be assigned to either a single or a mixture of Cu oxide/(oxy)hydroxide surface species. For example, Cu_85_In_15_ (CO-selective) and Cu_25_In_75_ (HCOO^–^-selective) exhibited a strong peak (at 530–536 cm^–1^) combined with a pre-shoulder peak (at 455–467 cm^–1^) under CO_2_ electrolysis at all studied potentials (between −0.4 and −0.9 V). These two peaks could be attributed to a mixture of Cu(OH)_2_ and basic Cu carbonate [Cu_x_(CO_3_)_y_(OH)_z_, malachite/azurite] phases since their positions match those of some of the obtained peaks for our internally measured Cu(OH)_2_ and malachite standards. Additionally, the pure Cu foam exhibited a strong peak at 498 cm^–1^ with a pre-shoulder peak at 461 cm^–1^, which can be attributed to Cu(OH)_2_ based on alignment with that standard. Moreover, Cu_85_Sn_15_ (CO-selective) and Cu_40_Sn_60_ (HCOO^–^-selective) also showed a strong peak at 597–598 cm^–1^ assigned to Cu(OH)_2_. Cu_40_Sn_60_ showed additional peaks that can be assigned to malachite/azurite, as shown in Figure 7b. We will refer to all the observed peaks between 400 and 630 cm^–1^ as a mixture of copper (oxy)hydroxide and carbonate species since the obtained peaks in this region for our synthesized Cu-based bimetallic foams are broad peaks compared to those of the measured Cu standards (see Figure S30). Indeed, the obtained operando SERS results provide evidence for the persistence of copper oxide surface species and their *in situ* formation and transformation under CO_2_ electrolysis conditions. Important to note is that in the absence of CO_2_, neither of these peaks assigned to surface copper (oxy)hydroxide/carbonate species were observed. This highlights the essential role of the local environment changes developed during CO_2_ electrolysis, such as local pH increase, in the dynamic transformation of Cu_2_O/CuO of the as-prepared catalysts into Cu_x_(CO_3_)_y_(OH)_z_ under CO_2_ electrolysis conditions. That is, surface Cu_2_O/CuO of the as-prepared samples in situ converted into a mixture of Cu hydroxide [Cu(OH)_2_] and basic Cu carbonate (malachite/azurite) during CO_2_ electrolysis. The equilibrium between these two copper surface species is strongly dependent on the developed local environmental changes under CO_2_ electrolysis. It is worth mentioning here that there are no peaks identified for either In or Sn oxides under the applied measurement conditions, attributed to their very low Raman scattering intensity due to the lack of the localized surface resonance.

The bicarbonate/carbonate (HCO_3_^–^/CO_3_^2–^) equilibrium was studied using operando SERS to quantify the induced local alkalization which occurs under CO_2_ electrolysis conditions. The HCO_3_^–^/CO_3_^2–^ equilibrium shifts toward CO_3_^2–^ with the increase in pH,^28^ resulting in a decrease in the intensity of HCO_3_^–^ Raman bands (at 1018 and 1365 cm^–1^) and simultaneous increase in the CO_3_^2–^ band (at 1066 cm^–1^). As shown in Figure S31, the intensity of this CO_3_^2–^ band grew with the application of more negative potentials, especially for pure Cu foam. For instance, the Cu foam local pH shifted from 6.8 at OCP to ∼11 at −0.7 V, while the local pH of CO-selective bimetallic foams rose to 9.0 at the same potential, providing direct evidence of the local alkalization developed under CO_2_ electrolysis. Furthermore, HCOO^–^-selective bimetallic foams also showed a local alkalization under CO_2_ electrolysis, especially at higher applied potentials. Based on these observations, we were curious why these Cu hydroxide/carbonate surface species did not appear in our quasi-in situ glovebox-assisted XPS and XAS analyses. The main difference here is that the Raman spectra are acquired during continuous applied bias. Thus, we investigated the effects of bias removal on these detected Cu hydroxide/carbonate peaks to evaluate the possibility of surface transformation at the open circuit in the electrolyte. We followed the induced changes in the obtained Raman spectra of our synthesized bimetallic foams over time following the bias removal while either keeping them inside the same electrolyte or exposing them to air. As seen in Figure S32, the typical Cu_2_O/CuO mixture peaks were observed along with the complete disappearance of Cu_x_(CO_3_)_y_(OH)_z_ peaks for all investigated catalyst materials after taking them out of the electrolyte even for short time (<5 min). On the other hand, while remaining immersed, these in situ-formed Cu_x_(CO_3_)_y_(OH)_z_ phases formed during CO_2_ electrolysis stay observable for some time (15–25 min) after the bias removal. After that time, the equilibrium between Cu(OH)_2_ and basic copper carbonate (malachite/azurite) shifts toward basic copper carbonate with time, as demonstrated by the appearance of several new peaks which can be assigned to malachite/azurite phases. We therefore conclude that while our quasi-in situ XPS approach does prevent the re-oxidation of catalyst surfaces after bias removal, it could not observe the Cu hydroxide/basic carbonate surface species since the presence of the local alkalization is essential for the stability of these phases. Both of the two CO-selective bimetallic systems (Cu_85_In_15_ and Cu_85_Sn_15_) showed a similar CO selectivity, despite being significantly different from each other in the dominant copper active-surface species. Even the pure copper foam showed a similar surface Cu species to Cu_85_In_15_ (CO-selective). Thus, we do not find any clear relationship between these observed Cu hydroxide/carbonate surface species and the CO_2_ER for either CO or HCOO^–^ of the different investigated catalyst materials.

The presence of adsorbed *CO on the surface of pure Cu and CO-selective bimetallic (Cu_85_In_15_ and Cu_85_Sn_15_) foams is evidenced by the presence of Raman peaks at ∼280–284, 350–360, and 1970–2070 cm^–1^, assigned to the frustrated CO rotational mode (P1), Cu–CO stretching (P2), and intramolecular C≡O stretching, respectively (Figure 6).^31^ P2 and P1 Raman bands usually indicate the interaction between the CO intermediate and Cu surface. The intensity ratio of P2 and P1 Raman peaks (P2/P1) can be used to assess the *CO surface coverage at the solid–liquid interface during CO_2_ electrolysis, with the ratio and *CO coverage scaling together.^31^ As seen in Figure 6, CO-selective bimetallic foams (Cu_85_In_15_ and Cu_85_Sn_15_) exhibited a higher P2/P1 ratio compared to the pure Cu foam (Figures S33 and S34), suggesting a higher *CO surface concentration at the bimetallic foams compared to that at the pure Cu foam, which correlates with higher FEs for CO evolution. On the other hand, neither of the CO-adsorption-related peaks were observed for the HCOO^–^-selective bimetallic foams (Cu_25_In_75_ and Cu_40_Sn_60_), which is expected since they are mainly HCOO^–^ producers (Figure 7). Additionally, both exhibited a strong broad peak at 2840–2950 cm^–1^ assigned to the C–H stretching (mainly HCOO^–^) together with a well-resolved peak at ∼1595–1600 cm^–1^ attributed to the C–O stretching of the HCOO^–^-pathway carboxylate intermediate (O-bound).

Schemes 1 and 2 summarize our observations of the surface-active species of CO- and HCOO^–^-selective bimetallic catalysts (respectively), as evaluated from quasi-in situ XPS and hypothesized general mechanisms of how the speciation relates to the observed selectivity switch from CO for Cu-rich to HCOO^–^ for Cu-poor bimetallic catalysts. The enhanced CO production at the Cu-rich system can be attributed to the localized partial negative charge on Cu sites due to the charge transfer from Sn/In atoms to Cu. This charge transfer from Sn/In to Cu led to partial positive charge on Sn atoms, indicated by the observed oxidized Sn and In species, and hence stabilized the *COOH intermediate (CO pathway).^16,23^

## 4 Conclusions

Composition-tunable Cu–In and Cu–Sn bimetallic foams with a dendritic nanomorphology were synthesized via a simple co-electrodeposition approach. Their compositions were tuned and optimized to achieve high CO_2_ER selectively toward either HCOO^–^ or CO. The selectivity of the prepared bimetallic foams toward CO_2_ER strongly depends on the catalyst composition. The Cu-rich bimetallic foams (85% Cu, Cu_85_In_15_ and Cu_85_Sn_15_) showed a high CO selectivity, while the Cu-poor bimetallic foams (25–40% Cu, Cu_25_In_75_ and Cu_40_Sn_60_) showed a high HCOO^–^ selectivity. Despite both Cu–Sn and Cu–In bimetallic systems showing a similar CO_2_ER dependency on the surface composition (Cu/Sn and Cu/In surface ratios), these bimetallic electrocatalysts showed significant differences in their surface speciation and CO_2_ER-enhancing mechanisms. Specifically, the indium and tin surface speciation is strongly dependent on the composition and the applied potentials. The surface of HCOO^–^-selective bimetallic foams (Cu_40_Sn_60_ and Cu_25_In_75_) exhibited exclusively metallic surface species (Cu^0^, Sn^0^, and In^0^) at potential with the highest HCOO^–^ selectivity (−0.9 V); however, they were found to enhance the HCOO^–^ pathway via different mechanisms. Cu_40_Sn_60_ enhances the HCOO^–^ selectivity at the expense of the parasitic hydrogen evolution reaction, while Cu_25_In_75_ improves HCOO^–^ production at the expense of CO.

At all potentials below −0.9 V, 80% of the surface indium of Cu_25_In_75_ exists as metallic In (In^0^) with low contribution of oxidized indium species, while under the same conditions, the surface speciation of Cu_40_Sn_60_ (HCOO^–^-selective) showed that ∼52% of the surface tin persists as oxidized tin species. This may explain the observed differences in their CO_2_ performance at low potentials, where Cu_40_Sn_60_ mainly produces H_2_, while Cu_25_In_75_ showed a high tendency for CO formation under similar conditions. On the other hand, CO-selective bimetallic foams (Cu-rich), Cu_85_In_15_ and Cu_85_Sn_15_, exhibited a similar CO_2_ER behavior, despite the significant differences of their surface speciation. Cu_85_In_15_ (CO-selective) exhibited the optimal CO selectivity at −0.6 V, while Cu_85_Sn_15_ showed the same CO selectivity (90%) at slightly higher potential (−0.7 V). The surface speciation of Cu_85_In_15_ showed that its surface is mainly composed of metallic Cu and metallic In with low contribution of oxidized In species, while the surface of Cu_85_Sn_15_ was found to be composed of metallic Cu and oxidized Sn species with a very low contribution of metallic Sn.

Despite the quasi-in situ XPS and XAS results indicating the full reduction of Cu at all the applied potentials, operando SERS measurements indicate the formation of a thin surface layer of the Cu(OH)_2_/malachite mixture during CO_2_ electrolysis for all the studied catalyst materials (pure Cu and bimetallic foams). The Cu(OH)_2_/malachite equilibrium depends on the local alkalinity developed under CO_2_ electrolysis conditions. Operando SERS enables us to follow the transformation of the copper surface oxides into metallic copper and then into a mixture of Cu(OH)_2_/malachite during the activation step (applying −2 mA·cm^–2^).

The SERS analysis provides direct evidence for the alkalinity developed under CO_2_ electrolysis, where the local pH for the pure Cu foam and the CO-selective bimetallic foams rises from 7–8 to 10–11 during the first 10 min of CO_2_ electrolysis. Operando SERS of the CO-selective bimetallic foams showed strong peaks for the CO intermediates; besides, both of the CO-selective foams showed higher CO surface coverage compared to the pure Cu foams. On the other hand, the HCOO^–^-selective bimetallic catalysts showed only peaks related to the formate pathway (no peaks for the CO pathway were detected) which agree well with the observed CO_2_ER performance. In summary, the complementary information gained from the various spectroscopic and microscopic techniques in ex-, quasi-in situ, or operando approaches allows us to fully examine the origin of the selectivity switch for the bimetallic Cu–M (M = In or Sn) catalysts from CO to HCOO^–^ upon increasing In or Sn contents. The information gathered from these techniques enabled us to understand the significant differences in the surface speciation and composition between Cu–In and Cu–Sn systems, despite both systems showing similar CO_2_ER performance. Our findings from in situ SERS highlight the importance of the developed local alkalinity in the formation of oxidized surface Cu species, where a mixture of Cu hydroxide and basic Cu carbonate (metastable malachite-like materials) was observed.

## Supporting Information Available

Detailed information of the utilized materials, chemicals, and techniques; synthesis procedures of various Cu–Sn and Cu–In catalysts; additional morphological, structural, and compositional characterization for the studied catalysts including SEM, XRD, XPS, and elemental mapping; details of EXAFS fit models and parameters; and additional electrochemical, in situ Raman, and quasi-in situ XAS data (PDF)
