# Supplementary material for: Comparative Spectroscopic Study Revealing Why the CO2 Electroreduction Selectivity Switches from CO to HCOO– at Cu–Sn- and Cu–In-Based Catalysts
Source: ACS Catal. 2022 Dec 5;12(24):15576–89. doi: 10.1021/acscatal.2c04419 (PMC9793466; doi:10.1021/acscatal.2c04419)
Supplement: Supplementary file 1 — cs2c04419_si_001.pdf [file cs2c04419_si_001.pdf]

A comparative spectroscopic study revealing why the CO_2 electroreduction selectivity switches from CO to $HCOO^-$ at Cu-Sn and Cu-In based catalysts

Gumaa A. El-Nagar [a,e*], Fan Yang [b+], Sasho Stojkovikj [a,c+], Stefan Mebs [b], Siddharth Gupta [a,c], Ibbi Y. Ahmet [d], Holger Dau [c], Matthew T. Mayer [a*]

[a] Young Investigator Group Electrochemical Conversion of CO_2, Helmholtz-Zentrum Berlin für Materialien und Energie GmbH

Hahn-Meitner-Platz 1, Berlin 14109, Germany

[b] Department of Physics, Freie Universität Berlin, Arnimallee 14, Berlin 14195, Germany

[c] Institut für Chemie und Biochemie, Freie Universität Berlin, Arnimallee 22, Berlin D-14195, Germany

[d] Institute for Solar Fuels, Helmholtz-Zentrum Berlin für Materialien und Energie GmbH, Hahn-Meitner-Platz 1, Berlin 14109, Germany

[e] Department of Chemistry, Faculty of Science, Cairo University, Giza 12613, Egypt

---

*Corresponding authors: G. A. El-Nagar (Gumaa.el-nagar@helmholtz-berlin.de) & M. T. Mayer (m.mayer@helmholtz-berlin.de)

+ Equally contributed (shared 2nd authorship)

[e] Permanent address of G. A. El-Nagar

S.1. Experimental details

S.1.1. Materials and chemicals

Cu gauze (mesh), 100 mesh woven from 0.11 mm wire diameter (Alfa Aesar); H_2SO_4 (≥98 wt.% ACS grade purity, Merck); HCl (37 wt.% ACS, EMSUR® purity, Merck); $KHCO_3$ (99.7-100.5 wt.% ACS, EMSUR® purity, Merck); Indium(III)bromide (99.999%, ROT® METIC, CARL-ROTH); Copper(II)sulfate pentahydrate (≥98%, ACS reagent, Sigma-Aldrich); Tin(II)sulfate (≥95%, ACS reagent, Sigma-Aldrich). All chemicals were used as received without any further purification. Milli-Q water (18.2 MΩ.cm) was used to prepare all the used solutions in this study.

S.1.2. Catalysis synthesis

Cu-In and Cu-Sn bimetallic foams were electrodeposited at top of a pre-cleaned copper mesh (100-mesh woven, 0.11 mm wire-diameter) using dynamic hydrogen bubbling template (DHBT) technique[1-3] in a two-electrode setup. Bimetallic foams were electrodeposited atop Cu mesh substrate (with rough surface) to improve the mechanical stability of the electrodeposited Cu-based foams. Prior to the electrodeposition, the copper mesh substrate was chemically polished via sonication in 0.5 M HCl to remove the native surface oxide layer and the contamination. The chemically polished copper mesh was next cleaned via sonicated in milli-Q water and absolute ethanol for 10 minutes to remove the HCl impurities, followed by drying step in air. Then, the pre-cleaned copper mesh was masked with PTFE tape to fix the exposed geometric surface area of the copper mesh to 0.4 cm^2. Two Cu foils with total area of 40 cm^2 were used as counter to ensure homogenous electrodeposition of bimetallic foams on both sides of the used copper mesh substrate, as shown in **Scheme S1**. The distance between the anode and cathode kept constant (1 cm).

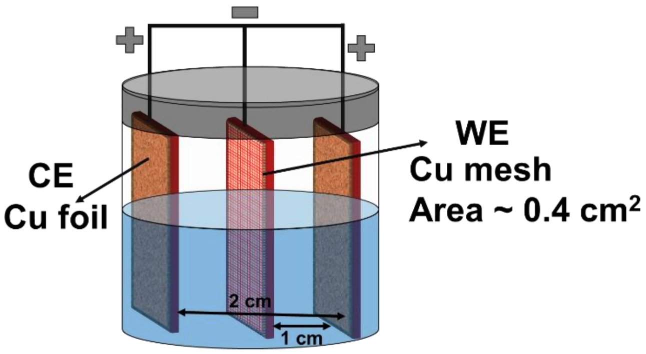

Scheme S1. Schematic diagram of the used two-electrode setup for fabrication various bimetallic foams.

<u>Synthesis of Cu-Sn bimetallic foams:</u> Cu-Sn bimetallic foams were electrodeposited atop of Cu mesh substrate from 30 ml 1.5 M H_2SO_4 solution containing 0.2M $CuSO_4/SnSO_4$ with different Cu/Sn concentration ratios to tune the composition of the electrodeposited Cu-Sn foams. For example, $Cu_{85}Sn_{15}$ foam (CO-selective) was electrodeposited from 30 ml 1.5 M H_2SO_4 containing 0.18 M $CuSO_4$ and 0.02 M SnO_4 (Cu:Sn, 9:1), while $Cu_{40}Sn_{60}$ foam (HCOO-selective) was created from 0.067 M $CuSO_4$ and 0.133 M SnO_4 (Cu:Sn, 1:2) mixed

solution. The electrodeposition was carried via applying a constant current of 1A/cm^2 for 15 s using SP-300 Biologic potentiostat with 4A booster.

Synthesis of Cu-In bimetallic foams: Cu-In bimetallic foams were created in a similar way to Cu-Sn foams. Cu-In bimetallic foams were electrodeposited from 40 ml 1.5 M H_2SO_4 containing 0.2 M $CuSO_4$/$InBr_3$ with various Cu/In ratios to fabricate Cu-In dendrites with various Cu/In ratios. For instance, $Cu_{85}In_{15}$ foam (CO-selective) was tailored from 1.5 M H_2SO_4 electrolyte containing 0.13 M $CuSO_4$ and 0.07 M $InBr_3$ (Cu:In, ~2:1), while $Cu_{25}In_{75}$ foam (HCOO-selective) was fabricated from 0.025 M $CuSO_4$ and 0.175 M $InBr_3$ (Cu:In, 1:7) mixed electrolyte. Galvanostatic mode was used to create Cu-In bimetallic foams via applying current of 1.0 A/cm^2 for 15 s using SP-300 Biologic potentiostat with 4A booster.

For comparison, pure In and Sn catalysts deposited on Cu mesh were also prepared from 1.5 M H_2SO_4 containing 0.2 M $InBr_3$ and 0.2 M $SnSO_4$, respectively, via applying 1 A/cm^2 for 15 s using the same two-electrode setup. It is worth to mention here that two carbon-felt pieces (6 mm thickness) with a total geometric surface area of 40 cm^2 served as counter electrodes instead of the Cu foils to create pure In and Sn catalysts. Additionally, pure Cu foam was prepared in 1.5 M H_2SO_4 containing 0.2 M $CuSO_4$ via applying 1 A/cm^2 for 15 s using Cu foils as counter electrode. All the electrodeposited catalyst materials were carefully rinsed with milli-Q water to clean their surfaces and interior pores from the residuals of the deposition baths.

S.1.3. Material characterization

The morphology, bulk and surface compositions, structure and elemental distribution of the as-prepared bimetallic foams were examined using variety of characterization techniques including Scanning electron microscopy (SEM), Transmission electron microscopy (TEM), Grazing incidence X-ray diffraction (GI-XRD), X-ray photoelectron spectroscopy (XPS), X-ray absorption spectroscopy (XAS), Energy dispersive X-ray (EDX) and inductively coupled plasma-optical emission spectroscopy (ICP-OES). More details about sample preparation and measuring conditions can be found in our recent published articles, where same characterization methods were used[4-5].

SEM images and EDX analysis. The morphology, elemental mapping and bulk composition of the synthesized catalyst materials were examined using LEO 1530 Gemini field emission SEM system equipped with a ThermoFisher Ultradry EDX detector. The SEM images were collected at 4 kV acceleration voltage using a standard aperture size of 30 µm and immersion Lens (in lens) secondary electron detector. Elemental mapping (EDX analysis) was acquired at 15 kV acceleration voltage using aperture size of 60 µm. **Cross sections** SEM images were prepared via focused ion beam milling technique, using a Zeiss Crossbeam 340 KMAT dual beam instrument with Ga ion source, to determine the thicknesses of the electrodeposited foams. The milling cut was directly performed without any protection layer using an acceleration voltage of 30 kV and two different polishing currents (80 nA for rough cut and 1.5 nA for fine polishing).

Grazing incidence X-ray diffraction (GI-XRD). GI-XRD was measured on a PANalytical X'Pert Pro MPD with Cu Kα X-ray source (λ = 1.5418 Å) and xenon scintillation counter detector with parallel plate collimator. The diffractograms were collected using a grazing incidence angle of 1°, step size of 0.05° and 5 s acquisition time.

Inductively coupled plasma-optical emission spectroscopy (ICP-OES). The chemical composition of the bimetallic foams with the best CO and HCOO selectivity was determined using ICP-OES via an iCAP 7400 Duo MFC ICP-OES analyzer (Thermo Scientific) in axial Ar plasma mode.

X-ray photoelectron spectroscopy (XPS). The surface composition and speciation were studied through SPECS PHOIBOS 100 analyzer using Al Kα X-ray excitation source (hv~1486.74 eV). The XPS spectra were acquired using energy step of 0.05 eV, dwell time of 0.1 and energy pass of 10 eV with 90 kV and 2200 kV bias and detector voltage, respectively.

S.1.4 Electrochemical measurements (CO_2 electroreduction testing)

All electrochemical CO_2ER experiments presented in this study were performed using a BioLogic SP-200 potentiostat in H-type two-compartment electrochemical cell separated by a previously activated a cation exchange membrane (Nafion N-115, 0.125 mm thickness, Alfa Aesar)), in CO_2 saturated 0.1 M $KHCO_3$ solution (pH 6.8) under constant CO_2 flow of 20 mL min^{-1}. A Vögtlin instruments mass-flow controller (Red-Y GSC-A9TA-BB21) was used to control the flow rate of CO_2. Pt mesh and Ag/AgCl electrode (filled with 3 M KCl, PalmSens) were served as a counter and reference electrodes, respectively. All applied potentials in this work are converted into the reversible hydrogen electrode (RHE) scale, considering the pH of CO_2-saturated 0.1 M $KHCO_3$ is 6.8.

The electrochemical protocol used during the CO_2ER performance measurements consists of:

i) Applying open circuit potential (OCP) for 10 minutes, followed by resistance measurements (ZIR)
ii) Then, chronopotentiometry (CP) pre-activation step, in which a constant cathodic current of -2 mA cm^{-2} was applied to reach threshold potential of -0.4 V RHE. This step normally lasts for around 15 minutes.
iii) After this pre-activation step, CO_2 electrolysis measurements were carried out by applying constant potential of interest (chronoamperometry) for ~2 hours, typically between -0.5 to -0.9 V RHE

The composition of the gaseous products in the cathode gas outlet stream was analyzed using in-line gas chromatography (Thermo Scientific Model TRACE 1310 gas chromatograph) every 10 minutes. This GC was equipped with a Pulse Discharge Detector (PDD) for H_2 and CO detection, and flame ionization detector (FID) for hydrocarbons quantification (more details can be found in our recent published articles[4-5]. The faradaic efficiency of the gaseous products at a certain applied potential was estimated according to equation 1, where x_i is the concentration of gas i determined by gas chromatography, Q_m is the molar flowrate of CO_2 flowed into the cell (20 ml min^{-1}), z the number of electrons required to produce 1 mol of gas x, and I_{total} is the average recorded current in time interval 2 minutes prior to the GC injection, and F is the Faraday constant

FE of gaseous product (%) = $[(z \cdot F \cdot x_i \cdot Q_m) / I_{total}] \cdot 100$ (Equation 1)

The quantification of non-volatile liquid CO_2RR products (formate and acetate) was conducted using Ultra-High-Performance liquid chromatograph from Thermos Scientific (Model HPLC+ UltiMate 3000 series) with UV variable wavelength (UltiMate 3000, Dionex) and refraction index (RefractoMax 520, ERC) detectors, and a HyperREZ XP H+ column, and a mobile phase of 5 mM H_2SO_4(aq). The quantification of volatile liquid CO_2RR products (including methanol, ethanol and propanol) was performed using gas chromatograph (GC, Thermo Scientific, model Trace 1310) via heated headspace autosampler together with FID and pulse discharge (PDD)

detectors. The percent faradaic efficiency (FE) of formate (HCOO-) was calculated according to equation 2, where z is the number of electrons (z = 2), C_{HCOO} is the measured $HCOO^-$ concentration in the catholyte, F is the Faraday constant (96485.33 $C \cdot mol^{-1}$), $V_{catholyte}$ is the volume of catholyte (25 cm^3), q is the amount of passed charge.

$$\text{FE of HCOO- (\%)} = [(z \cdot C_{HCOO-} \cdot F \cdot V_{catholyte}) / q] \cdot 100 \qquad \text{(Equation 2)}$$

S.2. Additional characterization and experimental data

SEM images of the various as-prepared Cu-Sn and Cu-In foams.

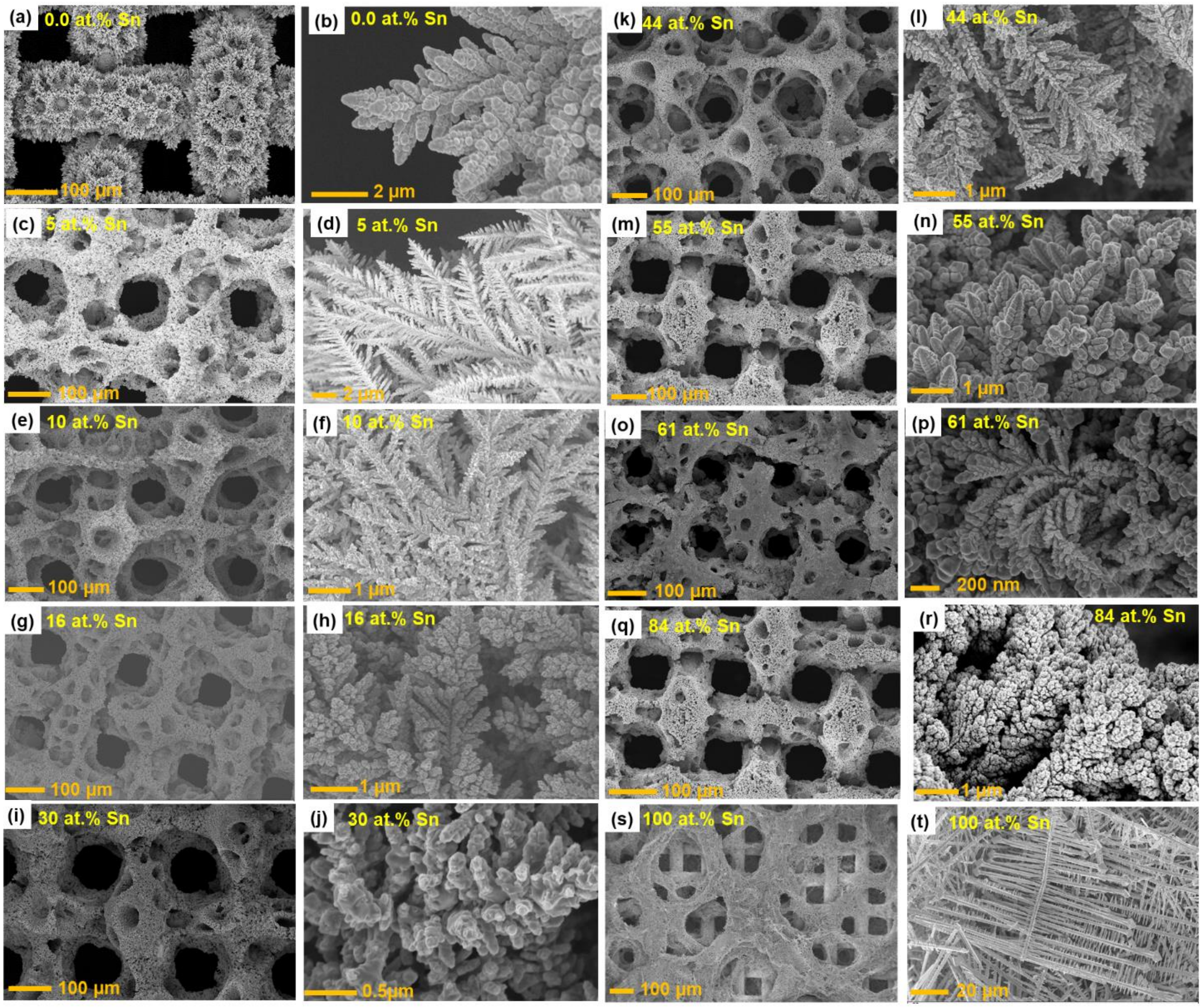

Figure S1. SEM images of as-prepared pure Cu foam and Cu-Sn bimetallic foams with various Sn contents. (a,b) pure Cu foam (100 at.% Cu) and (c-t) Cu-Sn bimetallic foams with various at.% Sn: (c,d) 5.0 at.%, (e,f) 10 at.% , (g,h) 16 at.%, (I,j) 30 at.%, (k,l) 44 at.%, (m,n) 55 at.% , (o,p) 61 at.%, (q,r) 84 at.% and (s,t) 100 at.%. The provided Sn at.% was estimated from EDX.

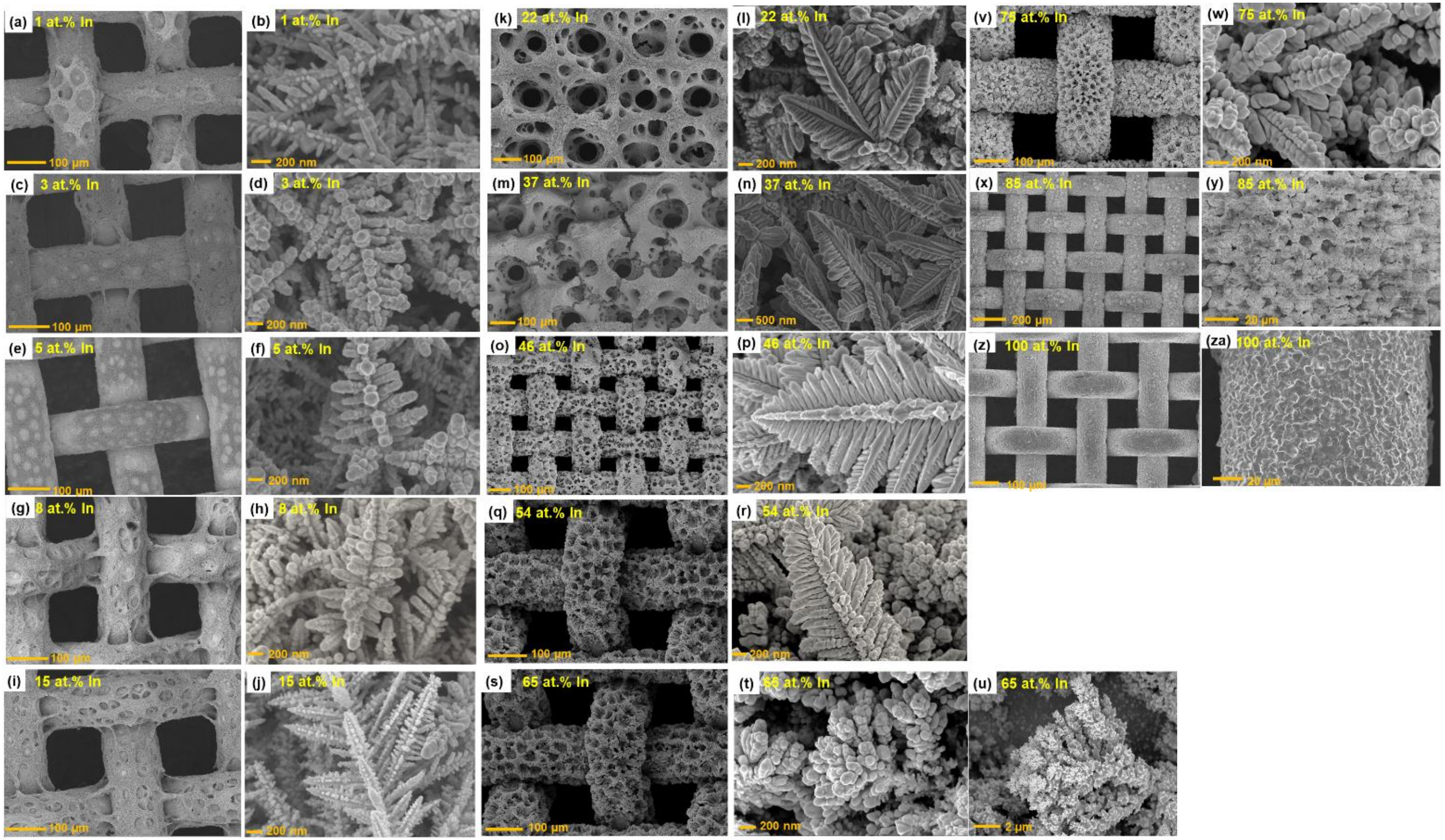

Figure S2. SEM images of the as-synthesized Cu-In bimetallic foams with various In wt.%; (a,b) 1.0 at.%, (c,d) 3.0 at.%, (e,f) 5 at.%, (g,h) 8.0 at.%, (i,j) 15 at.%, (k,l) 22 at.%, (m,n) 37 at.% , (o,p) 46 at.%, (q,r) 54 at.%, (s-u) 65 at.%, (v,w) 75 at.%, (x,y) 85 at.%, and (z,za) 100 at.% . The provided Sn at.% are based on EDX analysis.

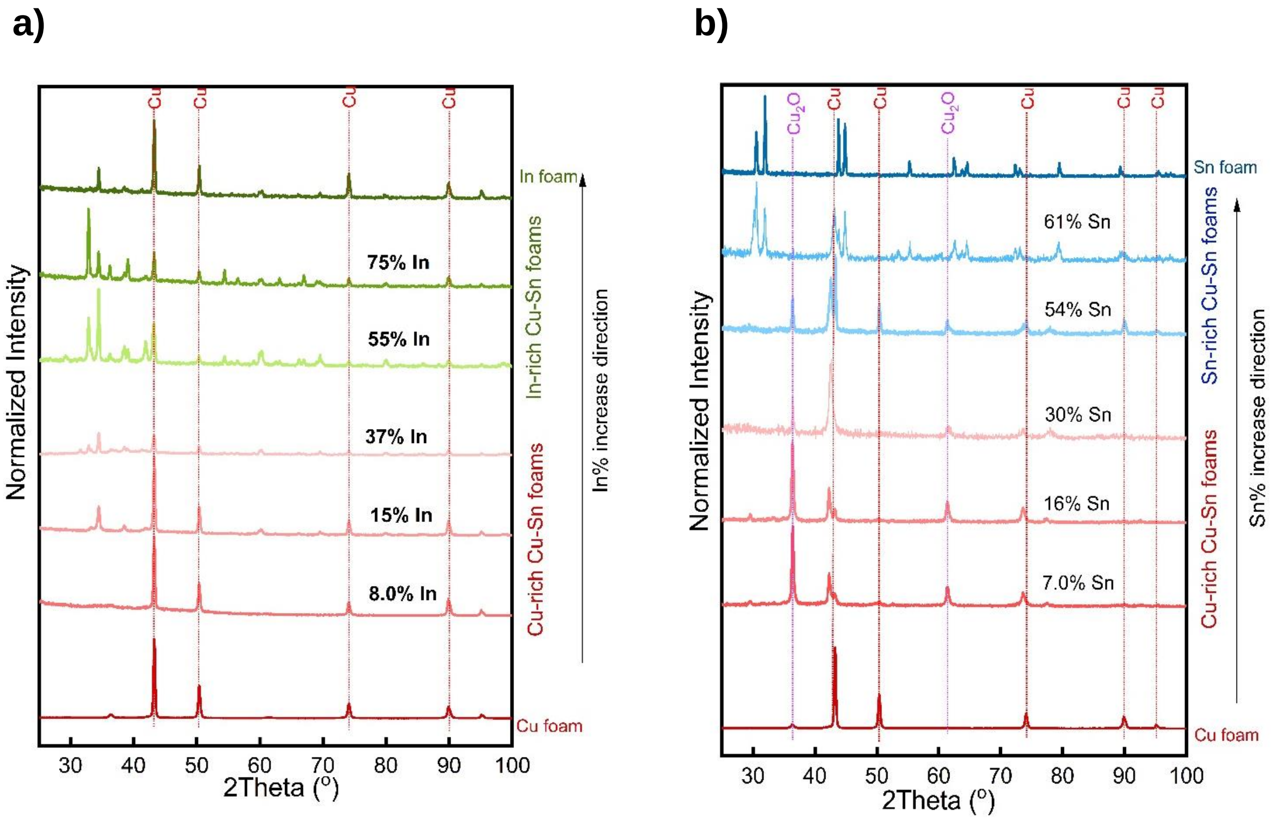

Figure S3. Representative XRD patterns of some selected as-prepared Cu-In (a) and Cu-Sn (b) bimetallic foams with various Sn and In contents, respectively, deposited onto Cu mesh substrates. The labeled In and Sn contents are obtained from their respective EDX analysis. The dotted lines indicate peaks attributable to the Cu phases mentioned in the legend, at peak positions derived from reference patterns for each material. The unassigned peaks are attributed to bulk metallic In and Sn phases of Cu-In and Cu-Sn foams, respectively.

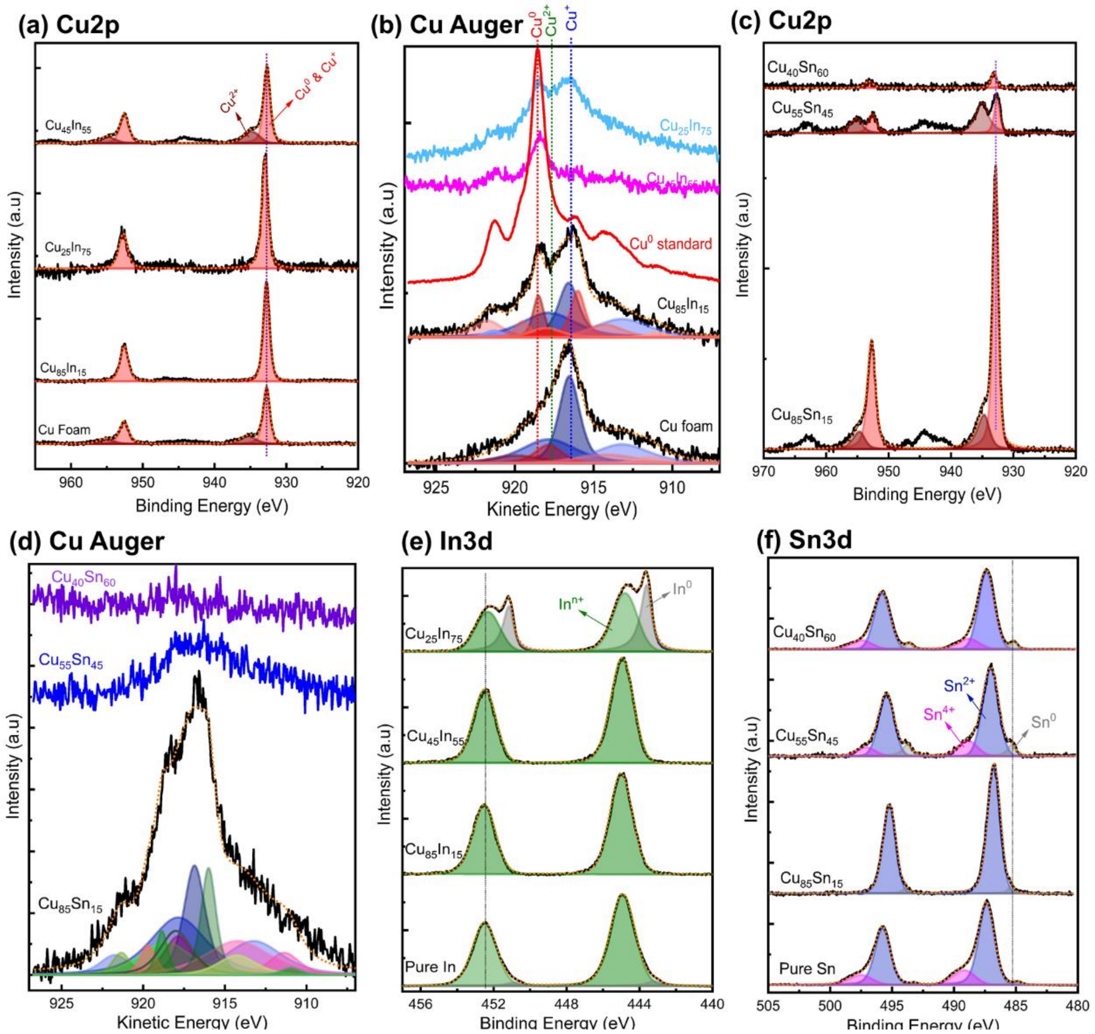

Figure S4. XPS measurements for the as-prepared pure (Cu, In and Sn) and bimetallic (Cu$_{85}$In$_{15}$, Cu$_{85}$Sn$_{15}$, Cu$_{40}$Sn$_{60}$, Cu$_{25}$In$_{75}$, Cu$_{45}$In$_{55}$ and Cu$_{55}$Sn$_{45}$) foams. High-resolution XPS spectra of (a) Cu 2p of pure Cu and Cu-In foams and (b) their respective Cu auger regions. (c) Cu 2p of Cu-Sn bimetallic foams and (d) their corresponding Cu auger regions. (e) In3d of pure In and Cu-In bimetallic foams and (f) Sn 3d of pure Sn and Cu-Sn bimetallic foams.

As clearly seen in this figure, the surface of all the as-synthesized catalyst materials exhibited mainly oxidized surface species, despite of their XRD patterns showed their bulk metallic nature (**Figure 2**, **main manuscript**). Where Cu-Sn bimetallic foams: Cu$_{85}$Sn$_{15}$ (15 at. Sn%), Cu$_{40}$Sn$_{60}$ (60 at. Sn%) and Cu$_{55}$Sn$_{45}$ (45 at. Sn%), exhibited mainly oxidized Sn surface species with very small contribution from metallic Sn, as clearly seen in **Figure S3f**. Cu-In bimetallic foams: Cu$_{85}$In$_{15}$ (15 at. In%), Cu$_{25}$In$_{75}$ (75 at. In%) and Cu$_{45}$In$_{55}$ (55 at. In%), showed a similar behavior, where all of them showed fully oxidized In surface species except Cu$_{25}$In$_{75}$ exhibited mixture of metallic and oxidized In surface species (**Figure S3e**). Closer look at Cu2p spectra and Cu auger regions revealed that surface of the all as-synthesized catalyst materials is predominant by the oxidized Cu surface species. For instance, the pure Cu and

$Cu_{85}Sn_{15}$ foams showed mixture of Cu^{+} (is the dominant species) and Cu^{2+}, while $Cu_{85}In_{15}$ foam exhibited a mixture of metallic and Cu^{+} surface species. This will be discussed in more details together with the obtained results from quasi in-situ XPS measurements in section 3.3 of the main manuscript

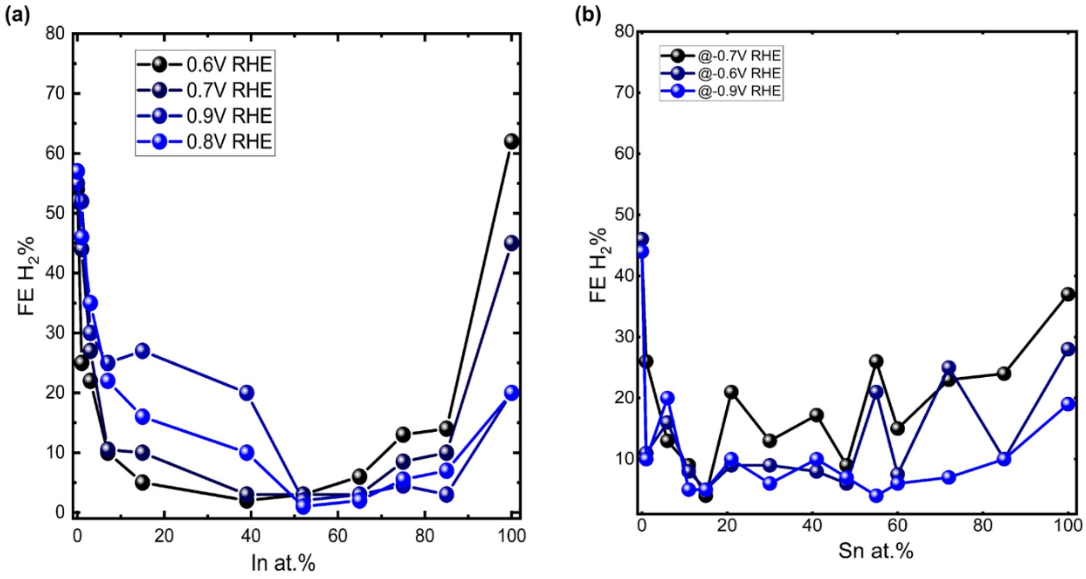

Figure S5. The variation of faradaic efficiency of H_2 (FE H_2%) as a function of In and Sn atom percent composition at different potentials.

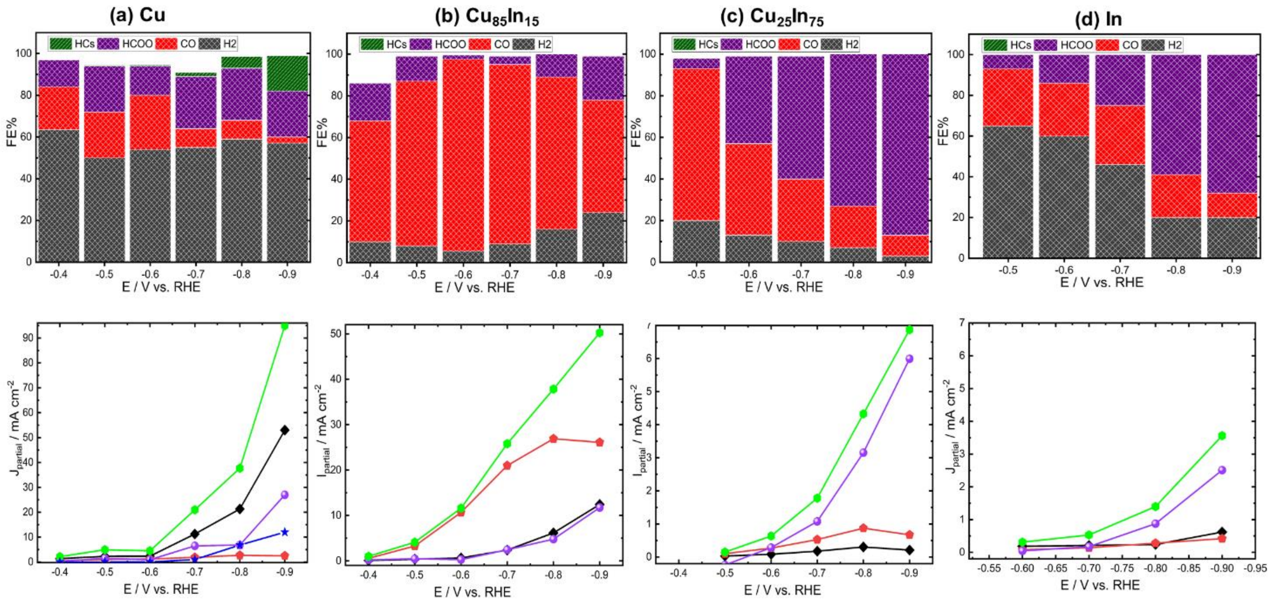

Figure S6. The distribution of faradaic efficiency and the partial current density of the various major products obtained at pure Cu foam (a), $Cu_{85}In_{15}$ (b), $Cu_{25}In_{75}$ (c) and pure In (d) at various applied potentials. The green curves in the partial current density graphs are presenting the total CO2ER current.

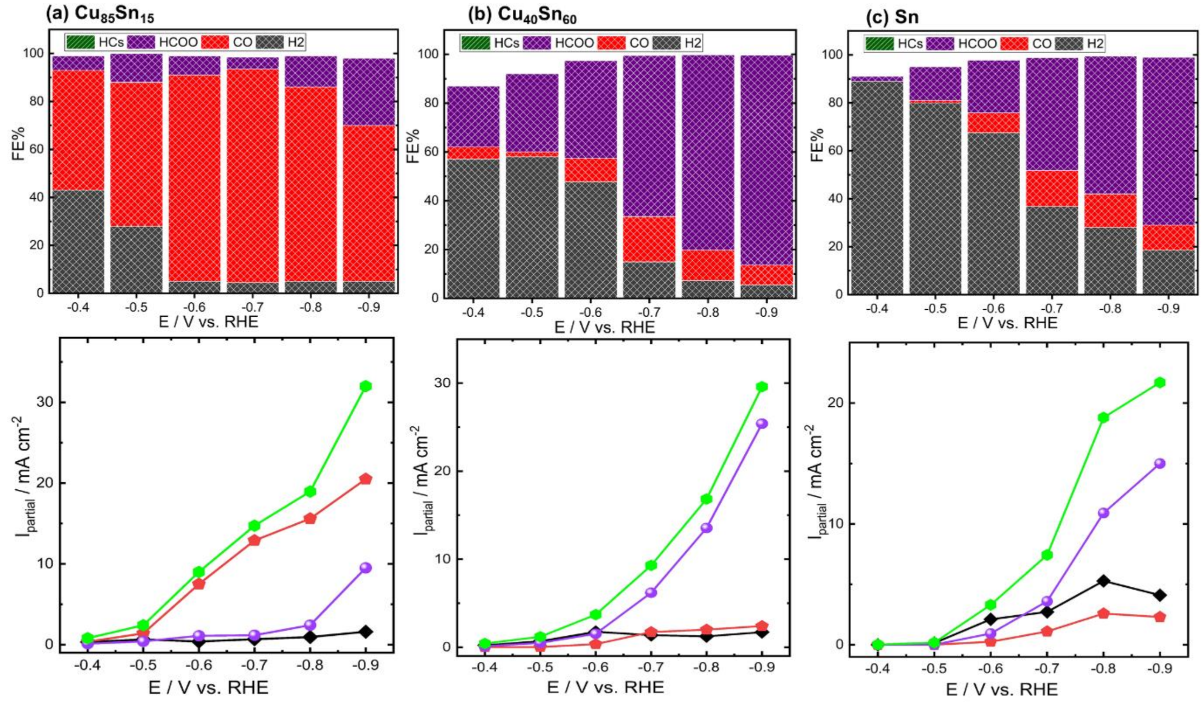

Figure S7. The distribution of faradaic efficiency of the various major products and their respective partial current density of $Cu_{85}Sn_{15}$ (a), $Cu_{40}In_{60}$ (b) and pure Sn (c) at various applied potentials.

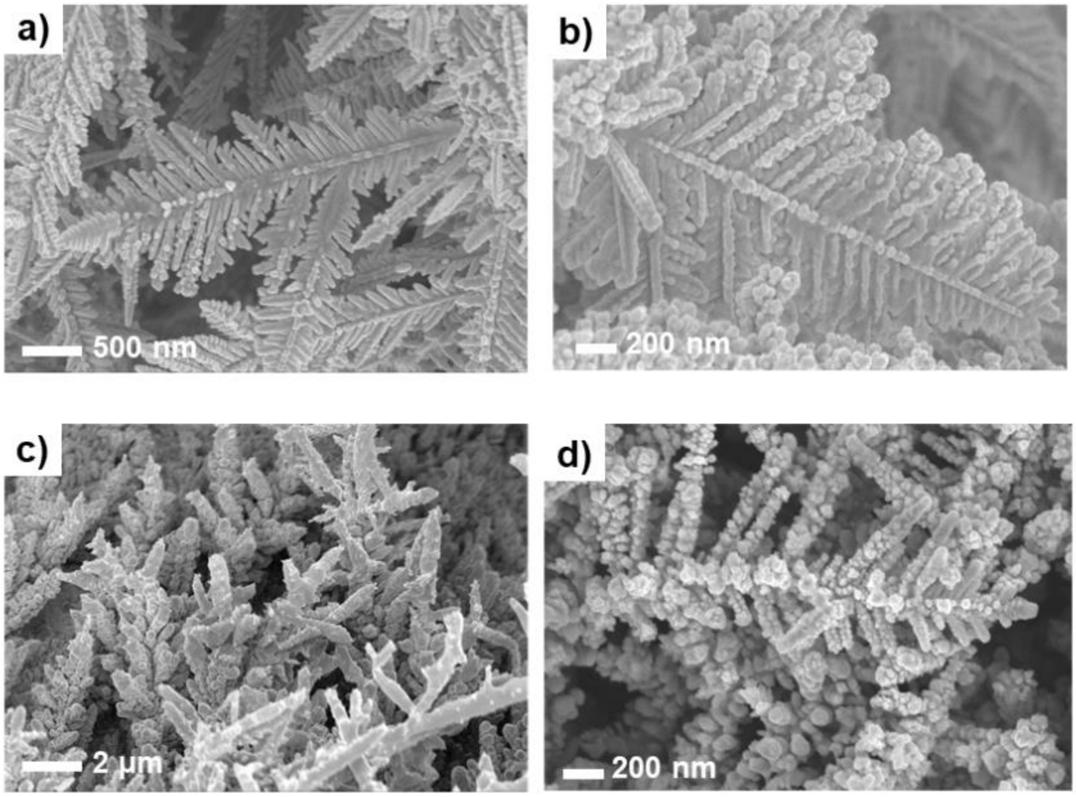

Figure S8. Post analysis SEM images of $Cu_{85}In_{15}$ (a), $Cu_{25}In_{75}$ (b), $Cu_{85}Sn_{15}$ (c) and $Cu_{40}Sn_{60}$ bimetallic foams.

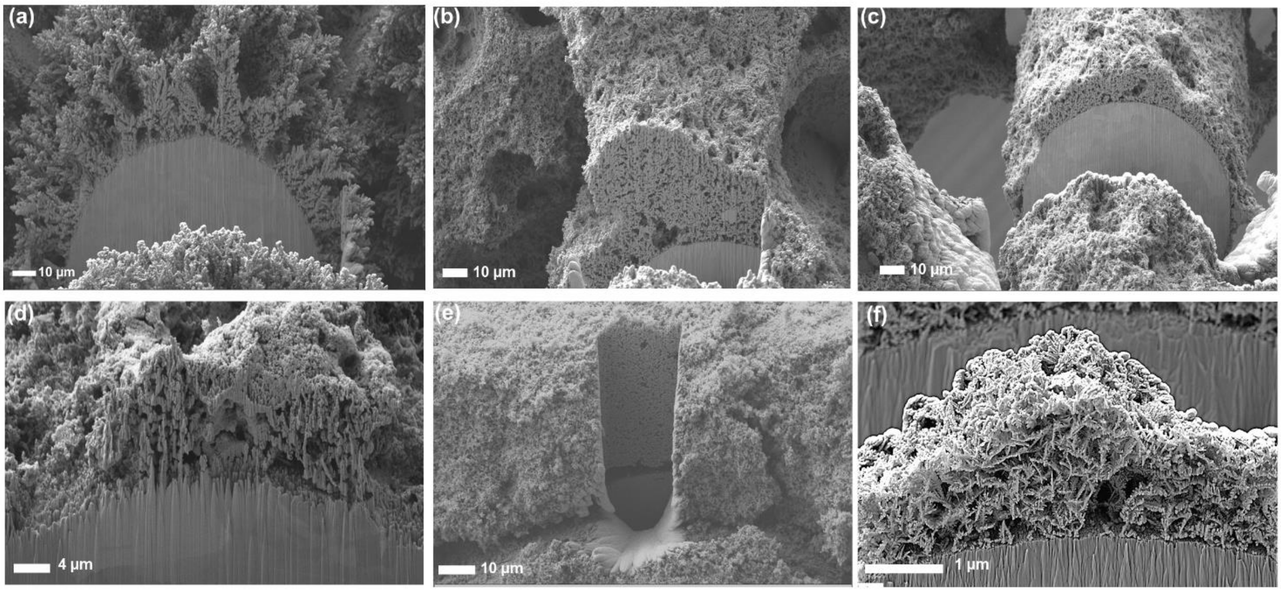

Figure S9. Cross section SEM prepared by using FIB for (a) Cu foam, (b) $Cu_{85}In_{15}$, (c) $Cu_{44}In_{65}$, (d) $Cu_{25}In_{75}$, (e) $Cu_{85}Sn_{15}$ and (f) $Cu_{40}Sn_{60}$.

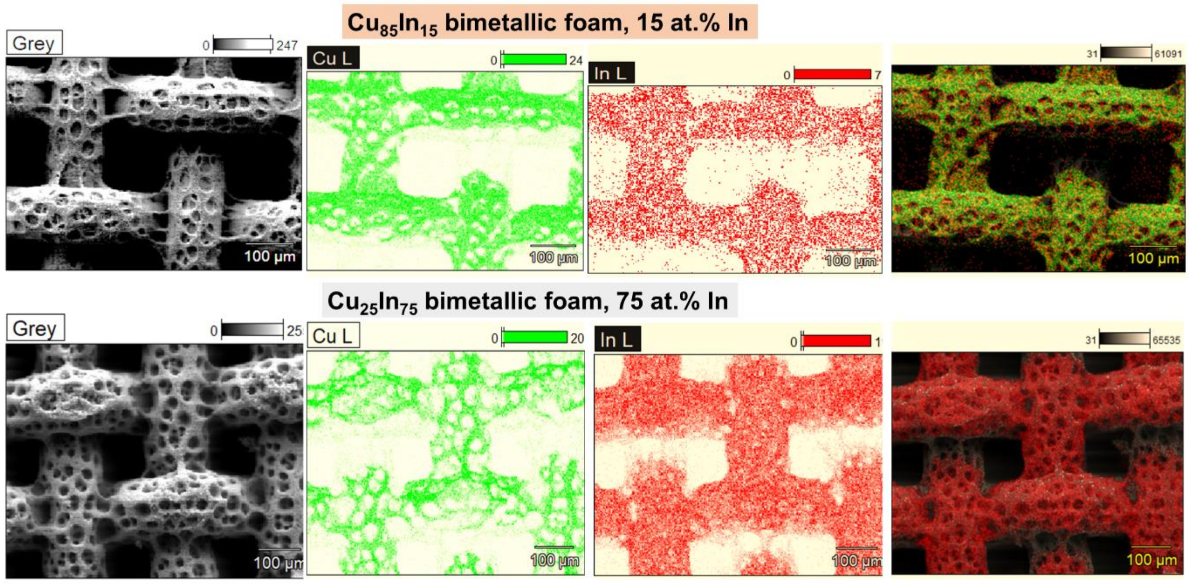

Figure S10. Elemental mapping by EDX of $Cu_{85}In_{15}$ and $Cu_{25}In_{75}$ bimetallic foams.

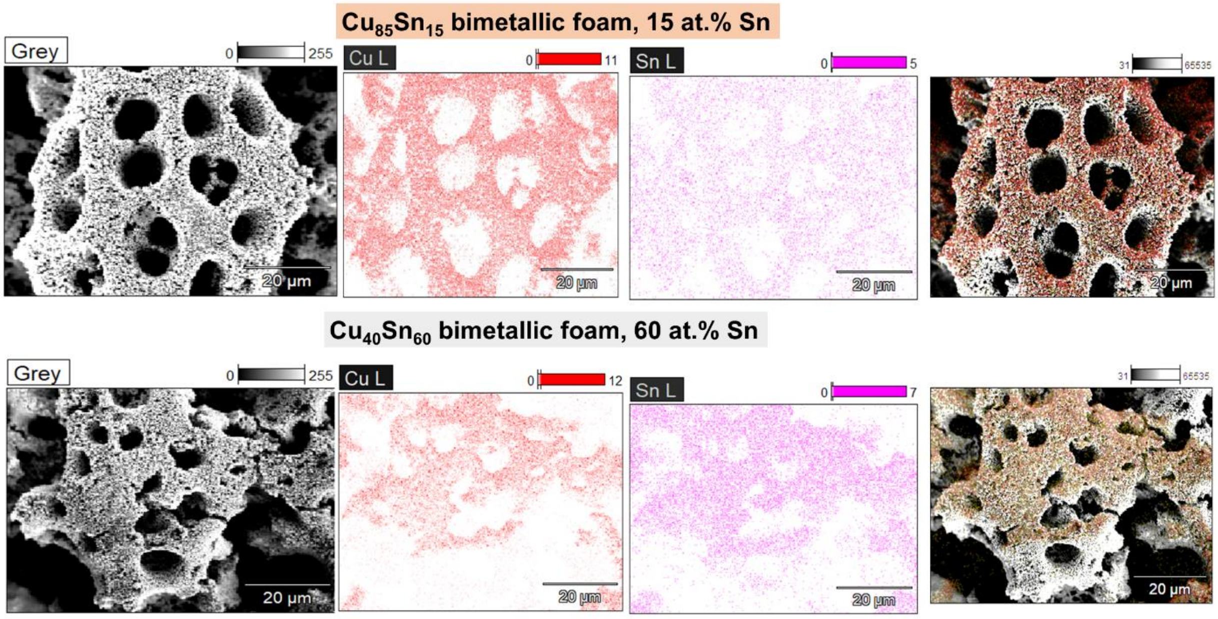

Figure S11. Elemental mapping by EDX of $Cu_{85}Sn_{15}$ and $Cu_{40}Sn_{75}$ bimetallic foams.

Estimation of the relative surface roughness factor (RF). The roughness factor of the various as-prepared Cu-In and Cu-Sn bimetallic foams were estimated from their respective double layer capacitance via measuring cyclic voltammetry (CV) in a non-faradaic region at different scan rates (10-250 mV/s), more details can be found in our recent publication[4]. Briefly, the double layer capacitive (C_{dl}) of the as-prepared bimetallic foams was determined from the below equation (eq. 1):

$$\Delta \mathbf{J}_{\mathbf{capacitive}} = (\mathbf{J}_{\mathbf{anodic}} - \mathbf{J}_{\mathbf{cathodic}}) = \mathbf{C}_{\mathbf{dl}} * \mathbf{v} + \mathbf{a}_{\mathbf{intercept}} \qquad \mathbf{eq.\,1}$$

Where ΔJ is the capacitive current and it is defined as the difference between the anodic (J_{anodic}) and cathodic ($J_{cathodic}$) current density, and v is the scan rate (mV/s) and C_{dl} is the double layer capacitance. The C_{dl} can be estimated from the slope of the linear relation between capacitive current (ΔJ) and scan rate. Relative roughness factor (RF) of the bimetallic systems was estimated by the ratio between the C_{dl} of the bimetallic foam and the C_{dl} of the pristine Cu mesh substrate (obtained data summarized in **Table 1**):

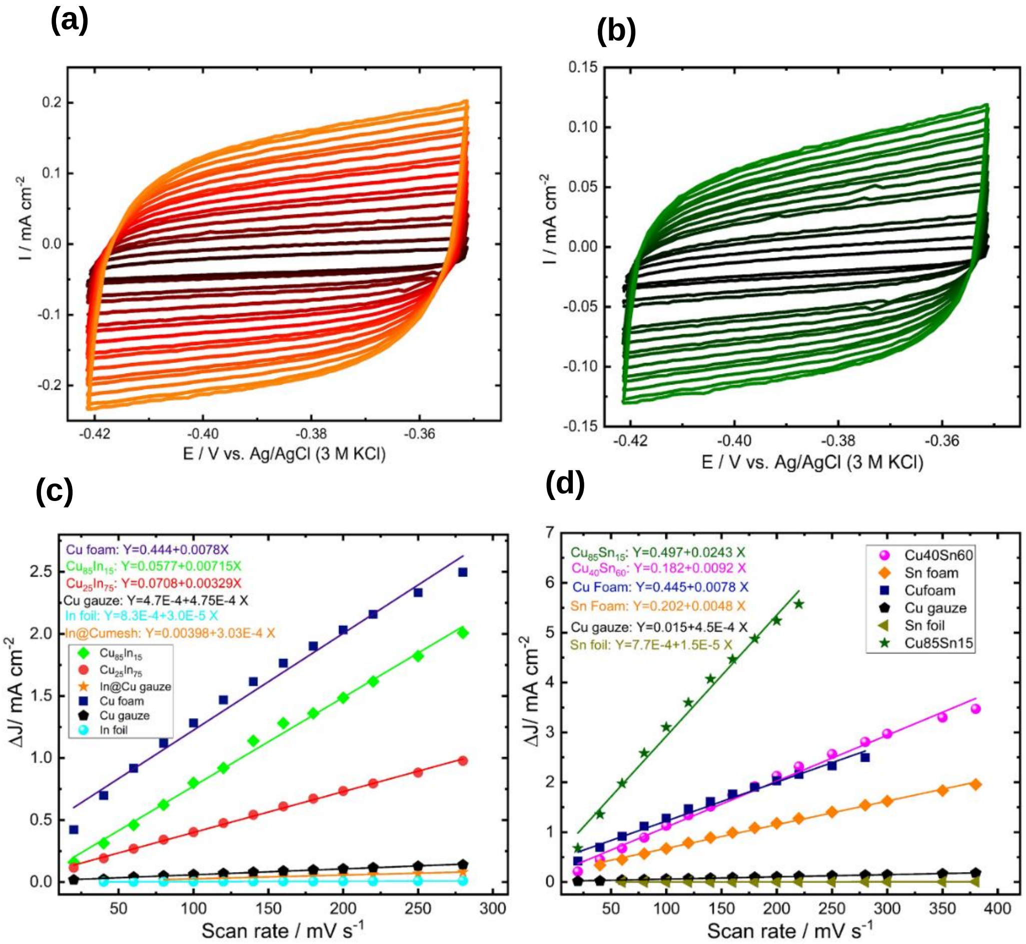

Figure S12. Two examples for CVs measured in the non-faradaic current region of potential recorded in CO_2-saturated 0.1 M $KHCO_3$ electrolyte at Cu foam (a) and $Cu_{85}Sn_{15}$ bimetallic foam (b) at different scan rates (10-250 mV/s). (c,d) The variation of capacitive current of the various as-prepared catalyst materials as function of scan rate: (c) Variation of Cu foam, $Cu_{85}In_{15}$, $Cu_{25}In_{75}$, Cu mesh, In foil and In decorated Cu

mesh capacitive current with scan rate. (d) Variation of capacitive current of Cu foam, $Cu_{40}Sn_{60}$, $Cu_{85}Sn_{15}$, Sn decorated Cu mesh, Cu mesh and Sn foil capacitive current with scan rate.

Quasi in-situ XPS measurements. XPS measurements were performed in SPECS PHOIBOS 100 analyzer using Al Kα X-ray excitation source (hv~1486.74 eV). The high-resolution XPS spectra were acquired using energy step of 0.05 eV, dwell time of 0.1 and energy pass of 10 eV with 90 kV and 2200 kV bias and detector voltage, respectively. The CO_2 electrolysis were examined under inert atmosphere inside a glovebox equipped with oxygen (O_2<1ppm) and water (H_2O<5 ppm) sensors using the same H-cell and conditions, which are used to measure the CO2R performance of the different synthesized catalyst materials as described in detail under **S.1.4**. Next, the samples were loaded onto the XPS sample holder inside the glovebox and transferred from the glovebox into XPS analysis chamber under N_2 using a specially designed transfer arm, which avoids any air exposure and surface re-oxidation. The obtained spectra were fitted using Fityk free software via subtracting of a Shirley background. The spectra were calibrated with respect to the measured adventitious carbon (C1s) peak at 285 eV. Cu LMM Auger region was collected for the various prepared catalyst materials and fitted using the reported values for various copper species (Cu_2O, CuO and Cu) by Biesinger [6], since it was difficult to differentiate between metallic Cu and Cu_2O species using the Cu2p spectra alone. We also prepared a clean metallic copper reference by Ar sputtering the surface of Cu foil to validate our fitting parameters.

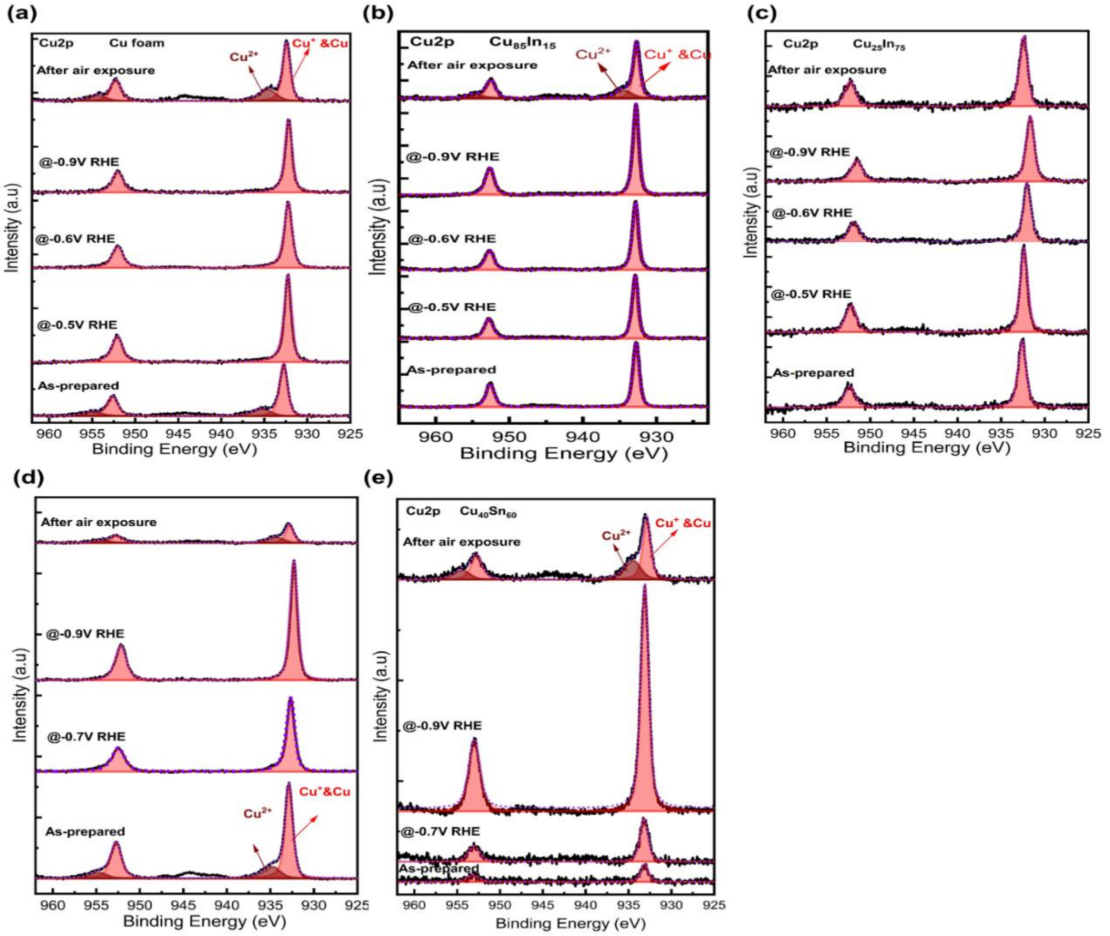

Figure S13. High-resolution Cu 2p XPS spectra of (a) pure Cu foam, (b) $Cu_{85}In_{15}$, (c) $Cu_{25}In_{75}$, (d) $Cu_{85}Sn_{15}$ and (e) $Cu_{40}Sn_{60}$ before and after CO_2 electrolysis in O_2-free glovebox at various cathodic potentials. Besides, we exposed same samples to air after CO_2 electrolysis and measured to validate our glovebox assisted XPS setup.

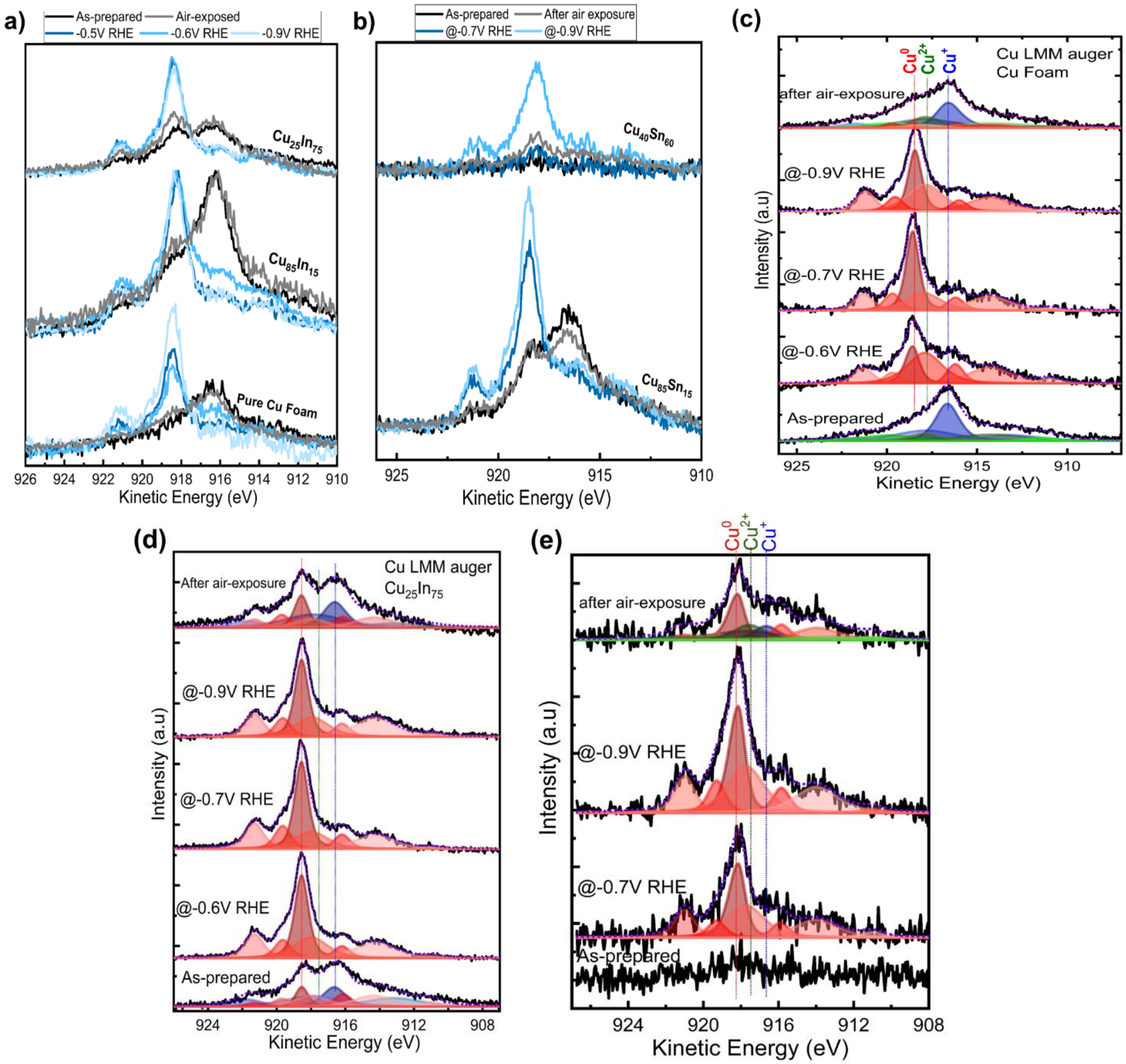

Figure S14. Quasi in-situ XPS for Cu speciation based on Cu LMM auger analysis. Summary of raw data (a-b), and their respective fittings of; (c) pure Cu foam, (d) $Cu_{25}In7_5$ and (e) $Cu_{40}Sn_{60}$ before and after CO_2 electrolysis in O_2-free glovebox at various cathodic potentials. Besides, we exposed same samples to air after CO2 electrolysis and measured to validate our glovebox assisted XPS setup. It is worth mentioning here that we could not fit the Cu LMM spectra of $Cu_{40}Sn_{60}$ before CO2R since, thus we just rely on the Cu 2p spectra to quantify the Cu surface species before CO_2ER. After CO_2 electrolysis at various potentials, noncable Cu LMM spectra were detected for $Cu_{40}Sn_{60}$, suggesting the surface reconstruction under CO_2ER conditions.

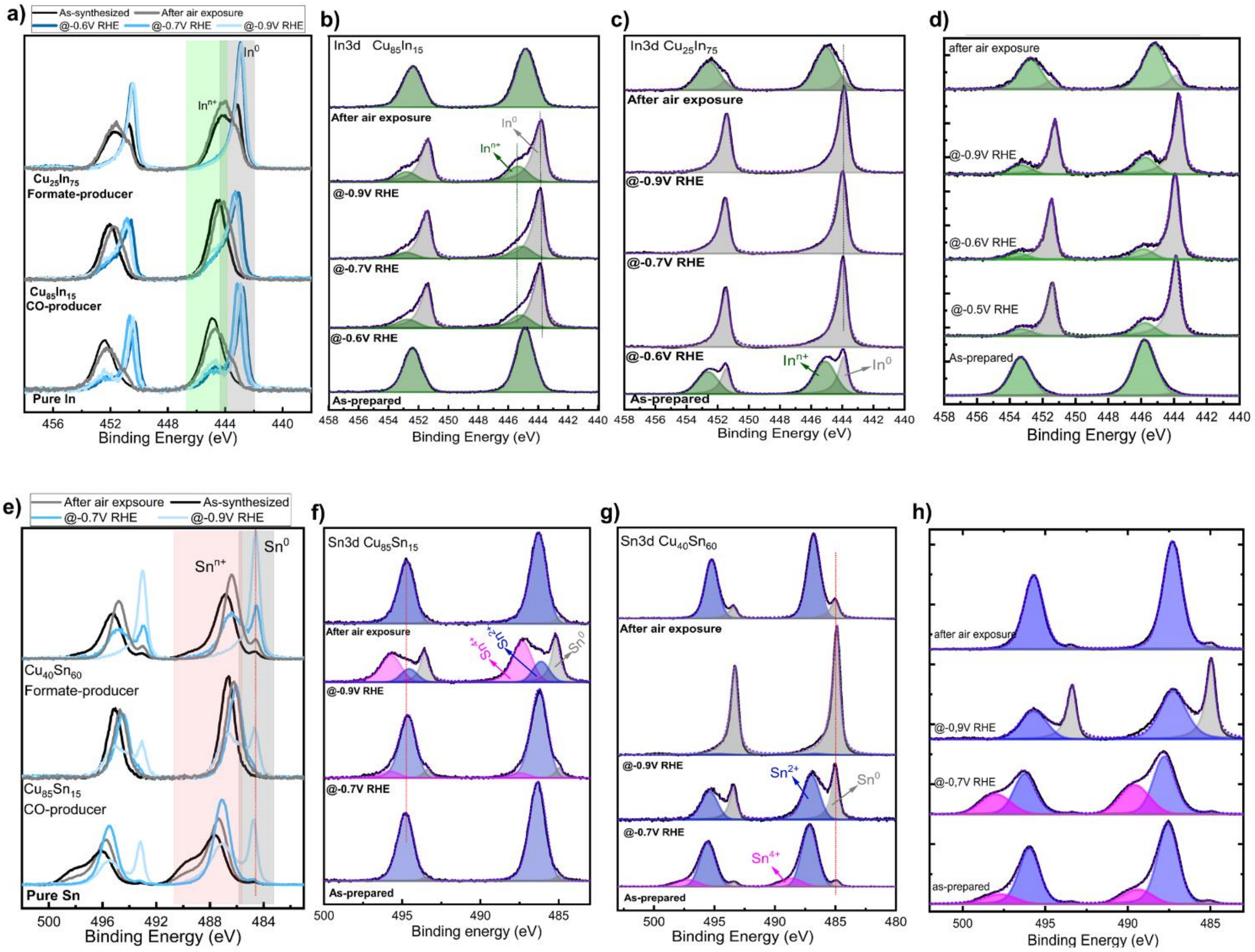

Figure S**15**. Quasi *in-situ* XPS results of In3d (a-d) and Sn3d (e-h) of Cu-In and Cu-Sn bimetallic dendrites. Overlay of the obtained In3d (a) and Sn3d (e) of the Cu-In and Cu-Sn bimetallic foams at various applied potentials without fittings. Representative fittings and of In3d and Sn3d spectra of Cu-In (a-d) and Cu-Sn (e-h) bimetallic foams with best CO and $HCOO^-$ performances, respectively.

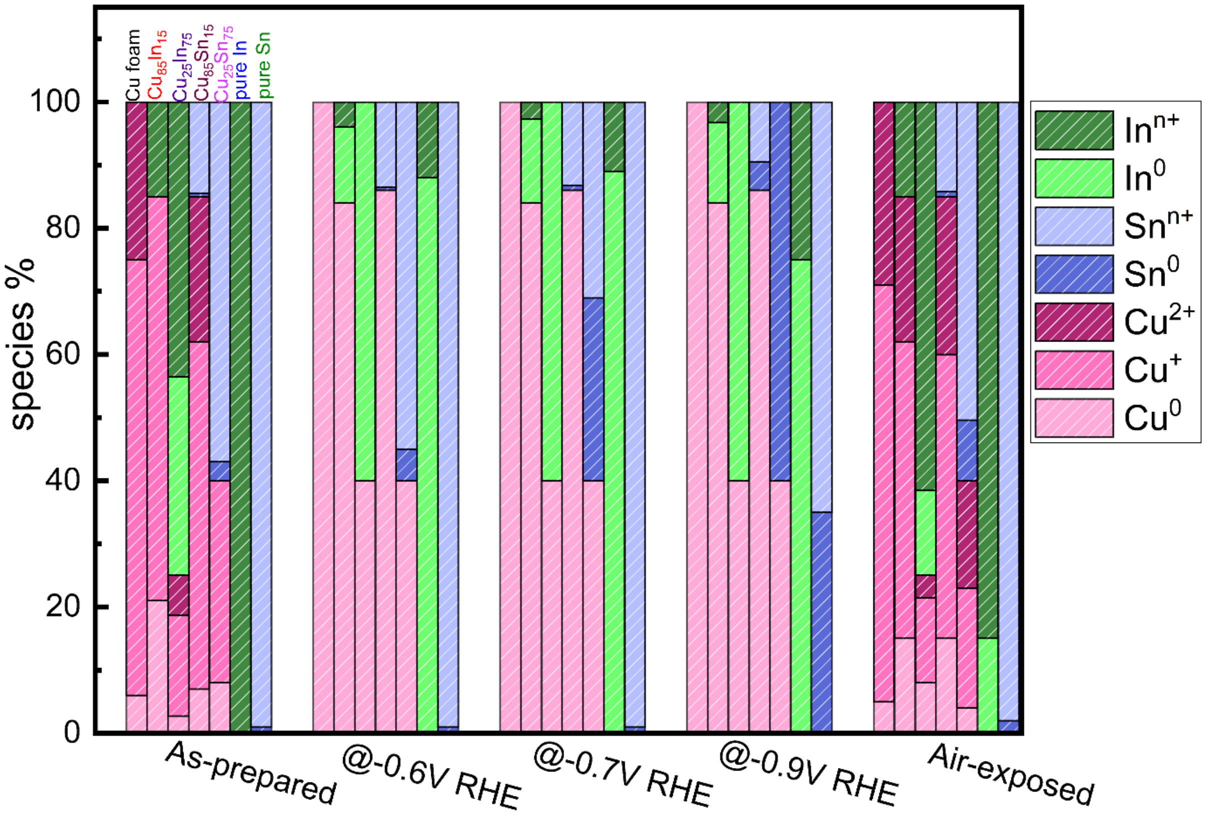

Figure S16. The quantification of various surface speciation as derived from XPS results before CO_2ER and after CO_2ER at various potentials on pure In, Sn and Cu besides bimetallic foams with highest CO- ($Cu_{85}In_{15}$ and $Cu_{85}Sn_{15}$) and $HCOO^-$ ($Cu_{25}In_{75}$ and $Cu_{40}Sn_{60}$) selectivity.

X-ray absorption spectroscopy.

Cu foams, Cu-In and Cu-Sn bimetallic foams were electrodeposited on graphene sheets (25um in thickness, Graphene Supermarket) using dynamic hydrogen bubbling template (DHBT) technique in a two-electrode setup. CO_2 electrolysis was carried out at various potentials in an O_2-free glovebox under inert (N_2) atmosphere using the same H-cell and conditions, which are used to measure the CO2ER performance of the different synthesized catalyst materials as described in detail under **S.1.4**. Then, the operated samples were fixed and covered by Kapton tape in homemade XAS sample holders. Subsequently, the operated samples were transferred out of the glovebox and immediately frozen liquid N2 until prior to XAS measurements.

X-ray absorption spectroscopy (XAS) at the Cu K-edge, Sn L3-edge, In L3-edge were performed at beamline KMC-3 at the BESSY-II synchrotron (Helmholtz Center Berlin, Germany) using a set-up including a Si[111] double-crystal monochromator, a 13-element energy-resolving Si-drift detector (RaySpec) for X-ray fluorescence monitoring, and DXP-XMAP pulse-processing electronics (XIA). Samples were held at 20 K in a liquid-helium cryostat (Oxford). The energy axis of the monochromator was calibrated (accuracy ±0.1 eV) using the K-edge or L_3-edge spectrum of copper, indium, or tin metal foils (fitted reference energy of 8979, 3730, or 3929 eV in the first derivative spectrum). The spot size on the samples was ca. 1.5 x 2.0 mm (vertical x horizontal) as set by a focusing mirror and slits. X-ray fluorescence spectra were collected using a continuous scan mode of the monochromator (scan duration ~10 min). 3 scans were averaged (1-2 scans per sample spot) for signal-to-noise ratio improvement. XAS data were processed (dead-time correction, background subtraction, normalization, self-absorption correction) to yield XANES and Cu-EXAFS spectra using our earlier described procedures and in-house software [7]. k3-weighted EXAFS spectra were simulated with in-house software and phase functions from FEFF9. [8] The range of the fits was 2-14 $Å^{-1}$ for all models, and the amplitude reduction factor ($S0^2(k)$) was 0.8. The errors represent the 68% confidence interval of the respective fit parameter in all EXAFS fit tables. The obtained Rf value of the fits are given for the distance range of interest (1-5 Å of reduced distance). EXAFS simulation results are tabulated in Tables S9-12.

EXAFS fit models:

Since XAS of (inter)metallic phases such as alloys tend to suffer from reabsorption effects and show very strong FT amplitudes for the metallic back scatterers, potentially hiding shells of lighter atoms (e.g., oxygen) or low amounts of other atoms (e.g., In or Sn), efforts have been made in careful data evaluation and thoroughly modeling the data, resulting in a total of almost 180 fit models in EXAFS (see SI for details). With this comprehensive approach, we address the following (interrelated) questions from the XAS-perspective:

(i) Are the metal atoms in the reduced Cu-In/Sn materials present as alloys or separate metallic phases? (ii) What are the Cu/In or Cu/Sn stoichiometries in the alloy phase?

(iii) What is the level of crystallinity? What are the metal oxidation states and oxidic contributions?
(iv) What is the effect of the applied potentials? Are In or Sn leaching or enriching during operation?

We modeled the EXAFS data with and without In or Sn shells and with or without multiple-scattering (MS) shells, the latter providing information on the level of crystallinity. For the intermetallic Cu-In and Cu-Sn samples, this resulted in 13 fit models each (156 models in total). A subsequent meta-analysis of R-values, first-shell distances, and populations, and Cu/In or Cu/Sn ratios disclosed the superior models, which are presented here.

For pure Cu foams the following four models were compared:
1. 4 Cu shells, 1 MS shell (7 parameters)
N fixed to XRD; R: free for 1-3, fixed for 4 and MS; $2\sigma^2$: free for 1-4, fixed for MS
2. 4 Cu shells, 1 MS shell (5 parameters)
N: free, but constrained ratio (XRD); R: free for 1-3, fixed for 4 and MS; $2\sigma^2$: refined conjointly
2a. Set $2\sigma^2$ to 0.007 for all shells (4 parameters)
2b. Set $2\sigma^2$ to 0.007 for all shells, R free for Cu-shell at about 5.1 Å as well as for MS-shell (6 parameters)

For Cu-In and Cu-Sn foams the following 13 models were compared:
1. 4 Cu shells, 1 MS shell (7 parameters)
N fixed to XRD; R: free for 1-3, fixed for 4 and MS; $2\sigma^2$: free for 1-4, fixed for MS
2. 4 Cu shells, 1 MS shell, 1 In/Sn shell (7 parameters)
N: free, but constrained ratio (XRD); R: free for 1-3 and In/Sn, fixed for 4 and MS; $2\sigma^2$: refined conjointly
2a. Set $2\sigma^2$ to 0.007 for all shells (6 parameters)
2b. Set $2\sigma^2$ to 0.007 for all shells, R free for Cu-shell at about 5.1 Å as well as for MS-shell (8 parameters)
3. 4 Cu shells, 1 MS shell, 1 In/Sn shell (8 parameters)
N: constrained ratio (XRD); R: free for 1-3 and In/Sn, fixed for 4 and MS; $2\sigma^2$: refined conjointly for Cu, free for In/Sn
3a. Set $2\sigma^2$ to 0.007 for Cu shell, free for In/Sn shell (7 parameters)
4. 4 Cu shells (9 parameters)
N, R: free; $2\sigma^2$: refined conjointly
5. 5 Cu shells (11 parameters)
Extra Cu shell; N, R: free; $2\sigma^2$: refined conjointly
6. 4 Cu shells, 1 In/Sn shell (11 parameters)
Extra In/Sn shell; N, R: free; $2\sigma^2$: refined conjointly
6a. Set $2\sigma^2$ to 0.007 for Cu+In/Sn shells (10 parameters)
7. 4 Cu shells, 1 In/Sn shell (12 parameters)
Extra In/Sn shell; N, R: free; $2\sigma^2$: refined conjointly for Cu; In/Sn free
7a. Set $2\sigma^2$ to 0.007 for Cu shell, free for In/Sn shell (11 parameters)

8. 4 Cu shells, 1 In/Sn shell (11 parameters)

Extra In/Sn shell; N:free for Cu, constrained ICP-ratio for In/Sn; R: free; $2\sigma^2$: refined conjointly for Cu; In/Sn free

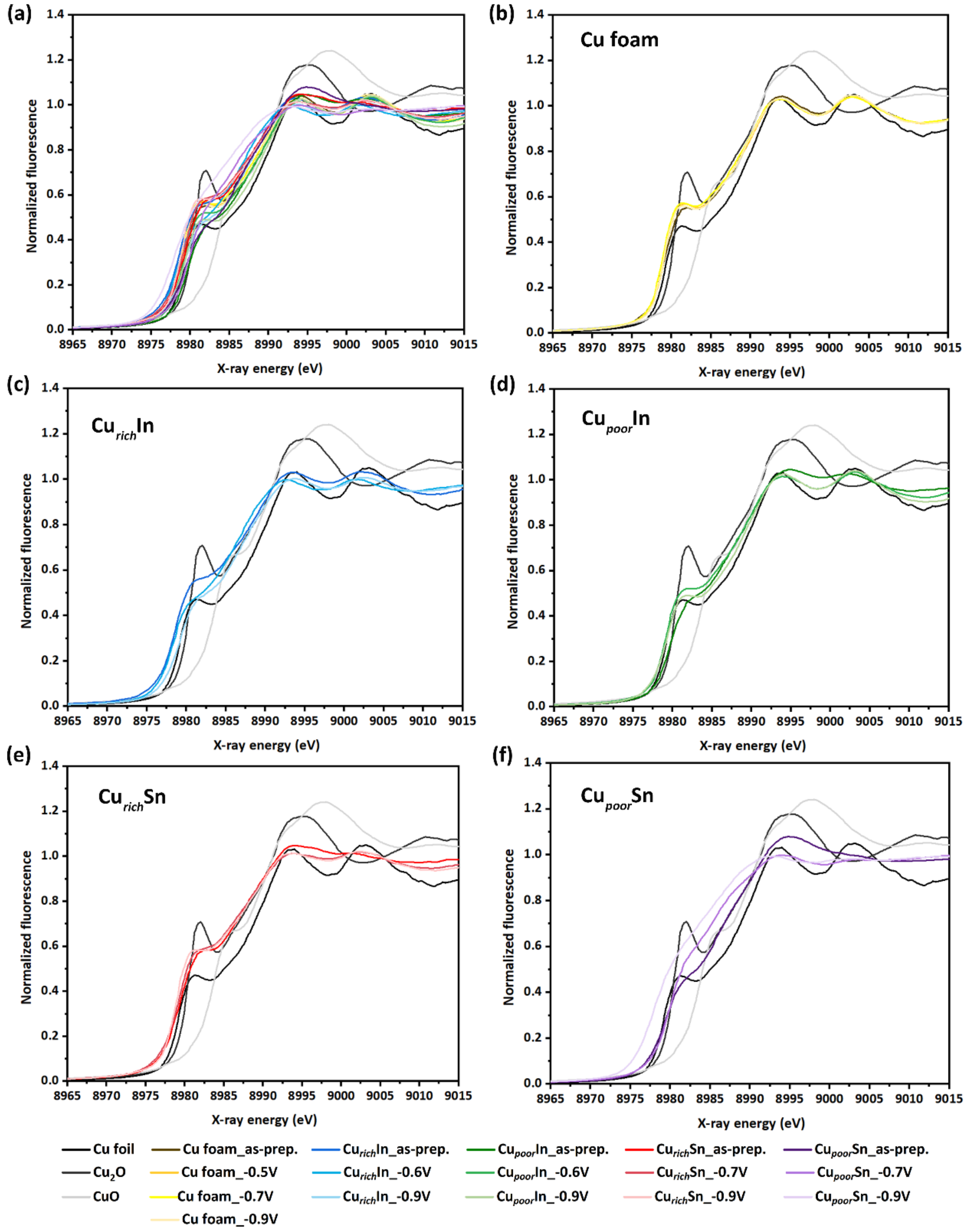

Figure S17. Cu K-edge XANES for (a) all samples, (b) Cu foam, (c) In-poor Cu foam, (d) In-rich Cu foam, (e) Sn-poor Cu foam, and (f) Sn-rich Cu-foam. Each sample type is shows in as-prepared state (dark color) and after application of different reductive potentials (vs. RHE, increasingly light colors). Cu foil (black), Cu_2O (medium gray), and CuO (light gray) are given as references.

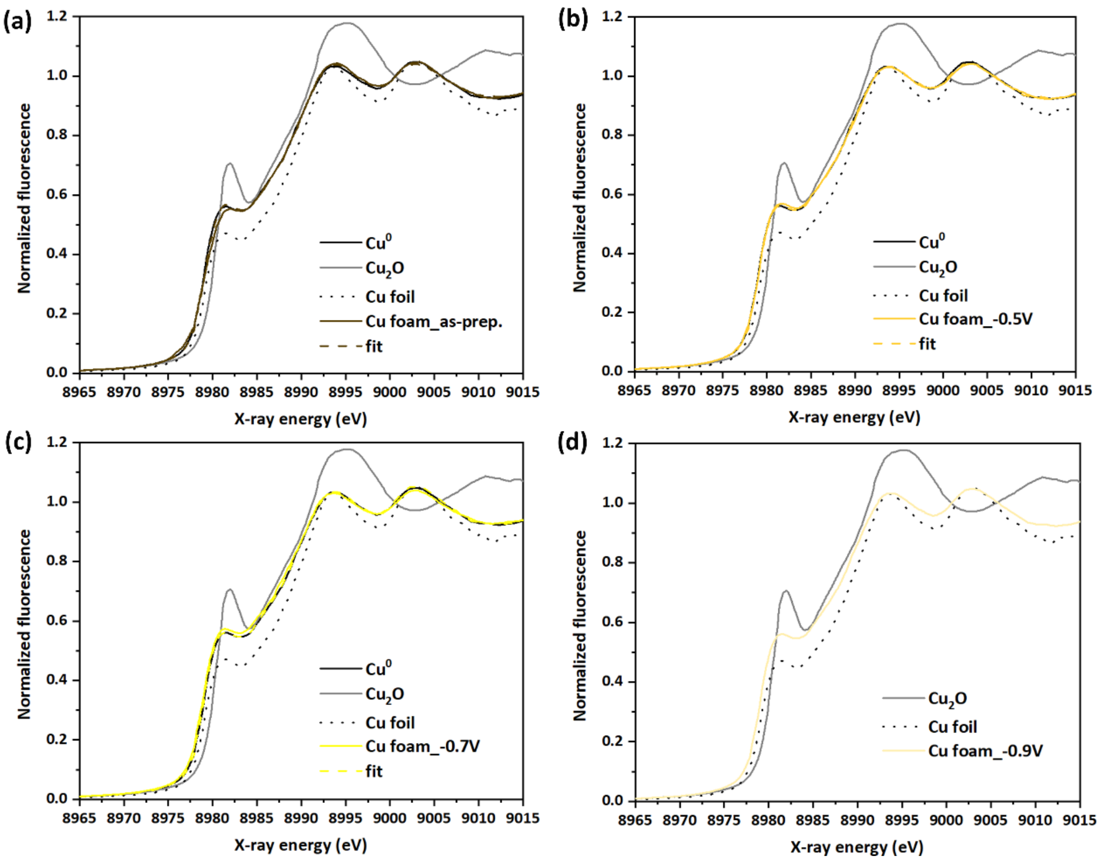

Figure S18. Cu K-edge XANES- for Cu foams. Since Cu foam looks neither like Cu foil or Cu_2O, nor like a combination of both, the Cu foam reduced at -0.9 V was considered to be the most proper reference for pure/non-oxidized Cu foam (labeled as Cu^0). Accordingly, using Cu^0 and Cu_2O for linear combination (shown here) resulted in superior fits compared to those including Cu foil (not shown), see Tables S1-S3. The fits imply 6% oxidation for the as-prepared sample (a), 3% oxidation for the sample at -0.5 V vs RHE (b), and full conversion to Cu at higher reducing potentials (c and d), see also Table S3.

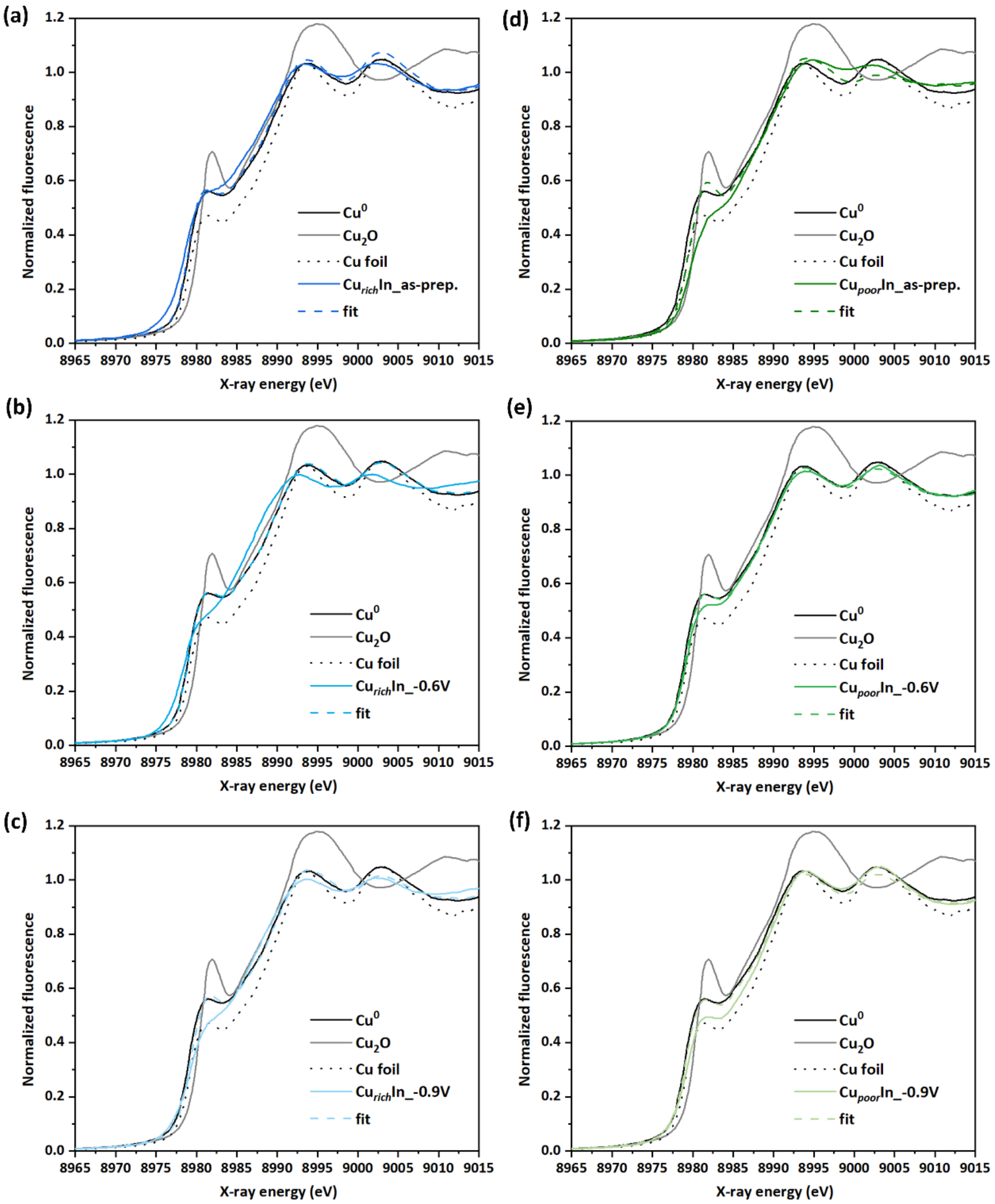

Figure S19. Cu K-edge XANES for Cu foams containing In. Fit lines stem from the linear combination of Cu^0 and Cu_2O, see also Table S4.

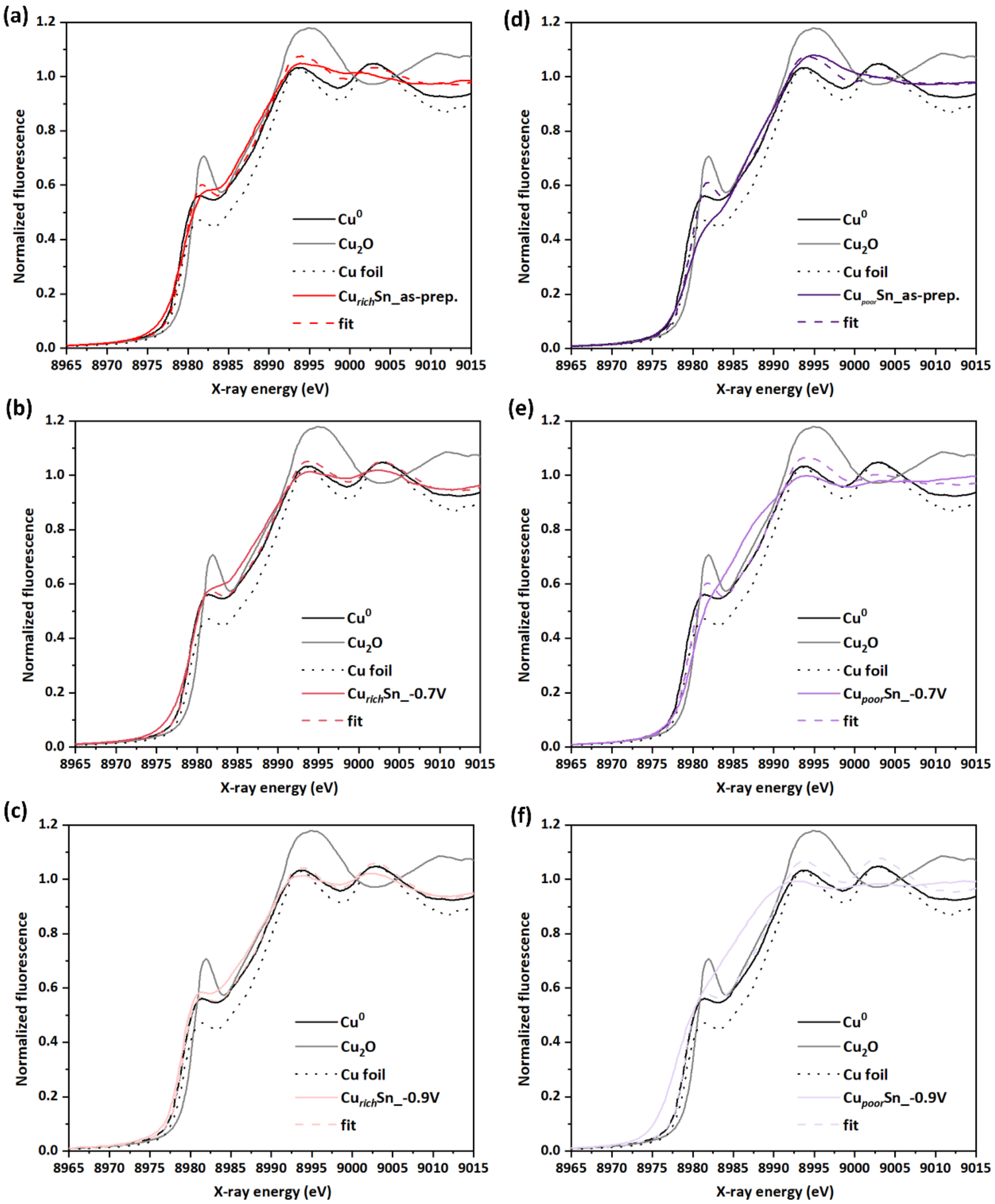

Figure S20. Cu K-edge XANES for Cu foams containing Sn. Fit lines stem from the linear combination of Cu^0 and Cu_2O, see also Table S5.

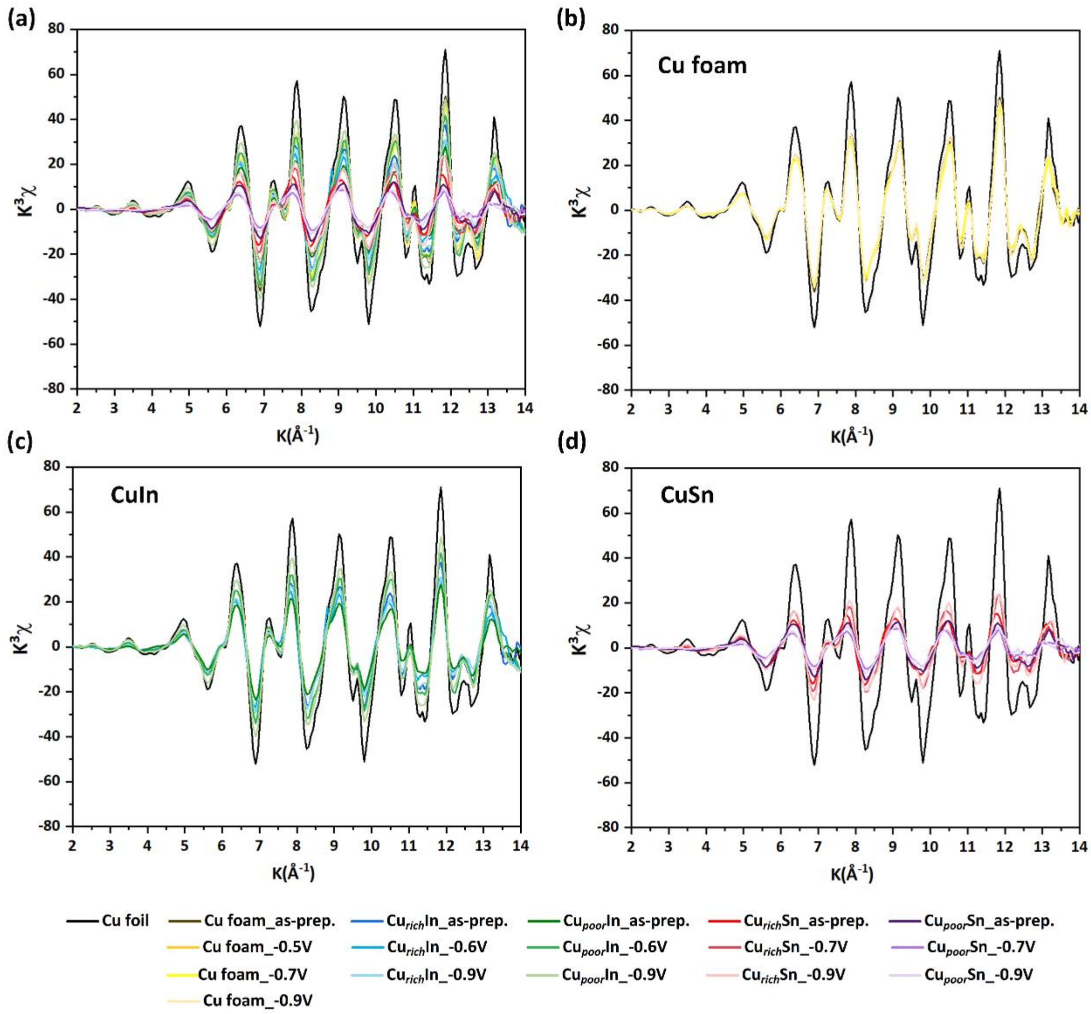

Figure S21. Cu K-edge EXAFS k-space representation for (a) all samples, (b) Cu foam, (c) Cu-In foam, and (d) Cu-Sn foam. Cu foil (black line) is given as reference. Pronounced oscillations are obtained for Cu foam and Cu-In foam, suggesting considerable degrees of crystallinity, whereas much lower oscillations are obtained for Cu-Sn foam, suggesting a rather amorphous structure. For Cu-In and Cu-Sn foams there is a tendency for increased crystallinity after application of reductive potentials.

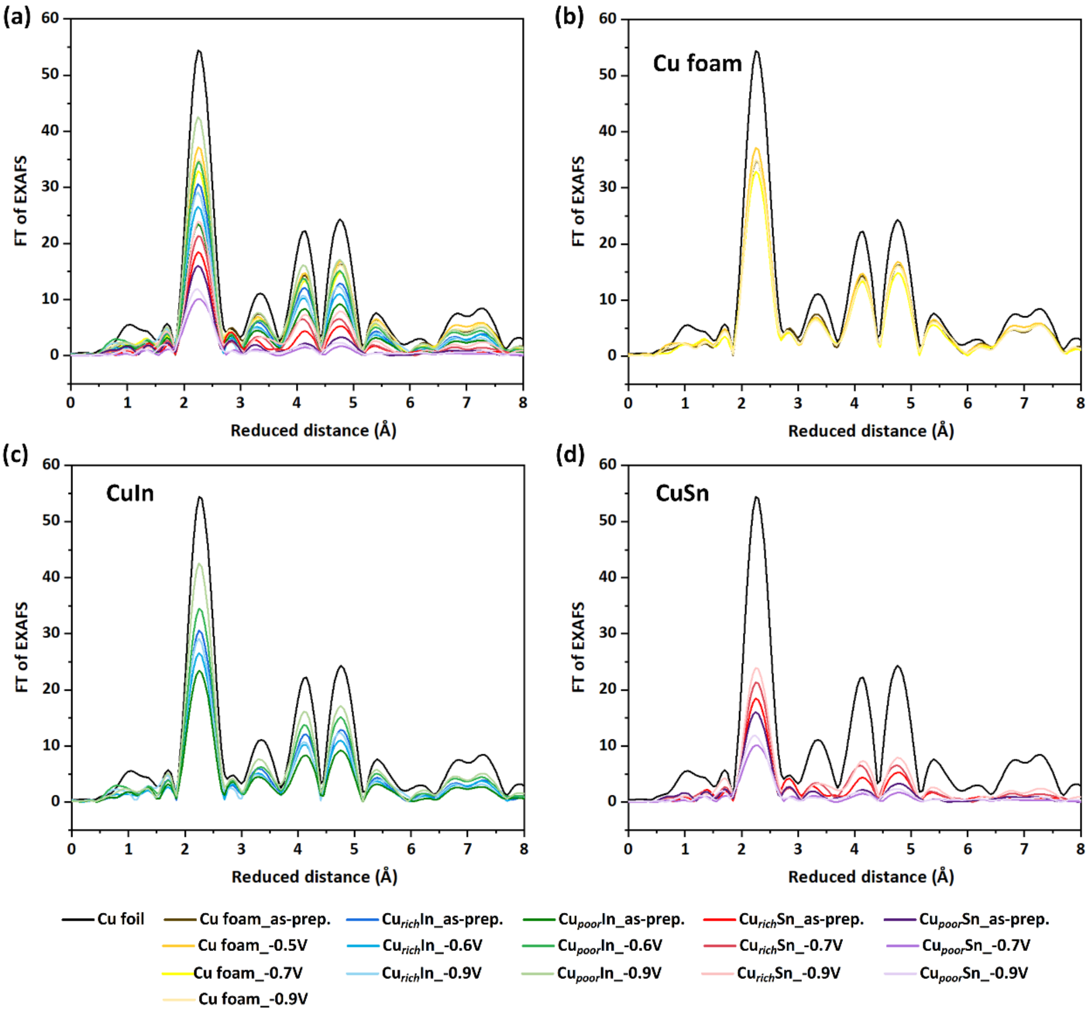

Figure S22. Cu K-edge EXAFS FT representation for (a) all samples, (b) Cu foam, (c) Cu-In foam, and (d) Cu-Sn foam. Cu foil (black line) is given as reference. Pronounced oscillations are obtained for Cu foam and Cu-In foam, suggesting considerable degrees of crystallinity, whereas much lower oscillations are obtained for Cu-Sn foam, suggesting a rather amorphous structure. For Cu-In and Cu-Sn foams there is a tendency for increased crystallinity after application of reductive potentials.

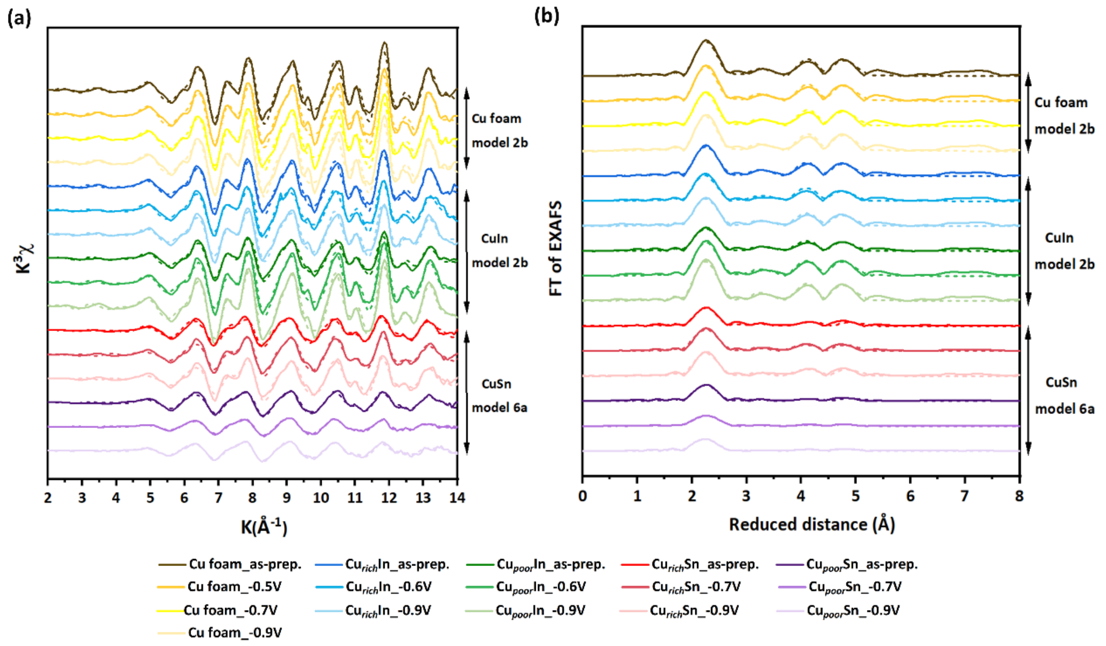

Figure S23. Cu K-edge EXAFS of all samples, (a) k-space, (b) FT of EXAFS. Fit lines refer to the best models, see also Table S9, suggesting considerable degrees of crystallinity for Cu foam and Cu-In foam (model 2b), but low levels for Cu-Sn foam (model 6a).

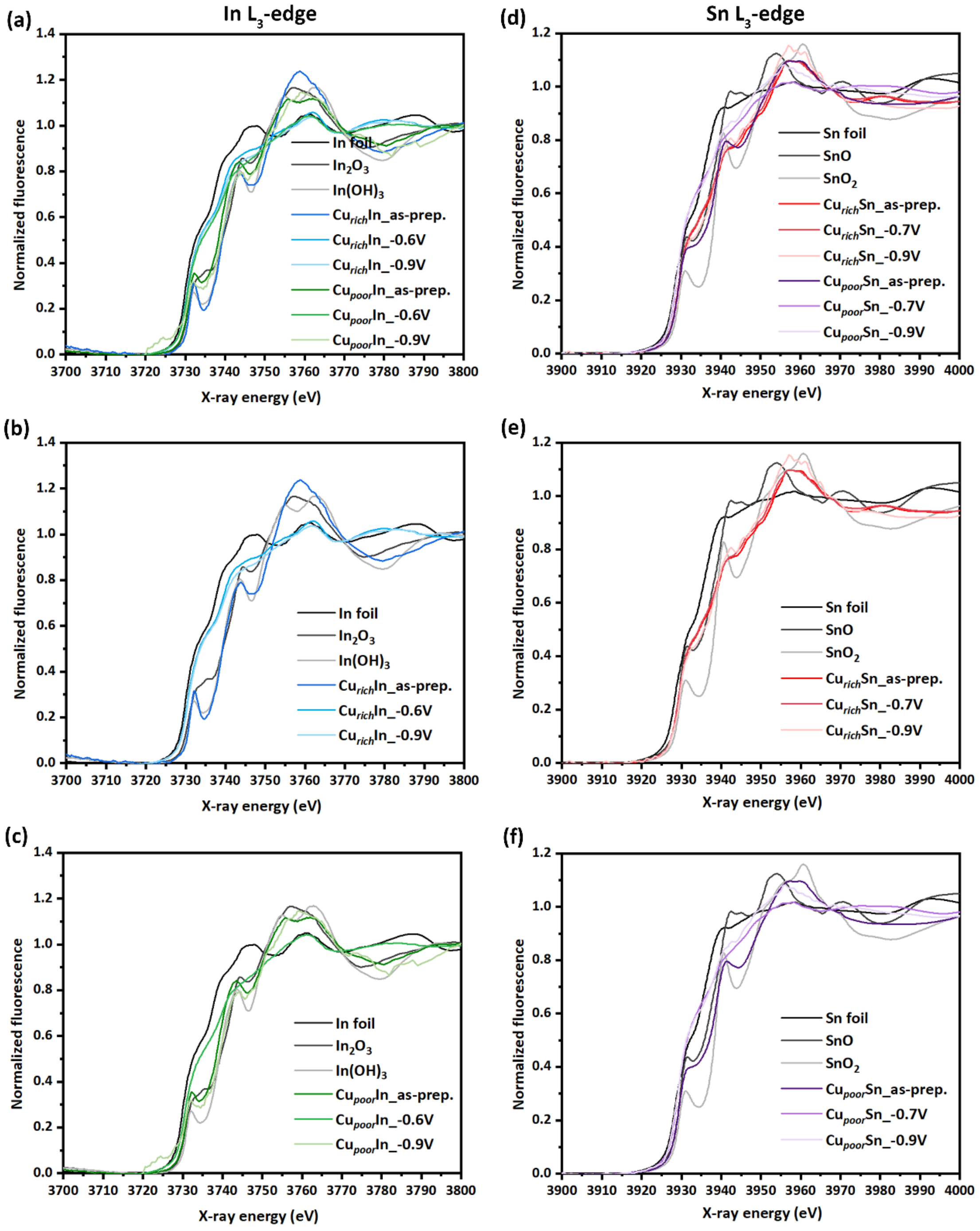

Figure S24. In and Sn L_3-edge XANES for (a) all Cu-In samples, (b) Cu-rich Cu-In, (c) Cu-poor Cu-In, (d) all Cu-Sn samples, (e) Cu-rich Cu-Sn, and (f) Cu-poor Cu-Sn. Cu-In samples can be represented as linear combination of In foil and In_2O_3, whereas Cu-Sn samples as superposition of Sn foil and SnO_2. Pronounced oxidation state changes are visible after application of reducing potentials.

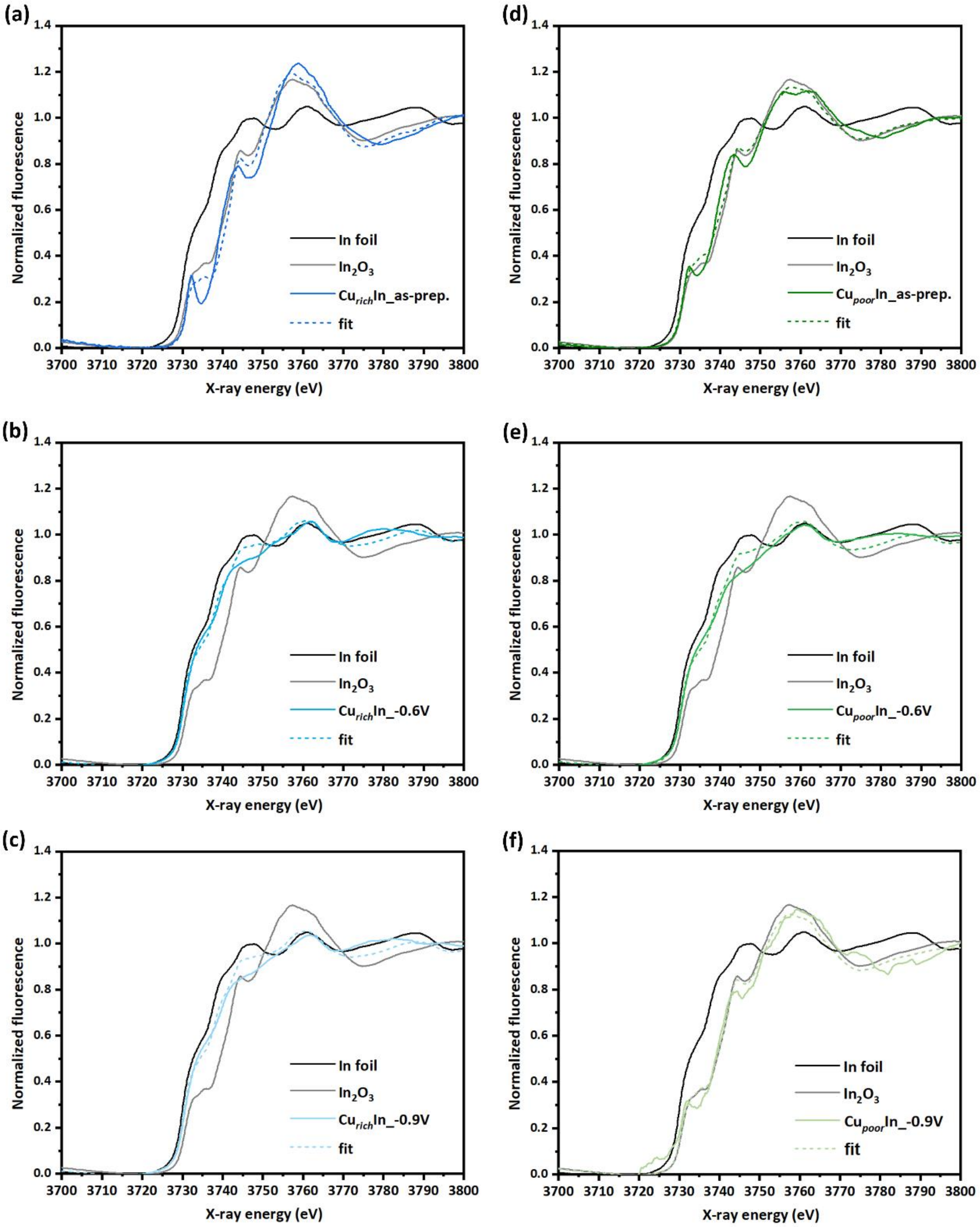

Figure S25. In L_3-edge XANES for (a) Cu rich as-prepared Cu-In, (b) Cu rich Cu-In after-0.6 V vs. RHE for 1h, (c) Cu rich Cu-In after -0.9 V vs RHE for 1h, (d) Cu poor as-prepared Cu-In, and (e) Cu poor Cu-In after -0.6 V vs RHE for 1h, (f) Cu poor Cu-In after -0.9 V vs RHE for 1h. Linear combination of In foil and In_2O_3 suggest 100% In_2O_3 for Cu rich as prepared Cu-In and 84% for the corresponding Cu poor sample, and 65-76% In foil character after application of reductive potentials, see also Table S4.

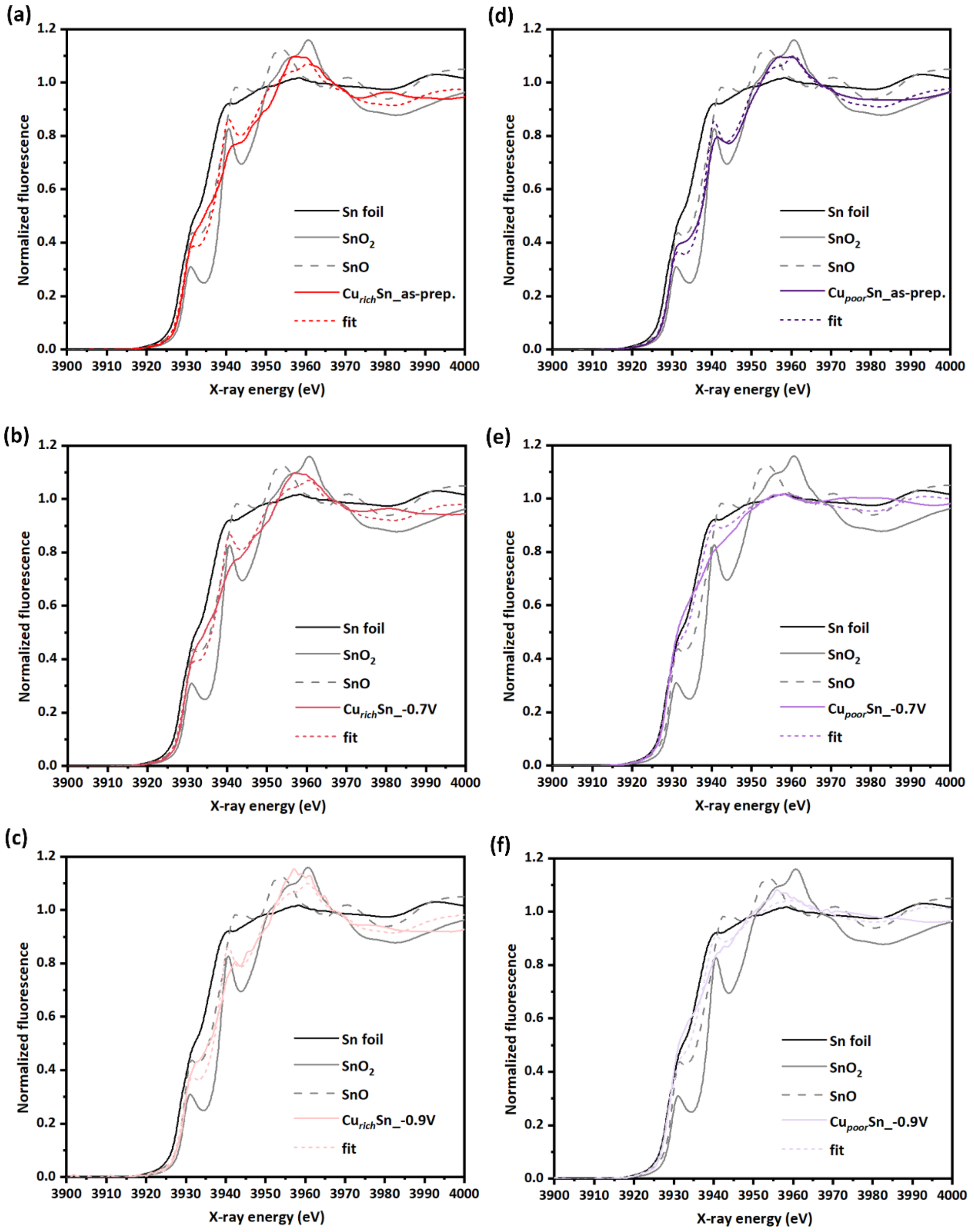

Figure S26. Sn L_3-edge XANES for (a) Cu rich as-prepared Cu-Sn, (b) Cu rich Cu-Sn at -0.6 V vs. RHE, (c) after -0.9 V vs RHE, (d) Cu poor as-prepared Cu-Sn, (e) Cu poor Cu-Sn at -0.6 V vs RHE, and (f) after -0.9 V vs RHE. Linear combination of Sn foil and SnO_2 suggest 50% SnO_2 for Cu rich as prepared Cu-Sn and 64% for the corresponding Cu poor sample. After application of reductive potentials, Cu-rich Cu-Sn samples are rather unaffected of even slightly oxidized, whereas Cu-poor Cu-Sn samples show 81-88% Sn foil character, see also Table S5.

Details and results for Figures S14-26:

Linear combination techniques indicate almost 100% In_2O_3 content for $Cu_{85}In_{15}$ (Cu-rich) as-prepared samples and a ratio of about 85:15 (In^{2+} : In^0) for $Cu_{25}In_{75}$ (Cu-poor) as-prepared samples (Table S4). To the contrary, as-prepared Sn-spectra of Cu-Sn samples can only be modeled by superposition of Sn-foil, and SnO_2 (instead of SnO, **Figure S26** and Table S5), with ratios of about 36:64 (Sn:SnO_2) for as-prepared $Cu_{40}Sn_{60}$ samples and 49:50 for as-prepared $Cu_{85}Sn_{15}$ samples, hinting towards an average oxidation state around 2 for Sn. Whereas little to no response to applied potentials was observed for the Sn-edge in $Cu_{85}Sn_{15}$ (**Figure S24e**), the oxide-to-foil-trend obtained for the Sn-spectra is also visible for the Cu-poor case ($Cu_{40}Sn_{60}$, **Figure S24f**), which again suggests that most of the Sn atoms are incorporated into the Cu-phase for the Cu-rich case ($Cu_{85}Sn_{15}$), but a separate Sn(Ox)-phase might have formed for the Cu-poor case ($Cu_{40}Sn_{60}$).

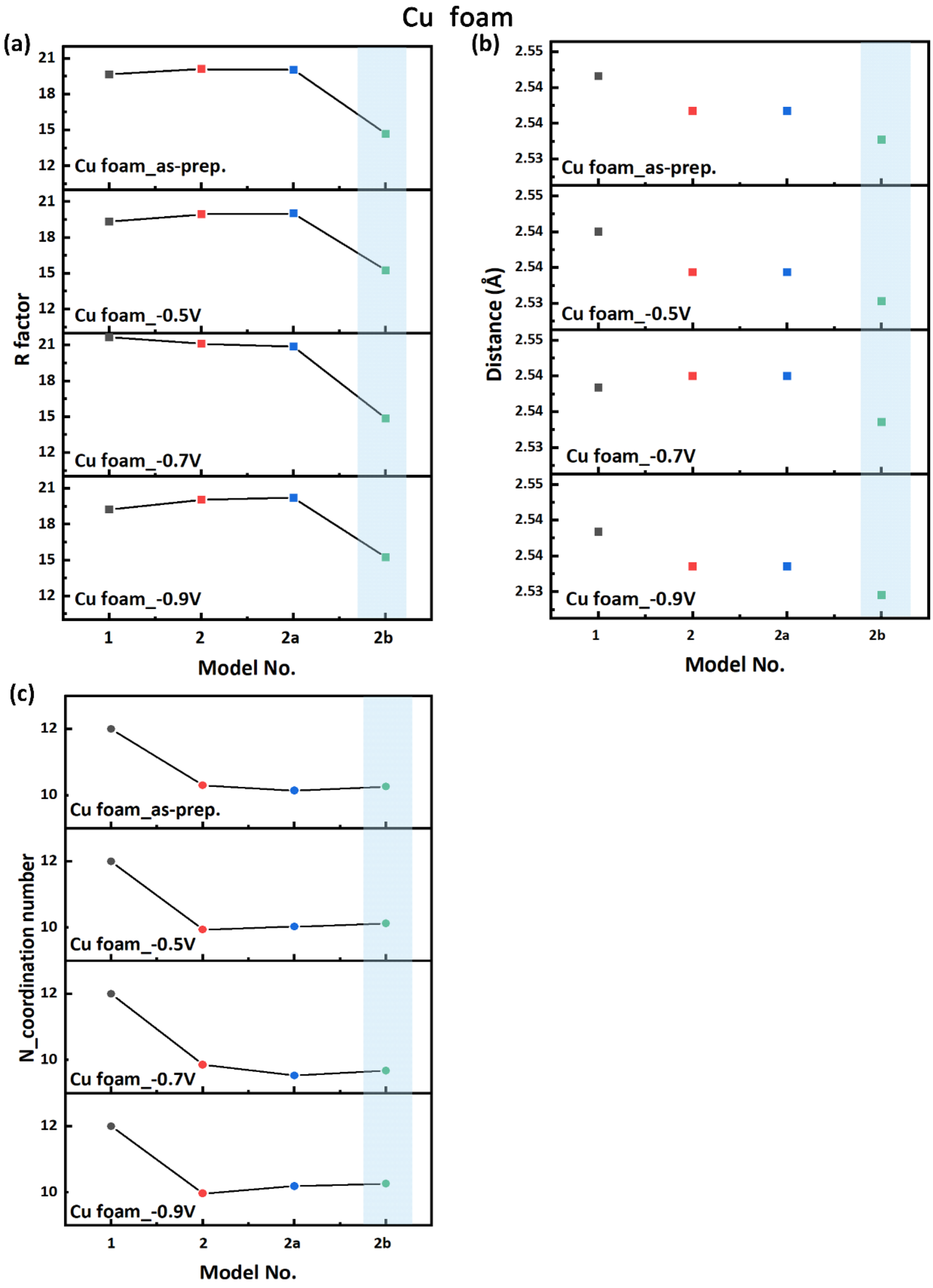

Figure S27. Comparison of EXAFS fit models for the Cu foams, see also Tables S9 and S10. The preferred model (2b) is highlighted by a blue vertical bar.

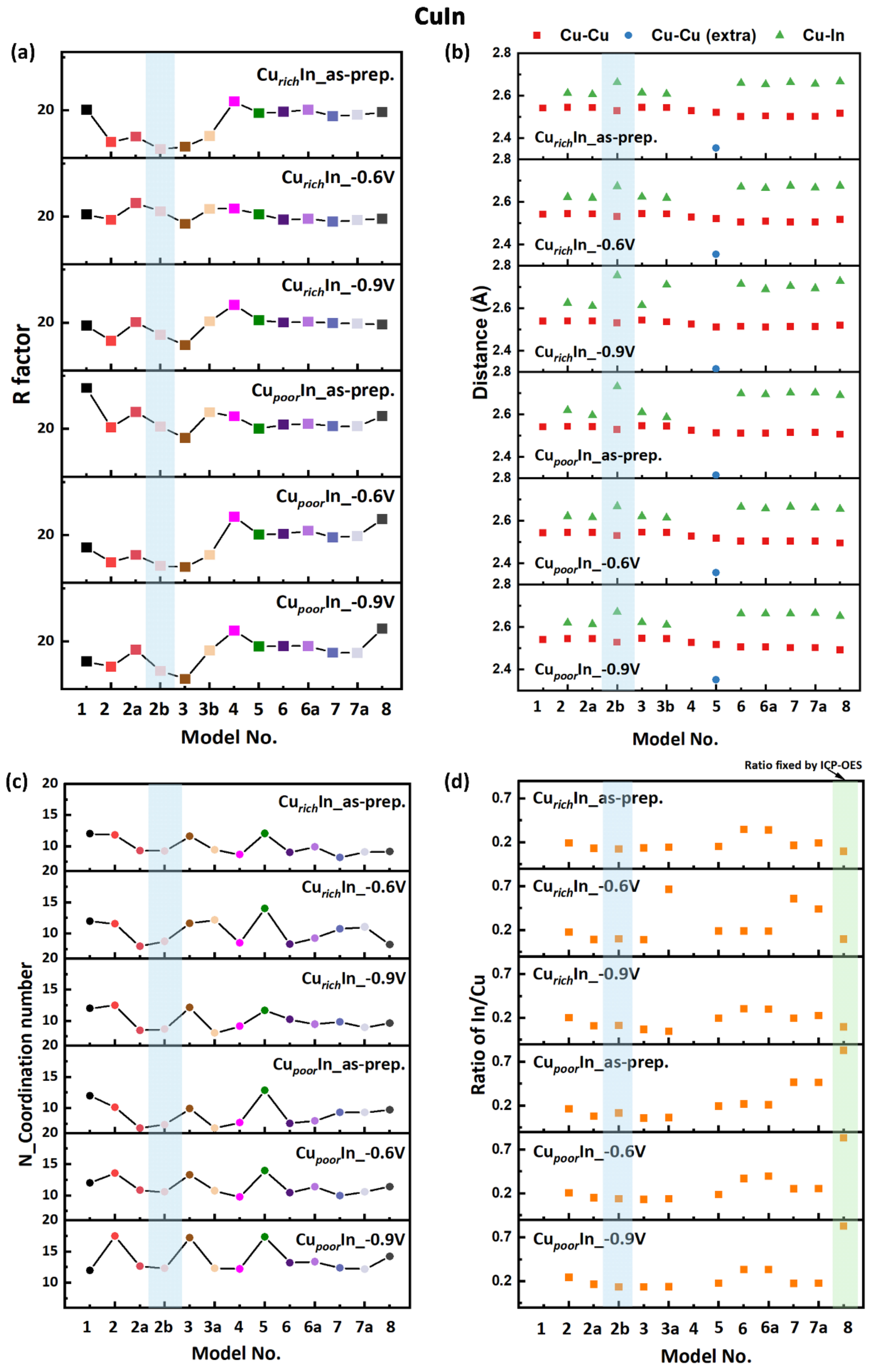

Figure S28. Comparison of the EXAFS fit models (Cu K-edge) of the six Cu-In foams. (a) fit R-factor, (b) first shell distances including Cu-Cu and Cu-In, (c) coordination number N of the first shell including Cu-Cu and Cu-In, and (d) ratio of N for the Cu-In and Cu-Cu shells. The preferred model (2b) is highlighted by a blue vertical bar, clearly suggesting the considerable degree of crystallinity in the Cu-In foams.

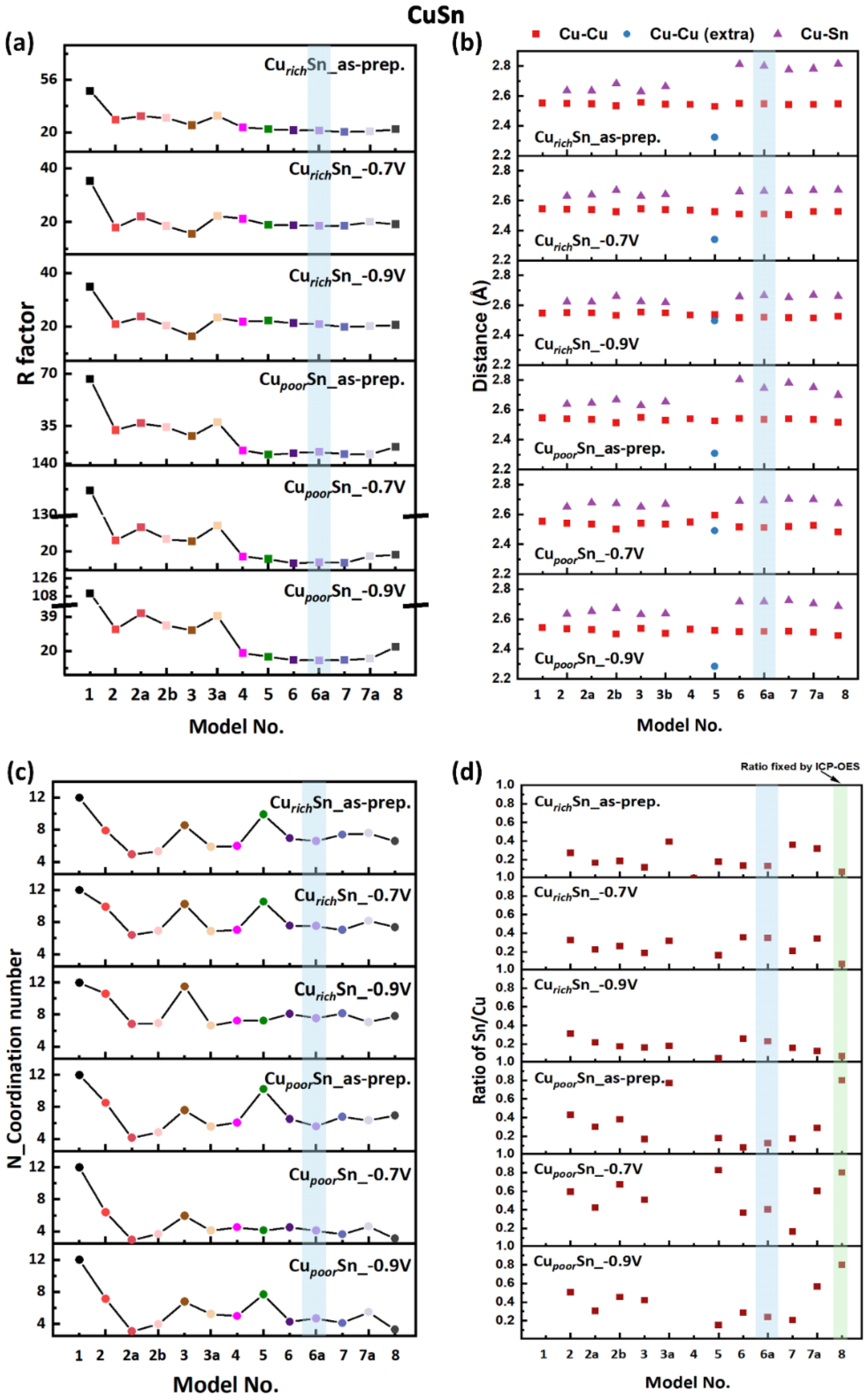

Figure S29. Comparison of the EXAFS fit models (Cu K-edge) of the six Cu-Sn foams. (a) fit R-factor, (b) first shell distances including Cu-Cu and Cu-Sn, (c) coordination number N of the first shell including Cu-Cu and Cu-Sn, and (d) ratio of N for the Cu-Sn and Cu-Cu shells. A blue vertical bar, clearly suggesting a low degree of crystallinity in the Cu-Sn foams highlights the preferred model (6a).

In-situ Raman measurements:

Raman spectra were collected with a Renishaw inVia Raman spectrometer coupled with a Leica microscope. Calibration was conducted using a silicon wafer standard (521 cm^{-1}). A water immersion objective (Leica, 40×, numerical aperture of 0.8) was used to focus and collect the incident and scattered laser light. The excitation source was a 633 nm laser (He-Ne laser, 10% power intensity), which was not focused on a circular spot, but on a line of about 100 μm length ("streamline mode" of the inVia spectrometer), to reduce the laser power per irradiated area. A grating of 1200 lines/mm and a slit size of 20 μm was used. A home-built electrochemical cell made of PTFE was interfaced with the Raman microscope for spectroscopic measurements. The cell was equipped with a saturated Ag/AgCl reference electrode and a Pt-ring counter electrode and controlled by a SP-200 Biologic potentiostat. Electrochemical CO_2 reduction was performed on deposited foam surface in 0.1 M $KHCO_3$ electrolyte saturated with CO_2 (pH 6.8), and the electrolyte was continuously purged with CO_2 throughout the experiment.

(1) Two spectra were collected for as-prepared samples in dry state and OCP condition. (2) The as-prepared samples were pre-activated by applying a constant current of -2.5 mA/cm^2 until the electrode potential reached -0.4V vs RHE. (3) A series of spectra at different time scales (2 min, 5 min, 10 min, etc.) was collected during the pre-activation step. (4) The samples were measured by applying the potential of interest (-0.4V to -0.9V vs RHE) for 2 h. (5) When the measurements at the selected potentials were completed, a series of spectra were collected at OCP. (6) The samples were transferred and exposed to air. (7) A spectrum was acquired after air-exposure.

In all experiments, five accumulations were averaged per spectrum; the data collection time was 5 s per accumulation. The data were collected during applying constant potential for one hour via acquiring 5 spectra (each spectrum requires ~35 s) at different time scales (5 min, 10 min, etc). All spectra were smoothed, and baseline corrected by Renishaw software. Fitting of the baseline is done after SERS peaks were automatically subtracted from the raw data spectra. The baseline consisted of a polynomial function with degrees between 8and 11, and noise tolerance level around 1.4 - 1.6. The degree of the polynomial function depended strongly on the amount of SERS peaks present. Normalization was done by averaging the intensity of the background curve and this averaged intensity was used to divide the background subtracted and smoothed spectra.

For local pH: A 473 nm laser (YAG laser, 100% power intensity) focusing on a line (~30×5 μm) served as an excitation source. To acquire spectra at different distances from the catalyst surface, depth scans were performed at each potential, employing the line-focus option of the inVia Raman spectrometer. The focusing point on the catalyst surface was set as zero position. The acquisition time for a spectrum with 5 accumulations (20s exposure time) was around 140 s.[9]

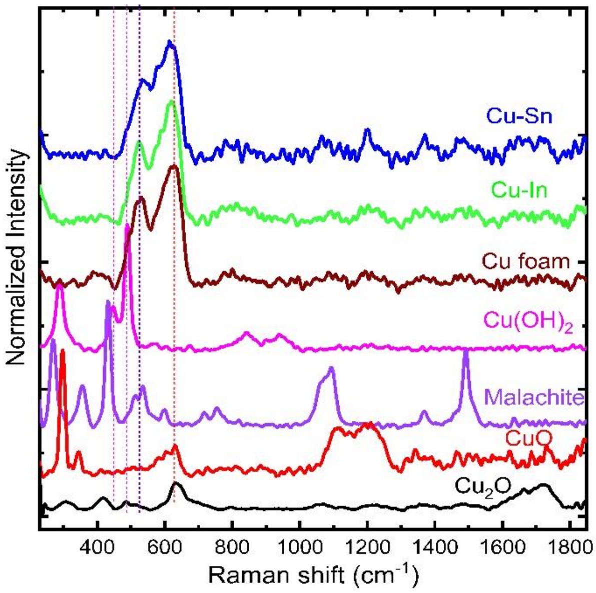

Figure S30. Raman spectra for various Cu standards (Cu_2O, CuO, $Cu(OH)_2$ and malachite) and as-prepared pure Cu and Cu-In and Cu-Sn bimetallic foams

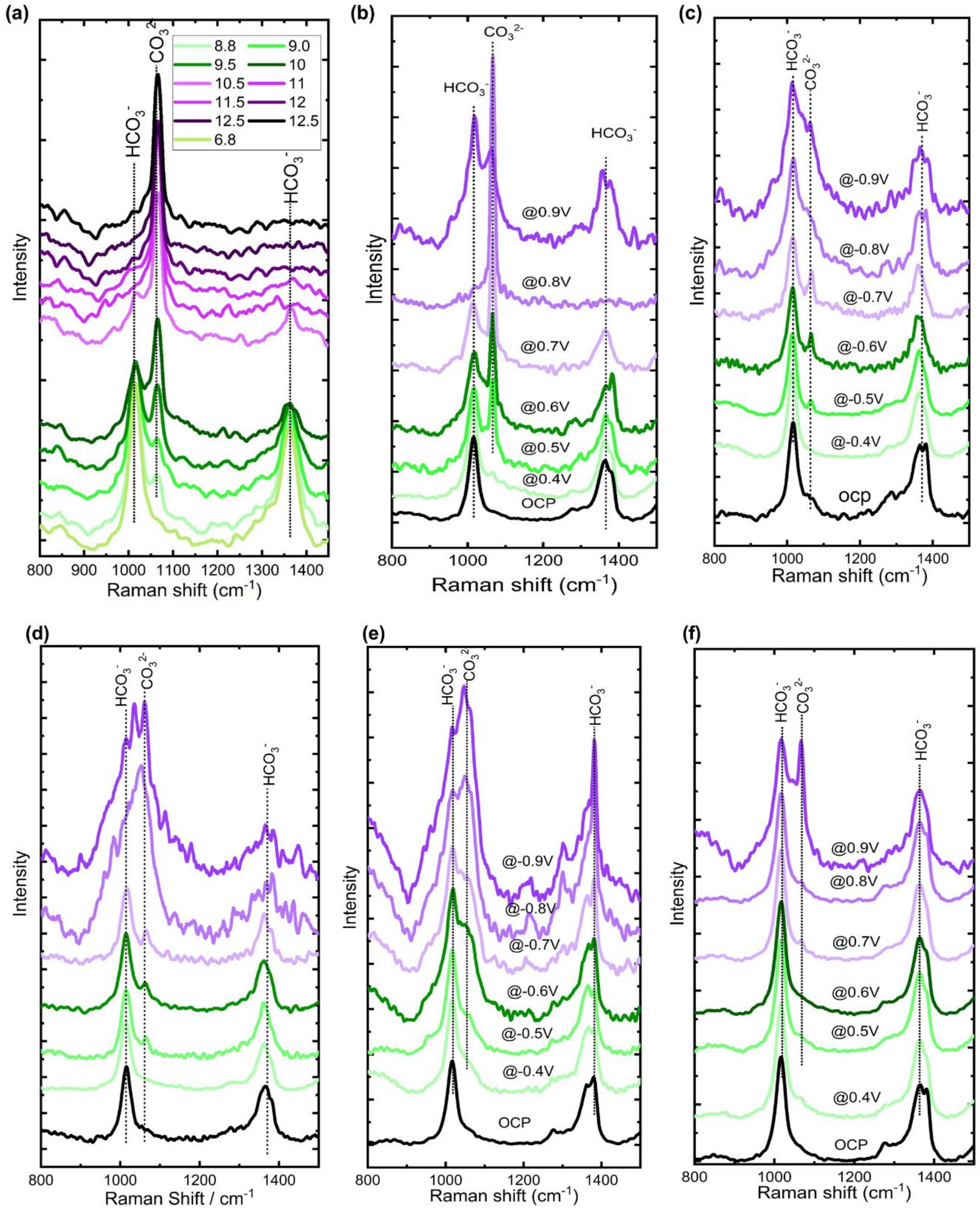

Figure S31. Raman measurements of various pH standards ((a), for comparison) and various as-prepared catalyst materials; (b) pure Cu foam, (c) $Cu_{85}In_{15}$, (d) $Cu_{85}Sn_{15}$, (e) $Cu_{25}In_{75}$ and (f) $Cu_{40}Sn_{60}$. Same electrochemistry protocol was applied as described in the experimental section.

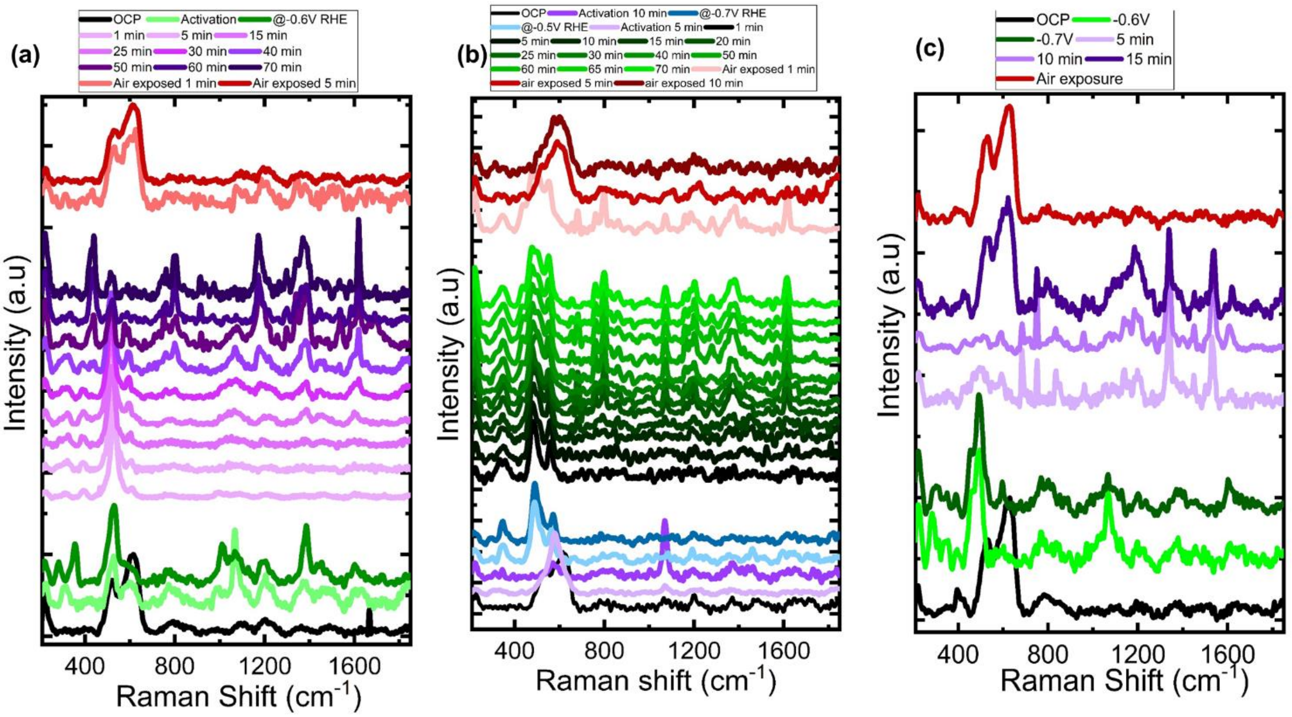

Figure S32. Raman spectra of (a) $Cu_{85}In_{15}$, (b) $Cu_{85}Sn_{15}$ and (c) pure Cu foam, after bias removal and keeping them in same electrolyte or exposed them to air.

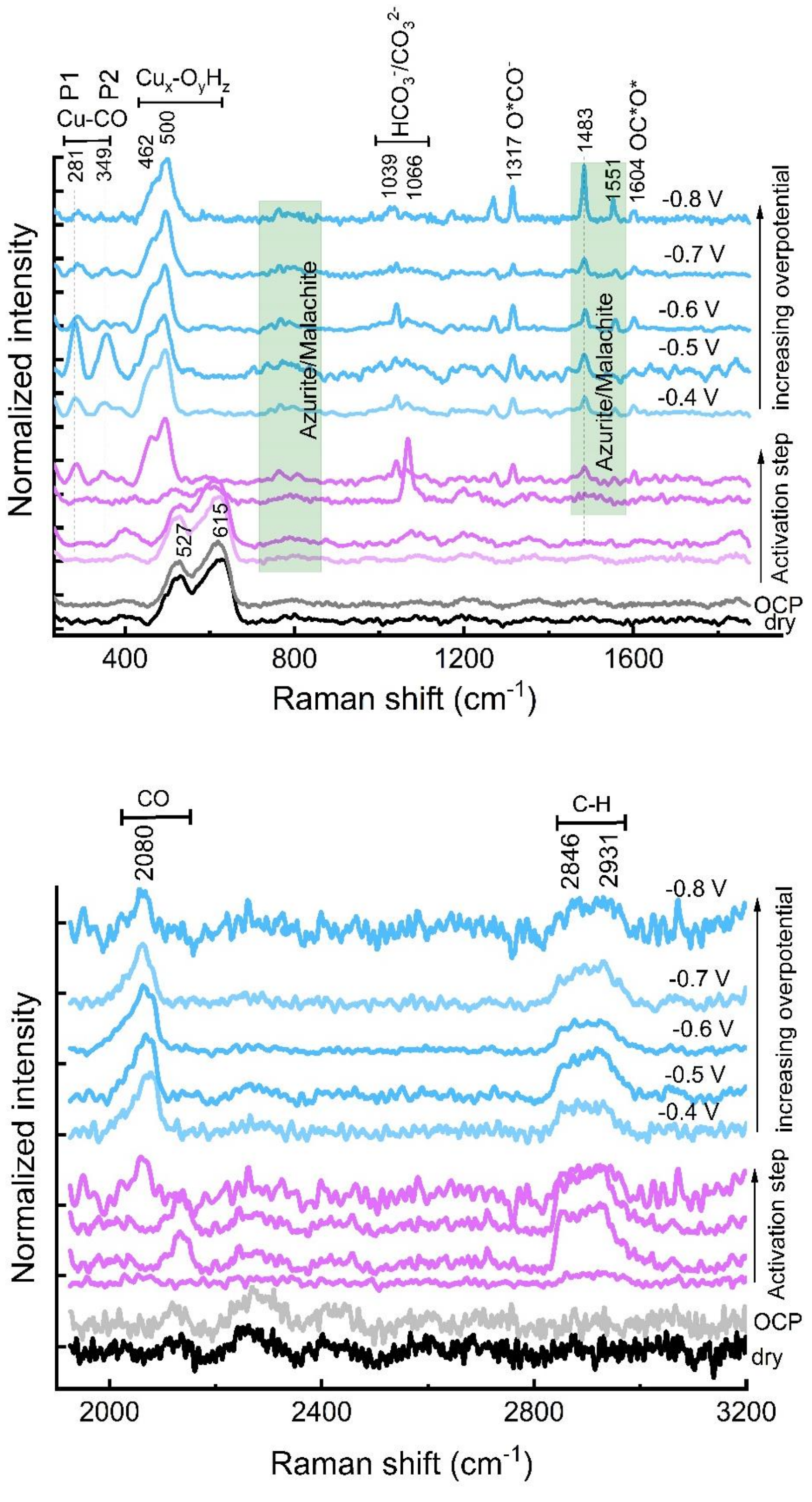

Figure S33. In-situ SERS obtained at pure Cu foam during the activation step (applying -2mA/cm^2 for 15 minutes) and at various applied potentials (from -0.4 V to -0.9 V) in CO_2-saturated 0.1 M $KHCO_3$.

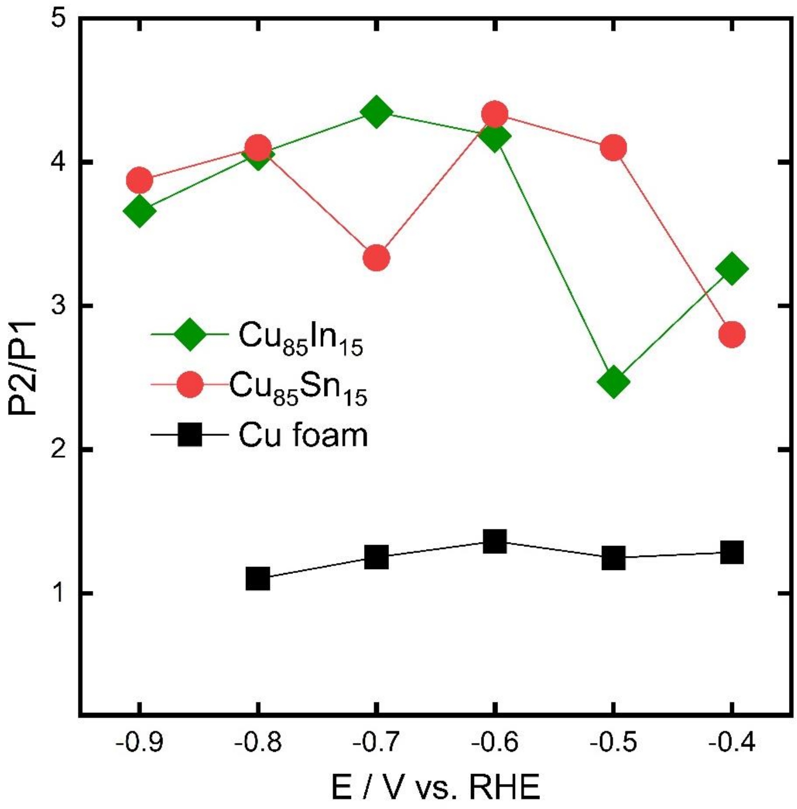

Figure S34. the variation of the P2/P1 ratio as function of the applied cathodic potential of Cu foam and CO-selective bimetallic catalysts *($Cu_{85}In_{15}$ and $Cu_{85}Sn_{15}$).*

Table S1. Cu XANES fit of samples using three different references

	Ref_Cu_2O	Ref_Cu^0	Ref_Cu foil	R^2
Cu foam_as-prep.	0.06	0.84	0.10	1.000
Cu foam_-0.5V	0.03	1.01	-0.05	1.000
Cu foam_-0.7V	-0.01	1.24	-0.23	1.000
Cu foam_-0.9V	0.00	1.00	0.00	1.000
Cu_{rich}In_as-prep.	-0.05	1.63	-0.58	0.999
Cu_{rich}In_-0.6V	0.05	1.49	-0.57	0.995
Cu_{rich}In_-0.9V	0.17	0.73	0.08	0.997
Cu_{poor}In_as-prep.	0.35	-0.08	0.74	0.997
Cu_{poor}In_-0.6V	0.08	0.70	0.21	1.000
Cu_{poor}In_-0.9V	0.07	0.31	0.63	1.000
Cu_{rich}Sn_as-prep.	0.27	1.06	-0.34	0.999
Cu_{rich}Sn_-0.7V	0.09	1.49	-0.60	0.999
Cu_{rich}Sn_-0.9V	0.00	1.61	-0.63	1.000
Cu_{poor}Sn_as-prep.	0.40	0.22	0.37	0.996
Cu_{poor}Sn_-0.7V	0.37	1.16	-0.57	0.993
Cu_{poor}Sn_-0.9V	0.03	2.71	-1.78	0.995

Table S2. Cu XANES fit of samples using Cu_2O and Cu foil as references

	Ref_Cu_2O	Ref_Cu foil	R^2
Cu foam_as-prep.	0.23	0.79	0.997
Cu foam_-0.5V	0.24	0.78	0.996
Cu foam_-0.7V	0.24	0.78	0.994
Cu foam_-0.9V	0.20	0.82	0.996
Cu_{rich}In_as-prep.	0.28	0.75	0.988
Cu_{rich}In_-0.6V	0.36	0.65	0.986
Cu_{rich}In_-0.9V	0.32	0.68	0.995
Cu_{poor}In_as-prep.	0.33	0.67	0.997
Cu_{poor}In_-0.6V	0.23	0.78	0.998
Cu_{poor}In_-0.9V	0.13	0.88	0.999
Cu_{rich}Sn_as-prep.	0.49	0.53	0.995
Cu_{rich}Sn_-0.7V	0.39	0.62	0.990
Cu_{rich}Sn_-0.9V	0.33	0.69	0.989
Cu_{poor}Sn_as-prep.	0.45	0.56	0.996
Cu_{poor}Sn_-0.7V	0.61	0.38	0.988
Cu_{poor}Sn_-0.9V	0.58	0.42	0.966

Table S3. Cu XANES fit of samples using Cu_2O and Cu^0 as references

	Ref_Cu_2O	Ref_Cu^0	R^2
Cu foam_as-prep.	0.06	0.93	1.000
Cu foam_-0.5V	0.03	0.97	1.000
Cu foam_-0.7V	-0.02	1.02	1.000
Cu foam_-0.9V	/	/	/
$Cu_{rich}In$_as-prep.	-0.06	1.08	0.997
$Cu_{rich}In$_-0.6V	0.05	0.95	0.993
$Cu_{rich}In$_-0.9V	0.17	0.81	0.997
$Cu_{poor}In$_as-prep.	0.36	0.62	0.995
$Cu_{poor}In$_-0.6V	0.09	0.90	0.999
$Cu_{poor}In$_-0.9V	0.07	0.91	0.998
$Cu_{rich}Sn$_as-prep.	0.27	0.74	0.998
$Cu_{rich}Sn$_-0.7V	0.08	0.93	0.997
$Cu_{rich}Sn$_-0.9V	-0.01	1.02	0.998
$Cu_{poor}Sn$_as-prep.	0.41	0.58	0.995
$Cu_{poor}Sn$_-0.7V	0.36	0.62	0.992
$Cu_{poor}Sn$_-0.9V	0.01	1.02	0.980

Table S4. In XANES fit of samples using In_2O_3 and In foil as references

	Ref_In_2O_3	Ref_In foil	R^2
$Cu_{rich}In$_as-prep.	1.23	-0.24	0.993
$Cu_{rich}In$_-0.6V	0.23	0.76	0.997
$Cu_{rich}In$_-0.9V	0.26	0.72	0.995
$Cu_{poor}In$_as-prep.	0.84	0.15	0.996
$Cu_{poor}In$_-0.6V	0.33	0.65	0.995
$Cu_{poor}In$_-0.9V	0.91	0.06	0.977

Table S5. Sn XANES fit of samples using SnO_2 and Sn foil as references

	Ref_Sn foil	Ref_SnO_2	R^2
Cu_{rich}Sn_as-prep.	0.49	0.50	0.993
Cu_{rich}Sn_-0.7V	0.51	0.48	0.994
Cu_{rich}Sn_-0.9V	0.38	0.62	0.994
Cu_{poor}Sn_as-prep.	0.36	0.64	0.998
Cu_{poor}Sn_-0.7V	0.88	0.11	0.994
Cu_{poor}Sn_-0.9V	0.81	0.20	0.995

Table S6. Sn XANES fit of samples using SnO and Sn foil as references

	Ref_Sn foil	Ref_SnO	R^2
Cu_{rich}Sn_as-prep.	0.35	0.60	0.982
Cu_{rich}Sn_-0.7V	0.36	0.60	0.984
Cu_{rich}Sn_-0.9V	0.10	0.85	0.980
Cu_{poor}Sn_as-prep.	0.04	0.91	0.983
Cu_{poor}Sn_-0.7V	0.88	0.11	0.993
Cu_{poor}Sn_-0.9V	0.73	0.26	0.993

Table S7. Sn XANES fit of samples using three different references

	Ref_Sn foil	Ref_SnO	Ref_SnO_2	R^2
Cu_{rich}Sn_as-prep.	0.68	-0.29	0.61	0.994
Cu_{rich}Sn_-0.7V	0.66	-0.22	0.56	0.994
Cu_{rich}Sn_-0.9V	0.46	-0.11	0.65	0.994
Cu_{poor}Sn_as-prep.	0.40	-0.06	0.66	0.998
Cu_{poor}Sn_-0.7V	0.96	-0.13	0.16	0.994
Cu_{poor}Sn_-0.9V	0.85	-0.06	0.22	0.995

Table S8. Cu XANES edge energies according to the integral method

	Energy
Ref_Cu foil	8985.4
Cu foam_as-prep.	8984.2
Cu foam_-0.5V	8984.1
Cu foam_-0.7V	8983.8
Cu foam_-0.9V	8984.1
$Cu_{rich}In$_as-prep.	8983.3
$Cu_{rich}In$_-0.6V	8983.8
$Cu_{rich}In$_-0.9V	8984.6
$Cu_{poor}In$_as-prep.	8985.1
$Cu_{poor}In$_-0.6V	8984.5
$Cu_{poor}In$_-0.9V	8984.9
$Cu_{rich}Sn$_as-prep.	8983.7
$Cu_{rich}Sn$_-0.7V	8983.5
$Cu_{rich}Sn$_-0.9V	8983.4
$Cu_{poor}Sn$_as-prep.	8984.5
$Cu_{poor}Sn$_-0.7V	8983.9
$Cu_{poor}Sn$_-0.9V	8982.3

Table S9. Cu EXAFS: best fit models (see also Figure S8)

	N_{Cu1}	R_{Cu1}	N_{Cu2}	R_{Cu2}	N_{Cu3}	R_{Cu3}	N_{Cu4}	R_{Cu4}	N_{MS}	R_{MS}	$N_{In/Sn}$	$R_{In/Sn}$	ΔE_0	Rf
Cu foam_as-prep.	10.2(1)	2.538(1)	5.1#	3.581(4)	20.4#	4.415(2)	10.2#	5.100(3)	10.2#	2.563(2)			4.8	14.6
Cu foam_-0.5V	10.1(1)	2.535(1)	5.1#	3.574(4)	20.3#	4.414(2)	10.1#	5.093(3)	10.1#	2.56(2)			4.4	15.4
Cu foam_-0.7V	9.7(1)	2.534(1)	4.8#	3.579(4)	19.4#	4.413(2)	9.7#	5.093(3)	9.7#	2.563(2)			4.2	15.0
Cu foam_-0.9V	10.3(1)	2.536(1)	5.1#	3.577(3)	20.5#	4.414(2)	10.3#	5.095(3)	10.3#	2.56(2)			4.4	15.4
$Cu_{rich}In$_as-prep.	8.2(2)	2.531(2)	4.1#	3.568(5)	16.4#	4.411(3)	8.2#	5.083(4)	8.2#	2.558(2)	1.0(2)	2.674(8)	2.8	12.7
$Cu_{rich}In$_-0.6V	8.0(1)	2.532(2)	4.0#	3.592(5)	16.0#	4.403(3)	8.0#	5.068(5)	8.0#	2.557(3)	0.8(2)	2.761(12)	2.1	20.9
$Cu_{rich}In$_-0.9V	7.8(2)	2.529(2)	3.9#	3.574(5)	15.6#	4.406(3)	7.8#	5.077(5)	7.8#	2.56(3)	0.9(2)	2.688(8)	2.6	17.8
$Cu_{poor}In$_as-prep.	8.1(1)	2.532(3)	3.3#	3.576(6)	13.2#	4.410(3)	6.6#	5.076(6)	6.6#	2.562(3)	0.8(2)	2.735(12)	3.6	20.4
$Cu_{poor}In$_-0.6V	9.3(2)	2.530(2)	4.6#	3.585(4)	18.5#	4.404(2)	9.3#	5.088(3)	9.3#	2.559(2)	1.3(2)	2.668(7)	3.3	14.2
$Cu_{poor}In$_-0.9V	10.9(2)	2.528(2)	5.4#	3.575(3)	21.7#	4.406(2)	10.9#	5.084(3)	10.9#	2.563(2)	1.5(2)	2.671(5)	3.5	14.4
$Cu_{rich}Sn$_as-prep.	5.8(2)	2.548(3)	1.5(3)	3.586(12)	5.1(6)	4.444(7)	7.5(9)	5.242(7)			0.8(2)	2.802(11)	3.6	21.3
$Cu_{rich}Sn$_-0.7V	5.5(2)	2.508(4)	1.8(3)	3.551(10)	8.4(6)	4.382(6)	12.1(9)	5.191(6)			2.1(3)	2.661(5)	-2.8	18.5
$Cu_{rich}Sn$_-0.9V	6.1(2)	2.518(4)	1.8(3)	3.565(10)	8.6(6)	4.402(5)	13.0(9)	5.210(6)			1.5(3)	2.665(6)	0.0	21.0
$C_{poor}Sn$_as-prep.	5.0(2)	2.534(5)	0.9(3)	3.568(19)	2.7(6)	4.42(12)	5.4(9)	5.230(10)			0.6(2)	2.744(19)	2.5	17.8
$Cu_{poor}Sn$_-0.7V	2.9(2)	2.511(12)	0.6(3)	3.552(32)	1.7(6)	4.388(21)	3.2(9)	5.201(19)			1.2(3)	2.692(15)	2.3	12.3
$Cu_{poor}Sn$_-0.9V	3.8(2)	2.517(7)	0.5(3)	3.589(35)	1.9(6)	4.393(18)	4.3(9)	5.215(14)			0.9(2)	2.718(16)	1.8	14.9

Best fitting for all samples: 2b for Cu foam and Cu-In foam, 6b for Cu-Sn foam. $2\sigma^2$ set to 0.007 (σ=0.059), *fixed parameter, #constrained parameter, N: coordination number, N/R_{MS}: multiple scattering shell, R: shell distance, ΔE_0: energy offset, Rf: fit R-factor

Table S10. Cu EXAFS: All four fit models for Cu foam in the as-prepared state

model	N_{Cu1}	R_{Cu1}	σ_{cu1}	N_{Cu2}	R_{Cu2}	σ_{cu2}	N_{Cu3}	R_{Cu3}	σ_{cu3}	N_{Cu4}	R_{Cu4}	σ_{cu4}	N_{MS}	R_{MS}	σ_{MS}	Rf
**1**	12.0*	2.545(1)	0.063(1)	6.0*	3.592(3)	0.061(2)	24.0*	4.419(2)	0.061(2)	12.0*	5.091#	0.064(1)	12.0*	2.545#	0.064#	19.8
**2**	10.3(3)	2.545(1)	0.060(1)	5.2#	3.585(4)	0.060#	20.7#	4.416(2)	0.060#	10.3#	5.090#	0.060#	10.3#	2.545#	0.060#	20.3
**2a**	10.1(1)	2.545(1)	0.059*	5.1#	3.585(4)	0.059*	20.2#	4.415(2)	0.059*	10.1#	5.090#	0.059*	10.1#	2.545#	0.059*	20.2
**2b**	10.2(1)	2.538(1)	0.059*	5.1#	3.581(4)	0.059*	20.4#	4.414(2)	0.059*	10.2#	5.100(3)	0.059*	10.2#	2.562(2)	0.059*	14.5

Models for Cu foam_as-prepared. Best model (2b) highlighted in blue color. *fixed parameter, #constrained parameter

N: coordination number, N/R_{MS}: multiple scattering shell, R: shell distance, σ: Debye-Waller parameter, Rf: fit R-factor

Table S11. Cu EXAFS: All 13 fit models for Cu rich Cu-In foam in the as-prepared state

Models for Cu rich Cu-In foam as-prepared. Best model (2b) highlighted in blue color. *fixed parameter, #constrained parameter

Model	N_{Cu1}	R_{Cu1}	σ_{cu1}	N_{Cu2}	R_{Cu2}	σ_{cu2}	N_{Cu3}	R_{Cu3}	σ_{cu3}	N_{Cu4}	R_{Cu4}	σ_{cu4}	N_{MS}	R_{MS}	σ_{MS}	N_{In}	R_{In}	σ_{In}	Rf
**1**	12.0*	2.542(1)	0.069(1)	6.0*	3.574(5)	0.077(3)	24.0*	4.418(3)	0.077(3)	12.0*	5.084#	0.073(1)	12.0*	2.542#	0.073#				20.1
**2**	9.9(4)	2.545(2)	0.068(2)	5.0#	3.573(5)	0.068#	19.8#	4.417(3)	0.068#	9.9#	5.089#	0.068#	9.9#	2.545#	0.068#	1.9(3)	2.623(6)	0.068#	14.1
**2a**	8.2(2)	2.544(1)	0.059*	4.1#	3.575(5)	0.059*	16.5#	4.417(3)	0.059*	8.2#	5.088#	0.059*	8.2#	2.544#	0.059*	1.1(2)	2.619(8)	0.059*	15.1
**2b**	8.2(2)	2.531(2)	0.059*	4.1#	3.568(5)	0.059*	16.4#	4.411(3)	0.059*	8.2#	5.083(5)	0.059*	8.2#	2.558(3)	0.059*	1.0(2)	2.674(9)	0.059*	12.7
**3**	10.2(4)	2.545(2)	0.069(2)	5.1#	3.574(5)	0.069#	20.4#	4.418(3)	0.069#	10.2#	5.090#	0.069#	10.2#	2.545#	0.069#	1.4(4)	2.624(5)	0.054(10)	13.2
**3a**	8.2(2)	2.544(1)	0.059*	4.1#	3.575(5)	0.059*	16.5#	4.417(3)	0.059*	8.2#	5.088#	0.059*	8.2#	2.544#	0.059*	1.2(4)	2.619(8)	0.063(12)	15.2
**4**	8.7(3)	2.529(2)	0.057(2)	3.9(4)	3.559(5)	0.057#	14.2(8)	4.405(3)	0.057#	19.2(12)	5.21(3)	0.057#							21.7
**5**	10.5(6)	2.521(2)	0.059(2)	4.0(4)	3.550(5)	0.059#	14.5(10)	4.393(3)	0.059#	22.0(15)	5.196(4)	0.059#				1.6(3)	2.352(10)	0.059#	19.5
**6**	6.7(4)	2.506(3)	0.054(3)	3.4(4)	3.538(5)	0.054#	12.7(11)	4.384(4)	0.054#	20.0(18)	5.186(4)	0.054#				2.3(3)	2.671(6)	0.054#	19.8
**6a**	7.4(2)	2.508(2)	0.059*	3.8(4)	3.539(5)	0.059*	14.4(6)	4.384(2)	0.059*	22.9(10)	5.188(2)	0.059*				2.5(2)	2.664(3)	0.059*	20.2
**7**	7.0(4)	2.505(2)	0.052(3)	3.1(4)	3.539(5)	0.052#	12.5(10)	4.384(2)	0.052#	19.5(17)	5.188(2)	0.052#				1.2(4)	2.676(5)	0.011(24)	19.0
**7a**	7.6(3)	2.506(3)	0.059*	3.6(4)	3.538(6)	0.059*	14.4(6)	4.383(4)	0.059*	23.1(10)	5.187(4)	0.059*				1.5(4)	2.667(4)	0.032(15)	19.2
**8**	8.3(4)	2.518(2)	0.059*(3)	3.8(4)	3.549(5)	0.059*	14.6(11)	4.394(3)	0.059*	21.5(17)	5.199(3)	0.059*				0.8#	2.676(10)	0.039*	19.7

N: coordination number, N/R_{MS}: multiple scattering shell, R: shell distance, σ: Debye-Waller parameter, Rf: fit R-factor. For model 5, an extra Cu replaces In shell， N_{In},R_{In} and σ_{In} are replaced by N_{Cu},R_{Cu} and σ_{Cu}

Table S12. Cu EXAFS: All 13 fit models for Cu rich Cu-Sn foam in the as-prepared state

model	N_{Cu1}	R_{Cu1}	σ_{cu1}	N_{Cu2}	R_{Cu2}	σ_{cu2}	N_{Cu3}	R_{Cu3}	σ_{cu3}	N_{Cu4}	R_{Cu4}	σ_{cu4}	N_{MS}	R_{MS}	σ_{MS}	N_{Sn}	R_{Sn}	σ_{Sn}	Rf
1	12.0*	2.553(2)	0.087(0)	6.0*	3.587(7)	0.087*	24.0*	4.44(4)	0.087*	12.0*	5.107#	0.087*	12.0*	2.553#	0.087#				48.6
2	6.2(5)	2.549(3)	0.076(3)	3.1#	3.58(10)	0.076#	12.4#	4.433(6)	0.076#	6.2#	5.099#	0.076#	6.2#	2.549#	0.076#	1.7(4)	2.636(8)	0.076#	28.7
2a	4.2(2)	2.548(3)	0.059*	2.1#	3.587(10)	0.059*	8.5#	4.435(5)	0.059*	4.2#	5.095#	0.059*	4.2#	2.548#	0.059*	0.7(2)	2.635(12)	0.059*	31.3
2b	4.5(2)	2.533(5)	0.059*	2.3#	3.574(9)	0.059*	9.0#	4.427(6)	0.059*	4.5#	5.073(11)	0.059*	4.5#	2.568(6)	0.059*	0.8(2)	2.682(10)	0.059*	29.8
3	7.7(6)	2.557(4)	0.085(3)	3.8#	3.591(11)	0.085#	15.3#	4.443(7)	0.085#	7.7#	5.115#	0.085#	7.7#	2.557#	0.085#	0.9(1)	2.629(5)	0.039*	24.8
3a	4.2(2)	2.544(3)	0.059*	2.1#	3.582(10)	0.059*	8.5#	4.432(5)	0.059*	4.2#	5.088#	0.059*	4.2#	2.544#	0.059*	1.7(4)	2.664(12)	0.087*	31.5
4	6.0(4)	2.543(3)	0.063(3)	1.4(4)	3.578(15)	0.063#	5.6(8)	4.435(8)	0.063#	8.9(13)	5.235(8)	0.063#							23.5
5	8.4(9)	2.529(4)	0.073(3)	1.6(5)	3.568(17)	0.073#	7(11)	4.422(9)	0.073#	12.4(18)	5.224(9)	0.073#				1.5(5)	2.324(10)	0.073#	22.1
6	6.1(5)	2.549(3)	0.062(4)	1.5(4)	3.588(14)	0.062#	5.4(8)	4.445(8)	0.062#	7.9(13)	5.244(8)	0.062#				0.8(2)	2.812(16)	0.062#	21.5
**6a**	**5.8(2)**	**2.548(3)**	**0.059***	**1.5(4)**	**3.586(13)**	**0.059***	**5.1(6)**	**4.444(8)**	**0.059***	**7.5(10)**	**5.241(8)**	**0.059***				**0.8(2)**	**2.801(13)**	**0.059***	**21.2**
7	5.5(5)	2.542(4)	0.057(4)	1.4(4)	3.582(13)	0.057#	4.8(7)	4.439(8)	0.057#	7.4(12)	5.236(8)	0.057#				2(4)	2.776(17)	0.087*	20.3
7a	5.8(2)	2.543(4)	0.059*	1.5(4)	3.583(14)	0.059*	5.1(6)	4.439(8)	0.059*	7.7(10)	5.238(8)	0.059*				1.8(4)	2.783(15)	0.087*	20.5
8	6.2(5)	2.548(4)	0.063*(4)	1.5(4)	3.586(15)	0.063*	5.5(8)	4.443(8)	0.063*	8.2(14)	5.242(9)	0.063*				0.4#	2.815(25)	0.055(17)	22.2

Models for Cu rich Cu-In foam as-prepared. Best model (6a) highlighted in blue color. *fixed parameter, #constrained parameter

N: coordination number, N/R_{MS}: multiple scattering shell, R: shell distance, σ: Debye-Waller parameter, Rf: fit R-factor. For model 5, an extra Cu replaces Sn shell， N_{Sn},R_{Sn} and σ_{Sn} are replaced by N_{Cu},R_{Cu} and σ_{Cu}

Table S13. Summary of the XRD, XPS and XAS findings

Catalyst material	Information obtained	Technique
**$Cu_{85}In_{15}$** (Cu-rich Cu-In foams)	- High CO-selectivity	Electrochemistry/GC measurements
	- No crystalline alloy<br>- Bulk composed of metallic In and Cu species with small contribution from Cu oxides (Cu_2O)	ex situ GI-XRD analysis
	- No electronic interaction.<br>- At OCP, surface composed of oxidized Cu and In species with tiny contribution from metallic Cu.<br>- Under bias, surface showed mainly surface metallic Cu and In species with traces of In oxides.	ex situ and quasi in situ XPS measurements
	- Cu-In amorphous alloy formed under CO2ER conditions.<br>- Oxidic In phases (partially) reduced and some of the In atoms are incorporated into the Cu-In alloy during CO2ER.<br>- Showed high degree of crystallinity.	quasi in situ XAS measurements
**$Cu_{25}In_{75}$** (In-rich Cu-In foams)	- High selectivity towards HCOO-	Electrochemistry/GC measurements
	- No crystalline alloy.<br>- Bulk composed of exclusively metallic In and Cu species.	ex situ GI-XRD analysis
	- No electronic interaction<br>- Showed exclusively surface Cu and In metallic species regardless the applied potential.	ex situ and quasi in situ XPS measurements
	- Indication of amorphous Cu-In alloy formation<br>- Suggesting core-shell like structure with Cu-In alloy core and InOx shell<br>- Indication of In migration from bulk into surface under bias	quasi in situ XAS measurements
**$Cu_{85}Sn_{15}$** (Cu-rich Cu-Sn foams)	- High CO-selectivity	Electrochemistry/GC measurements
	- No crystalline alloy<br>- Showed only Cu and Cu_2O reflection peaks	ex situ GI-XRD analysis
	- No electronic interaction<br>- Exhibited metallic Cu and mainly oxidized Sn surface species under bias	ex situ and quasi in situ XPS measurements
	- Indicate the formation of amorphous Cu-Sn alloy<br>- Suggest co-existence of separate SnOx (as shell) and Cu-In alloy (core) phases.<br>- Suggest In migration from bulk into surface under bias	quasi in situ XAS measurements
**$Cu_{40}Sn_{60}$**	- High selectivity for HCOO-	Electrochemistry/GC measurements
	- No crystalline alloy<br>- Bulk composed of exclusively metallic Sn and Cu phases	ex situ GI-XRD analysis
	- No electronic interaction<br>- Sn surface speciation depend on the applied bias.<br>- At high cathodic bias, surface composed exclusively from metallic Cu and Sn species.	ex situ and quasi in situ XPS measurements
	- Indicate the formation of amorphous Cu-Sn alloy<br>- Suggest co-existence of separate SnOx (as shell) and Cu-In alloy (core) phases.<br>- Suggest In migration from bulk into surface under bias	quasi in situ XAS measurements

References

1. Klingan, K.; Kottakkat, T.; Jovanov, Z. P.; Jiang, S.; Pasquini, C.; Scholten, F.; Kubella, P.; Bergmann, A.; Roldan Cuenya, B.; Roth, C.; Dau, H., *ChemSusChem* **2018,** 11 (19), 3449-3459.
2. Kottakkat, T.; Klingan, K.; Jiang, S.; Jovanov, Z. P.; Davies, V. H.; El-Nagar, G. A. M.; Dau, H.; Roth, C., *ACS Applied Materials & Interfaces* **2019,** 11 (16), 14734-14744.
3. Rahaman, M.; Kiran, K.; Zelocualtecatl Montiel, I.; Dutta, A.; Broekmann, P., *ACS Applied Materials & Interfaces* **2021,** 13 (30), 35677-35688.
4. Stojkovikj, S.; El-Nagar, G. A.; Firschke, F.; Pardo Pérez, L. C.; Choubrac, L.; Najdoski, M.; Mayer, M. T., *ACS Applied Materials & Interfaces* **2021,** 13 (32), 38161-38169.
5. Pardo Pérez, L. C.; Arndt, A.; Stojkovikj, S.; Ahmet, I. Y.; Arens, J. T.; Dattila, F.; Wendt, R.; Guilherme Buzanich, A.; Radtke, M.; Davies, V.; Höflich, K.; Köhnen, E.; Tockhorn, P.; Golnak, R.; Xiao, J.; Schuck, G.; Wollgarten, M.; López, N.; Mayer, M. T., *Advanced Energy Materials* **2022,** 12 (5), 2103328.
6. Biesinger, M. C., *Surface and Interface Analysis* **2017,** 49 (13), 1325-1334.
7. Dau, H.; Liebisch, P.; Haumann, M., *Analytical and Bioanalytical Chemistry* **2003,** 376 (5), 562-583.
8. Rehr, J. J.; Kas, J. J.; Vila, F. D.; Prange, M. P.; Jorissen, K., *Physical Chemistry Chemical Physics* **2010,** 12 (21), 5503-5513.
9. Jiang, S.; Klingan, K.; Pasquini, C.; Dau, H., *The Journal of Chemical Physics* **2019,** 150 (4), 041718.
